# Supplementary material for: Identifying MMP14 and COL12A1 as a potential combination of prognostic biomarkers in pancreatic ductal adenocarcinoma using integrated bioinformatics analysis
Source: PeerJ. 2020 Nov 23;8:e10419. doi: 10.7717/peerj.10419 (PMC7690310; doi:10.7717/peerj.10419)
Supplement: File S1 [file peerj-08-10419-s001.docx]

**Supplemental files**

Data Availability

The following information was supplied regarding data availability:

Data is available at NCBI GEO: GSE28735, GSE62165, GSE91035.
