# Supplementary material for: Identifying MMP14 and COL12A1 as a potential combination of prognostic biomarkers in pancreatic ductal adenocarcinoma using integrated bioinformatics analysis
Source: PeerJ. 2020 Nov 23;8:e10419. doi: 10.7717/peerj.10419 (PMC7690310; doi:10.7717/peerj.10419)
Supplement: Table S1 [file peerj-08-10419-s002.docx]

Supplementary Table.1 DEGs in the identification of three datasets

| Dataset | DEGs |
| --- | --- |
| GSE28735 | 22028 |
| GSE62165 | 49384 |
| GSE91035 | 28898 |
