# Supplementary figures and images for: Identifying MMP14 and COL12A1 as a potential combination of prognostic biomarkers in pancreatic ductal adenocarcinoma using integrated bioinformatics analysis
Source: PeerJ. 2020 Nov 23;8:e10419. doi: 10.7717/peerj.10419 (PMC7690310; doi:10.7717/peerj.10419)

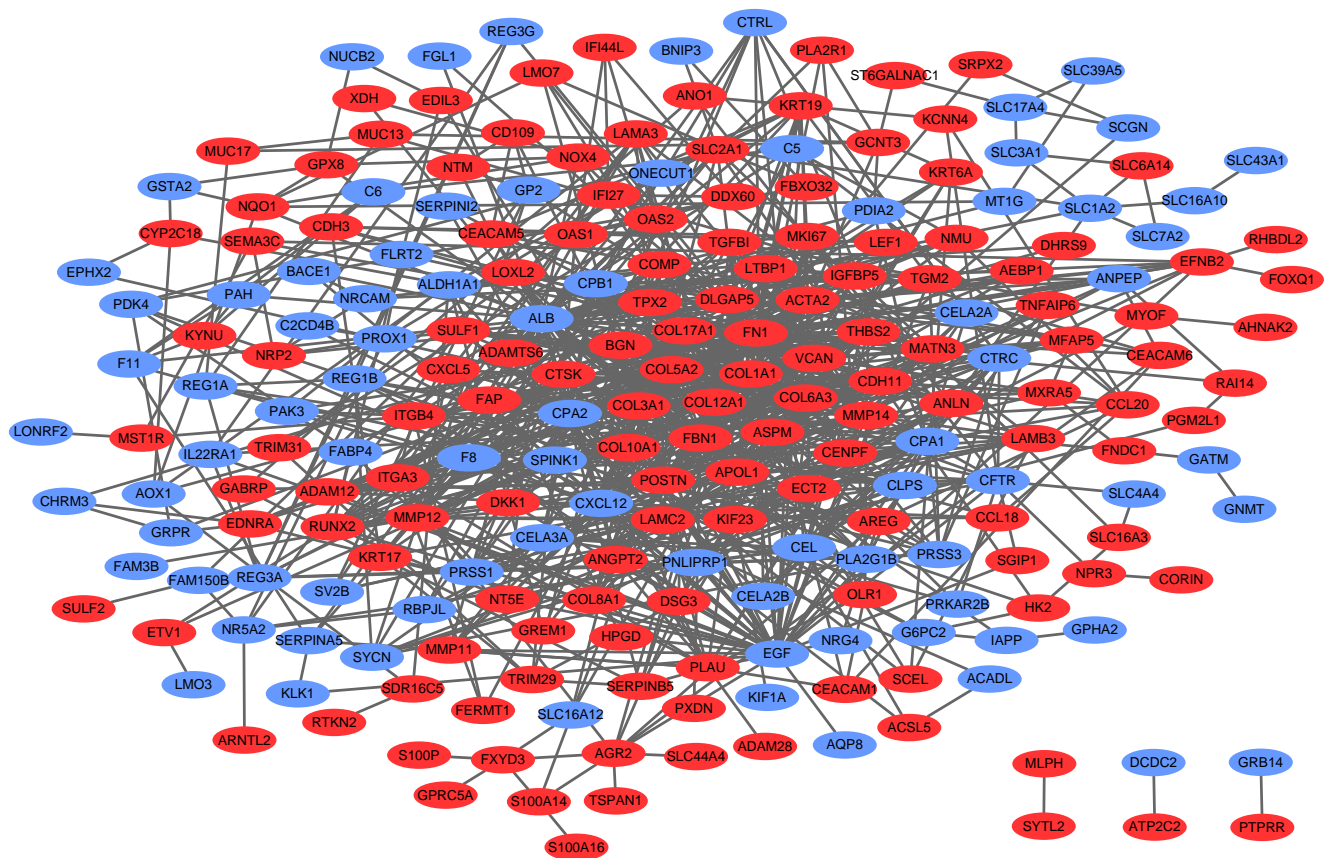

Supplement: Supplemental Information 4 [file peerj-08-10419-s004.pdf]

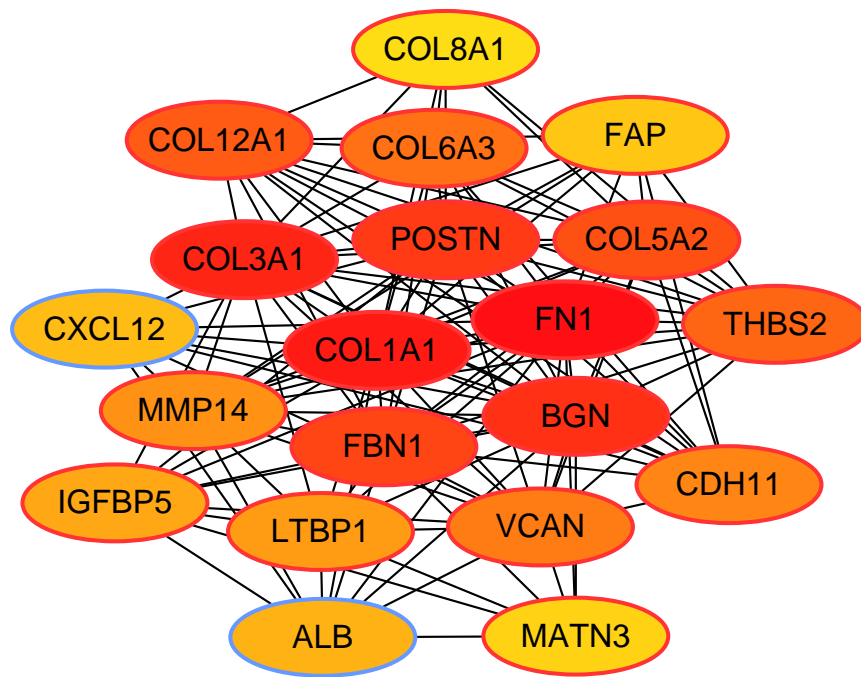

Supplement: Supplemental Information 5 [file peerj-08-10419-s005.pdf]

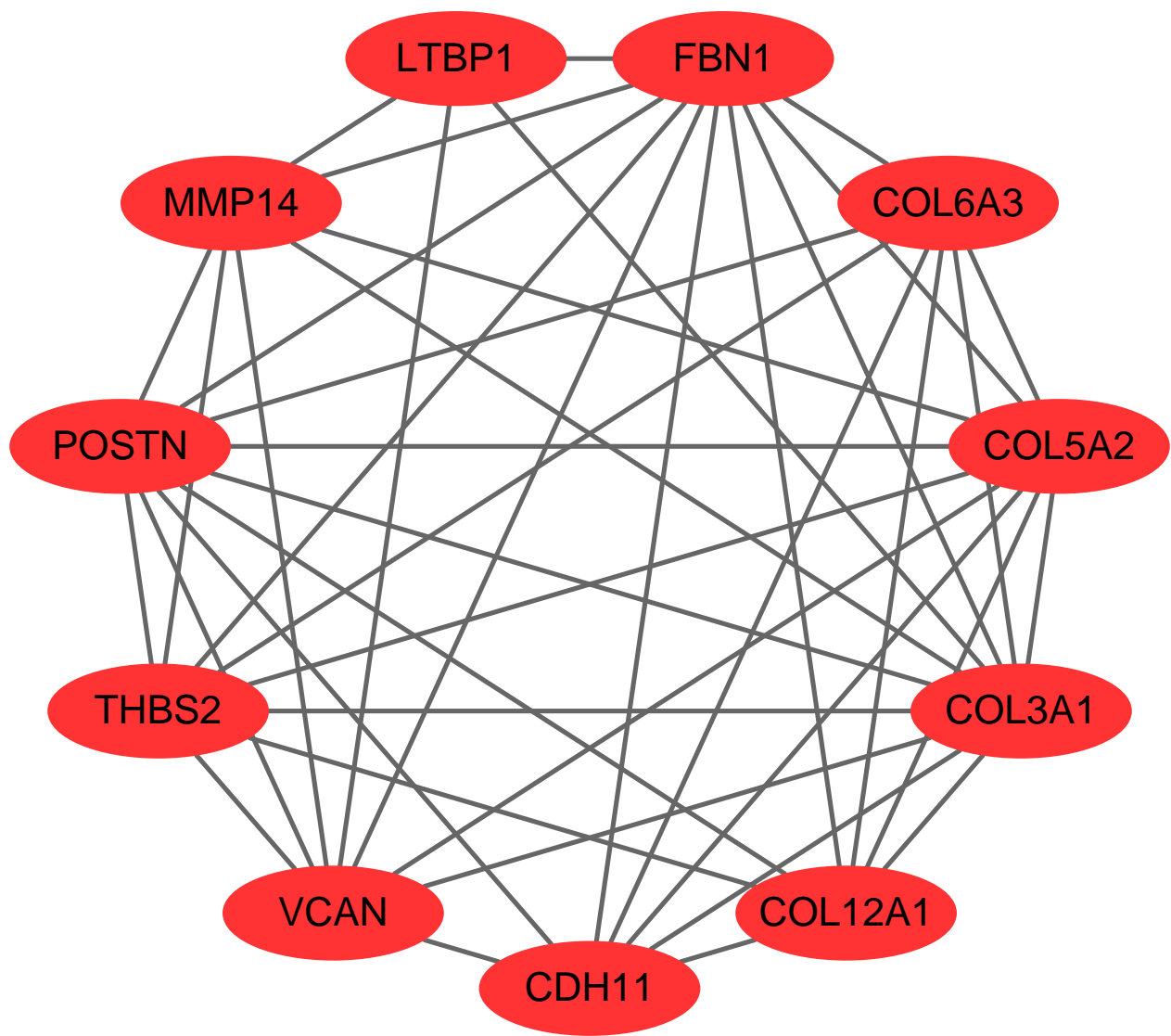

Supplement: Supplemental Information 6 [file peerj-08-10419-s006.pdf]

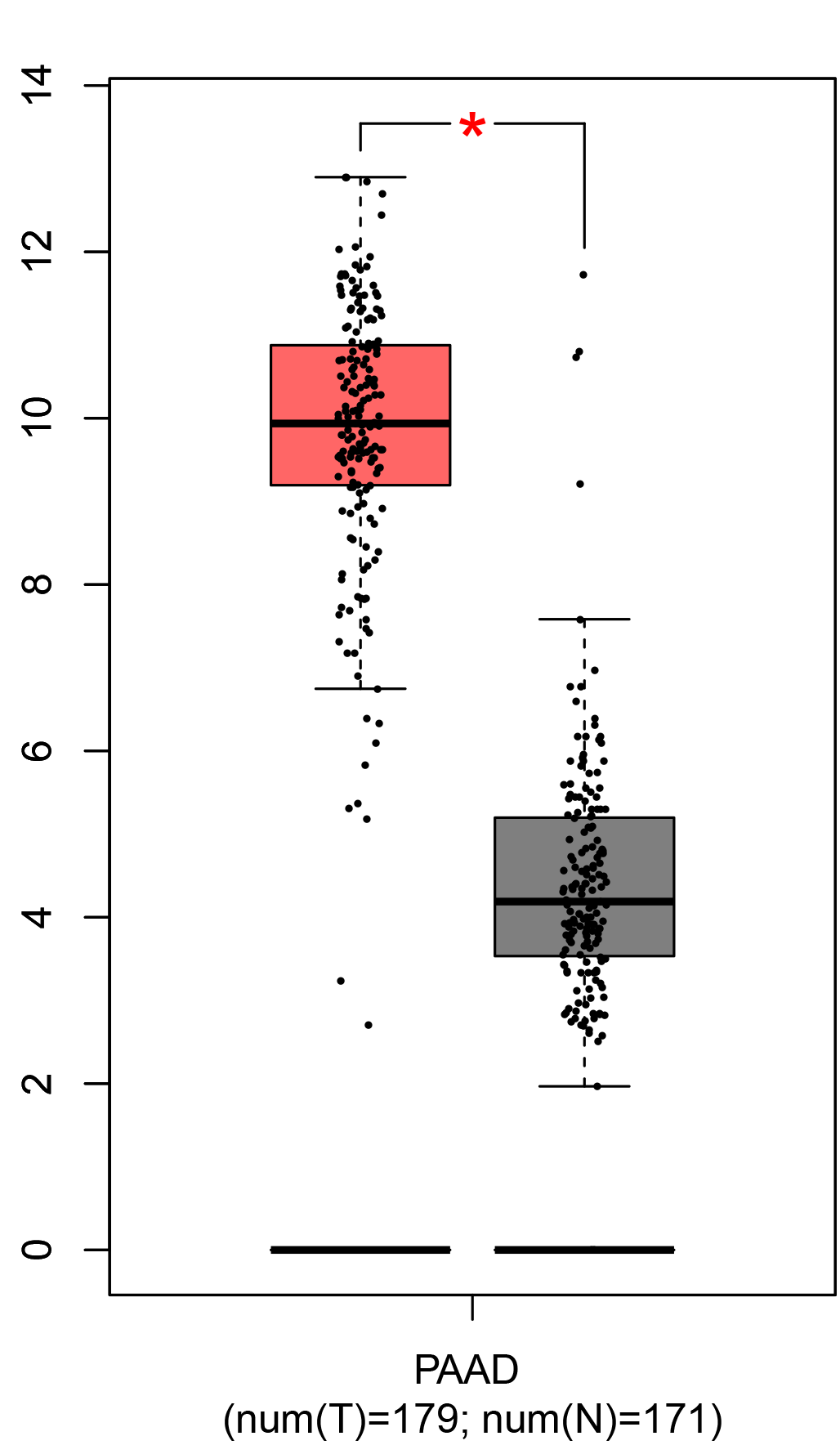

Supplement: Supplemental Information 7 [file peerj-08-10419-s007.png]

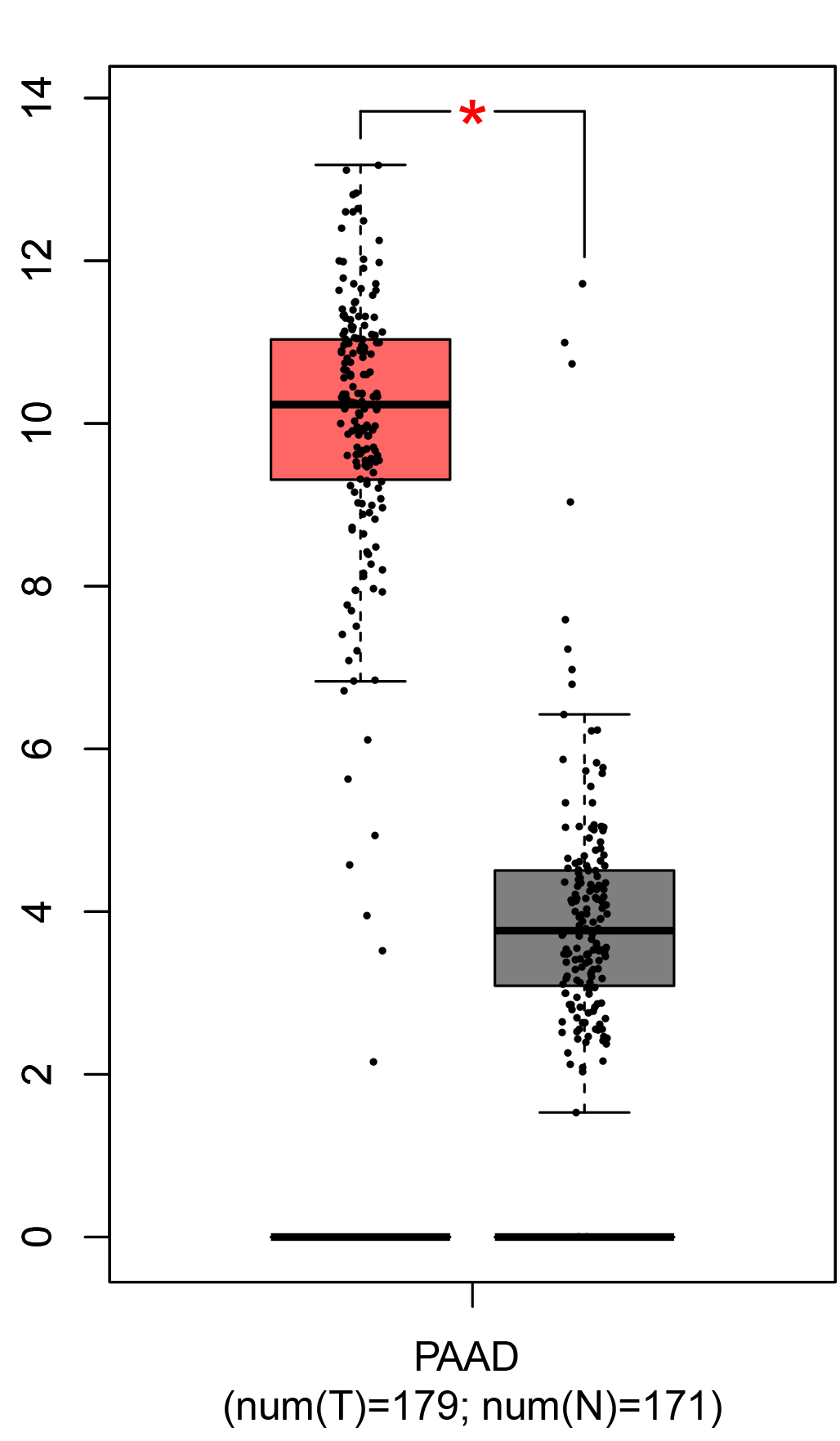

Supplement: Supplemental Information 8 [file peerj-08-10419-s008.png]

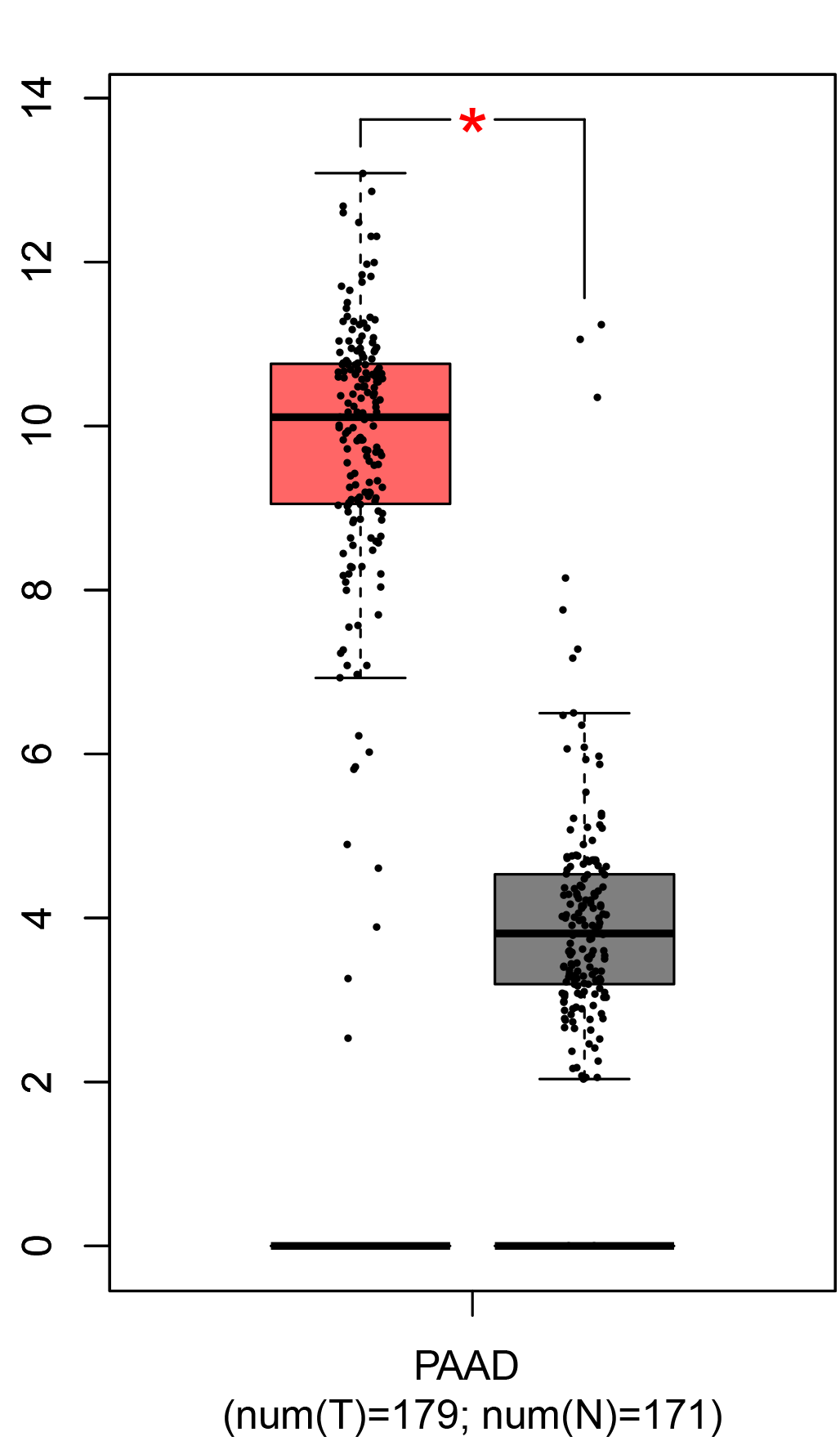

Supplement: Supplemental Information 9 [file peerj-08-10419-s009.png]

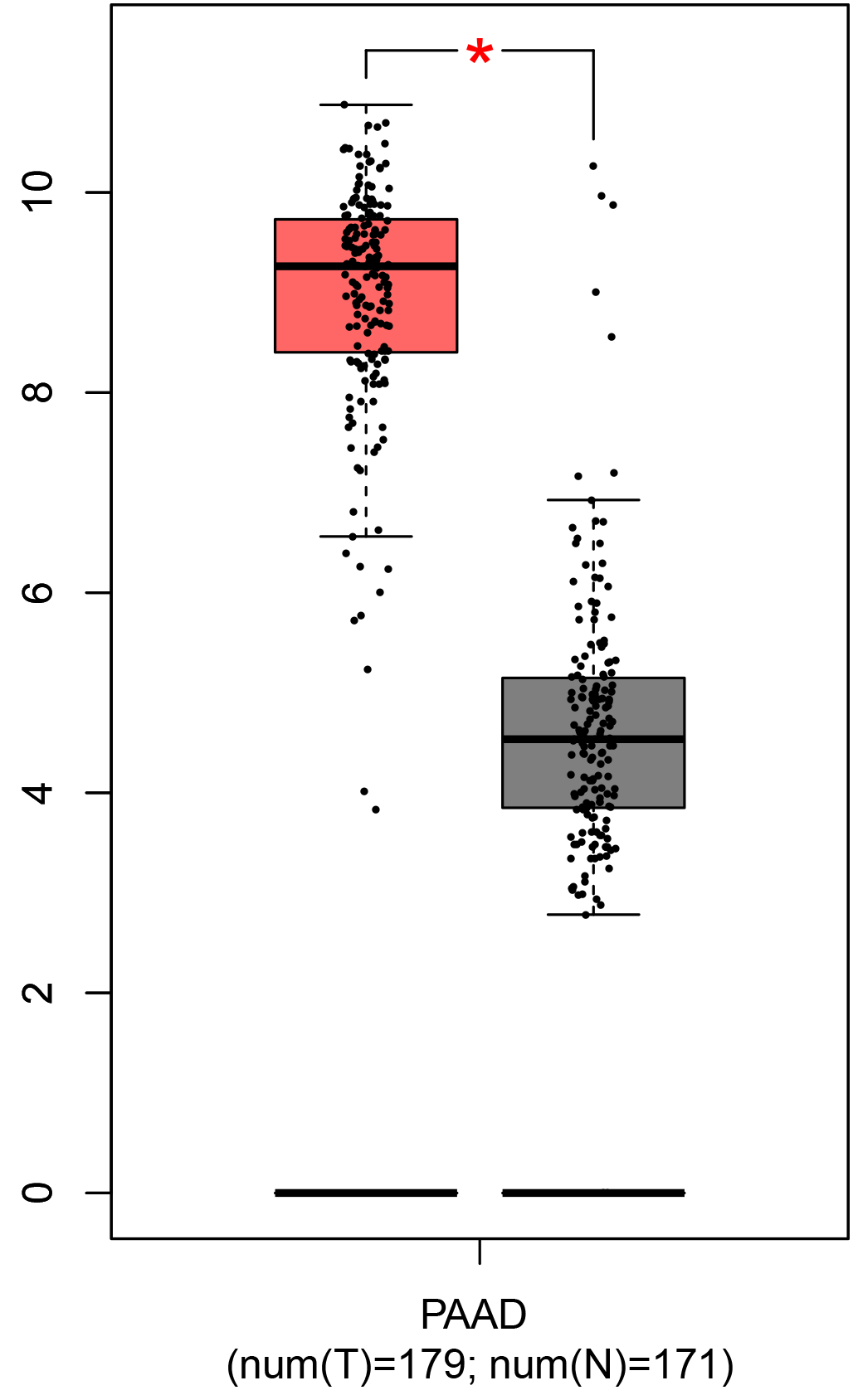

Supplement: Supplemental Information 10 [file peerj-08-10419-s010.png]

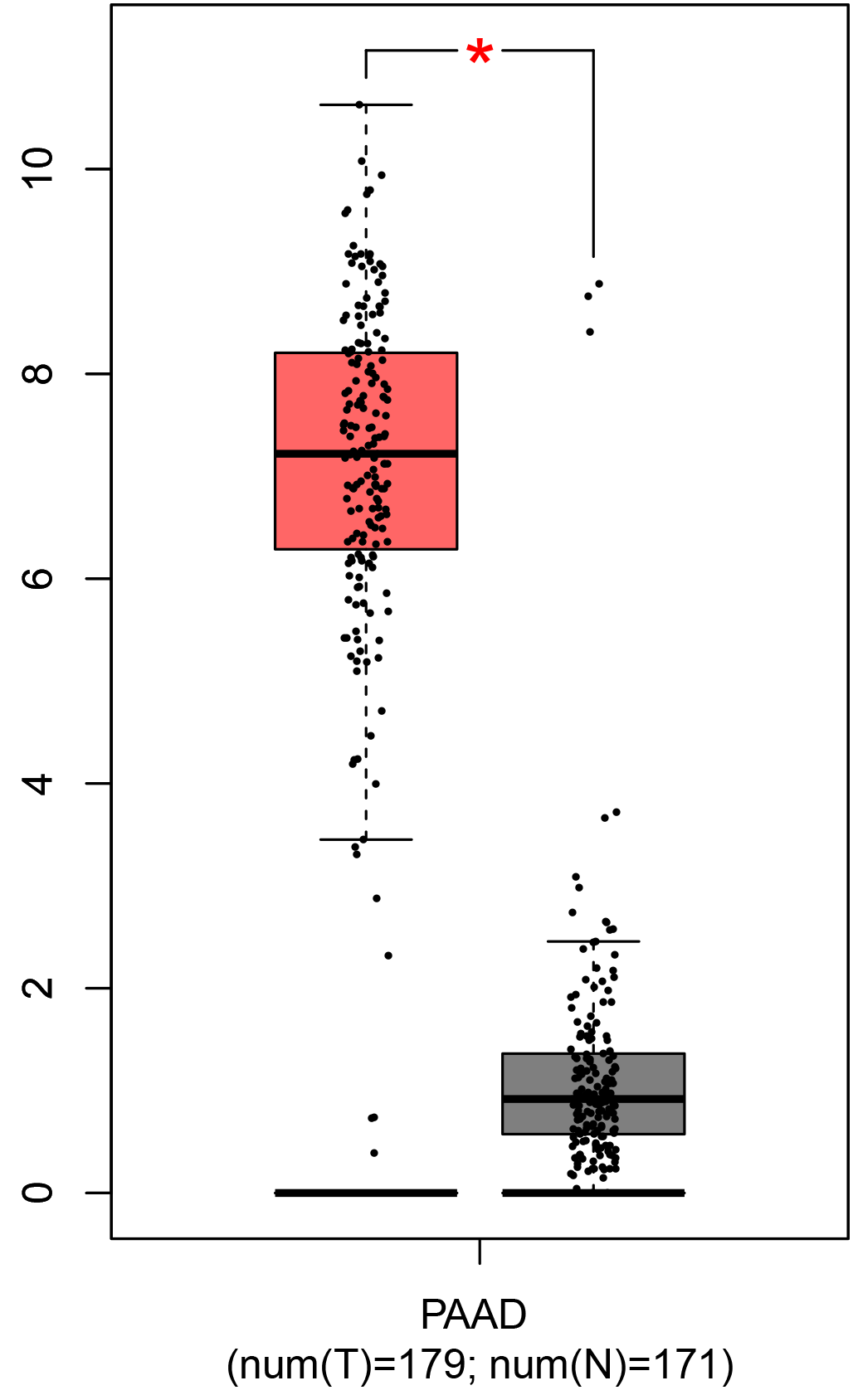

Supplement: Supplemental Information 11 [file peerj-08-10419-s011.png]

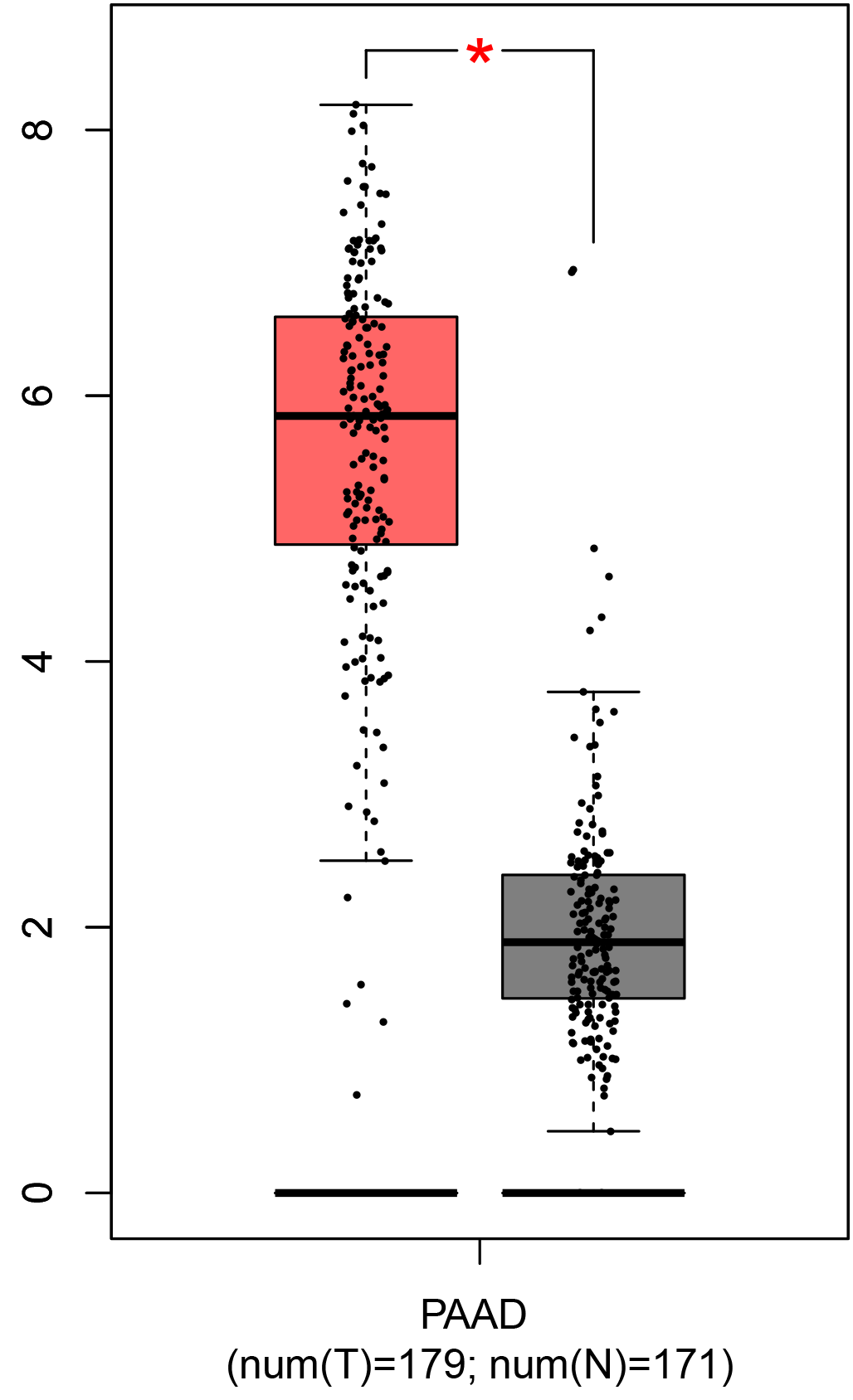

Supplement: Supplemental Information 12 [file peerj-08-10419-s012.png]

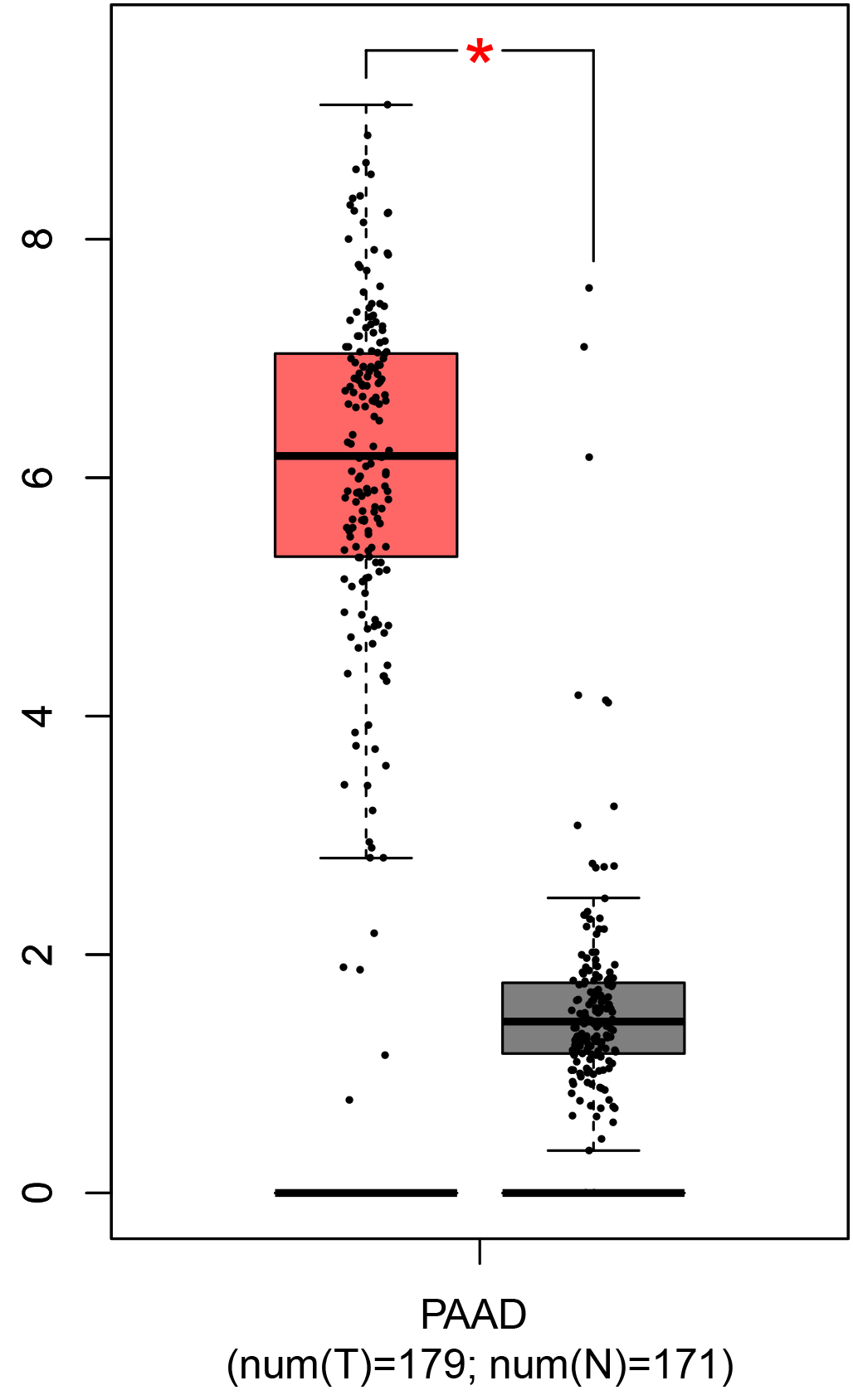

Supplement: Supplemental Information 13 [file peerj-08-10419-s013.png]

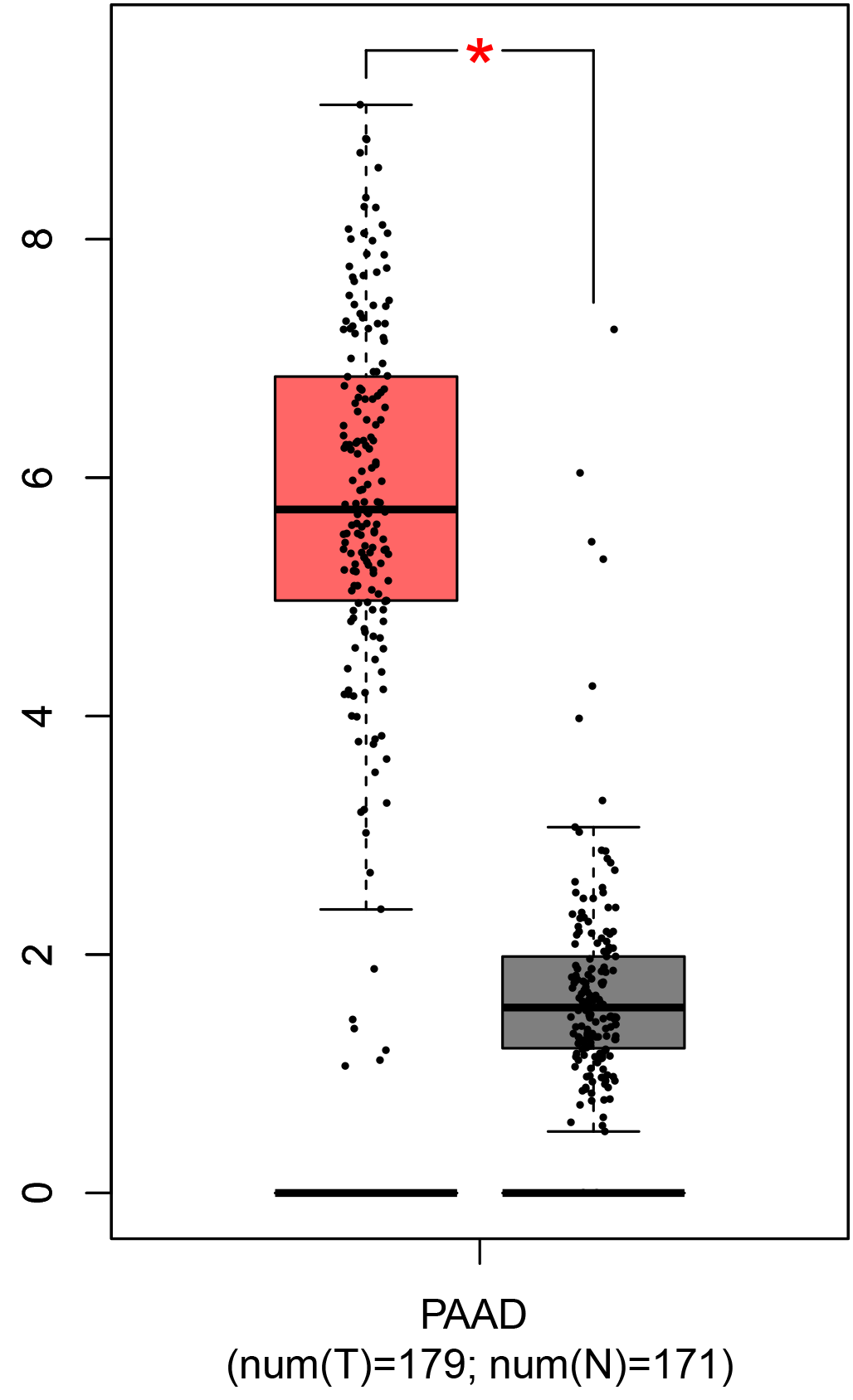

Supplement: Supplemental Information 14 [file peerj-08-10419-s014.png]

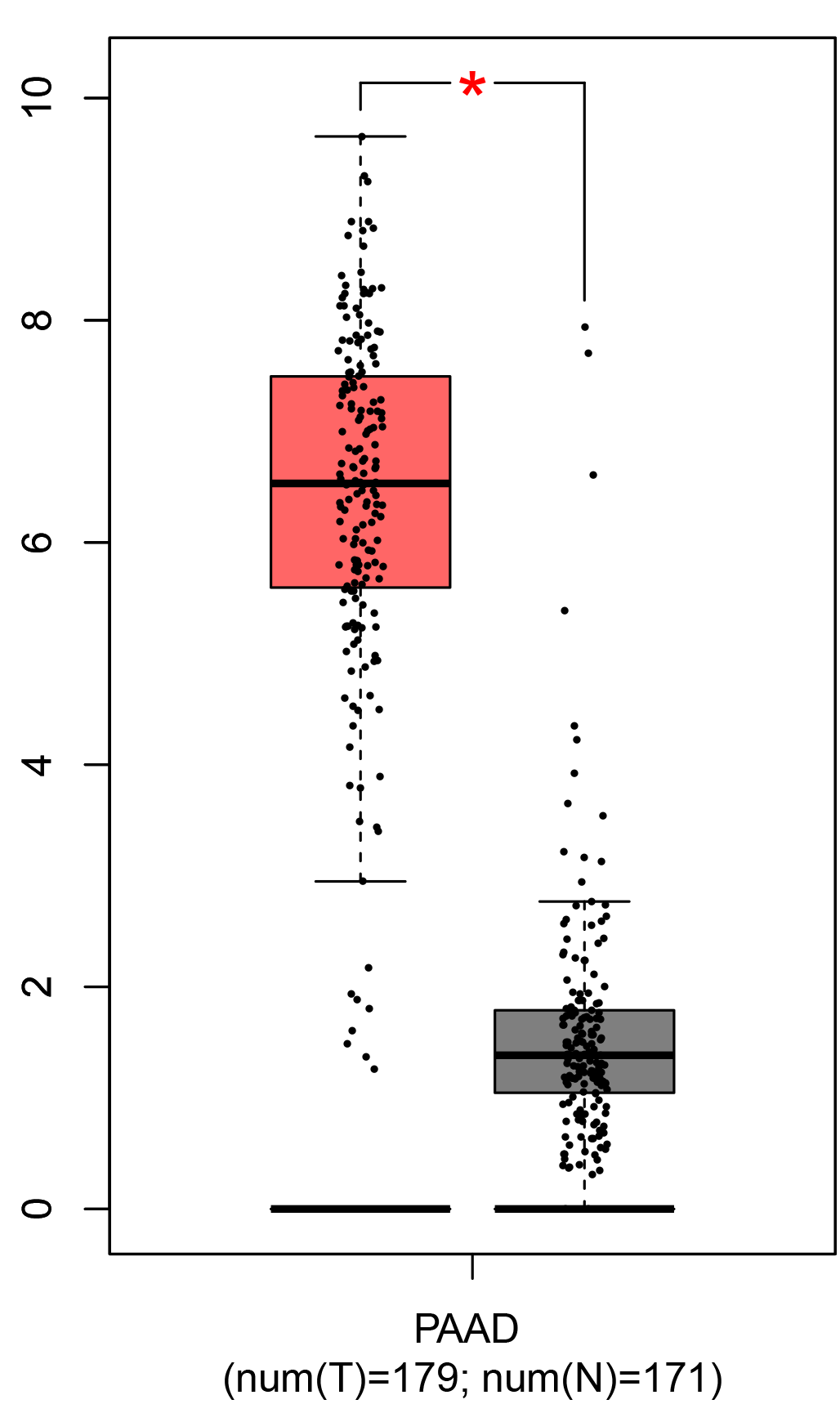

Supplement: Supplemental Information 15 [file peerj-08-10419-s015.png]

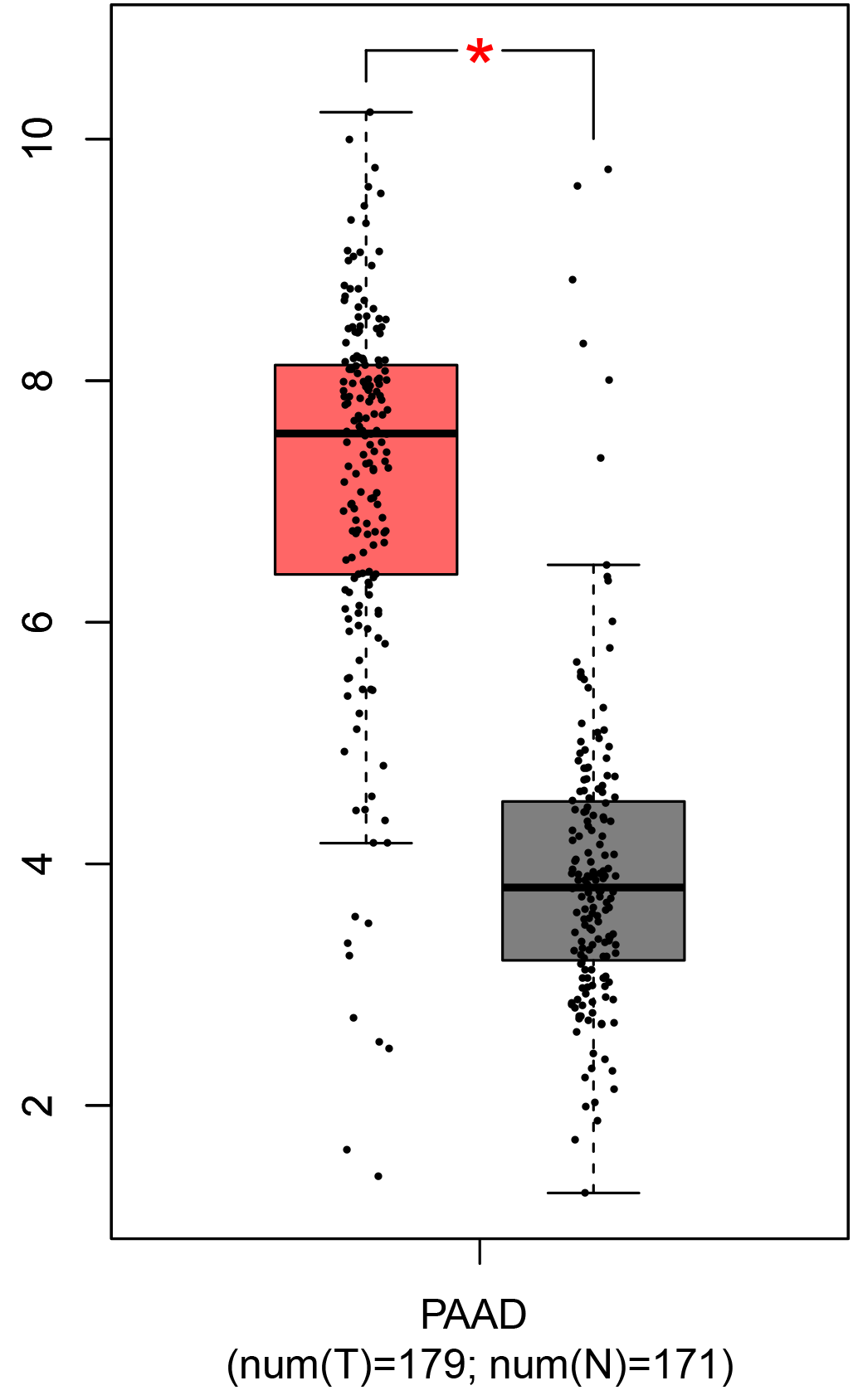

Supplement: Supplemental Information 16 [file peerj-08-10419-s016.png]

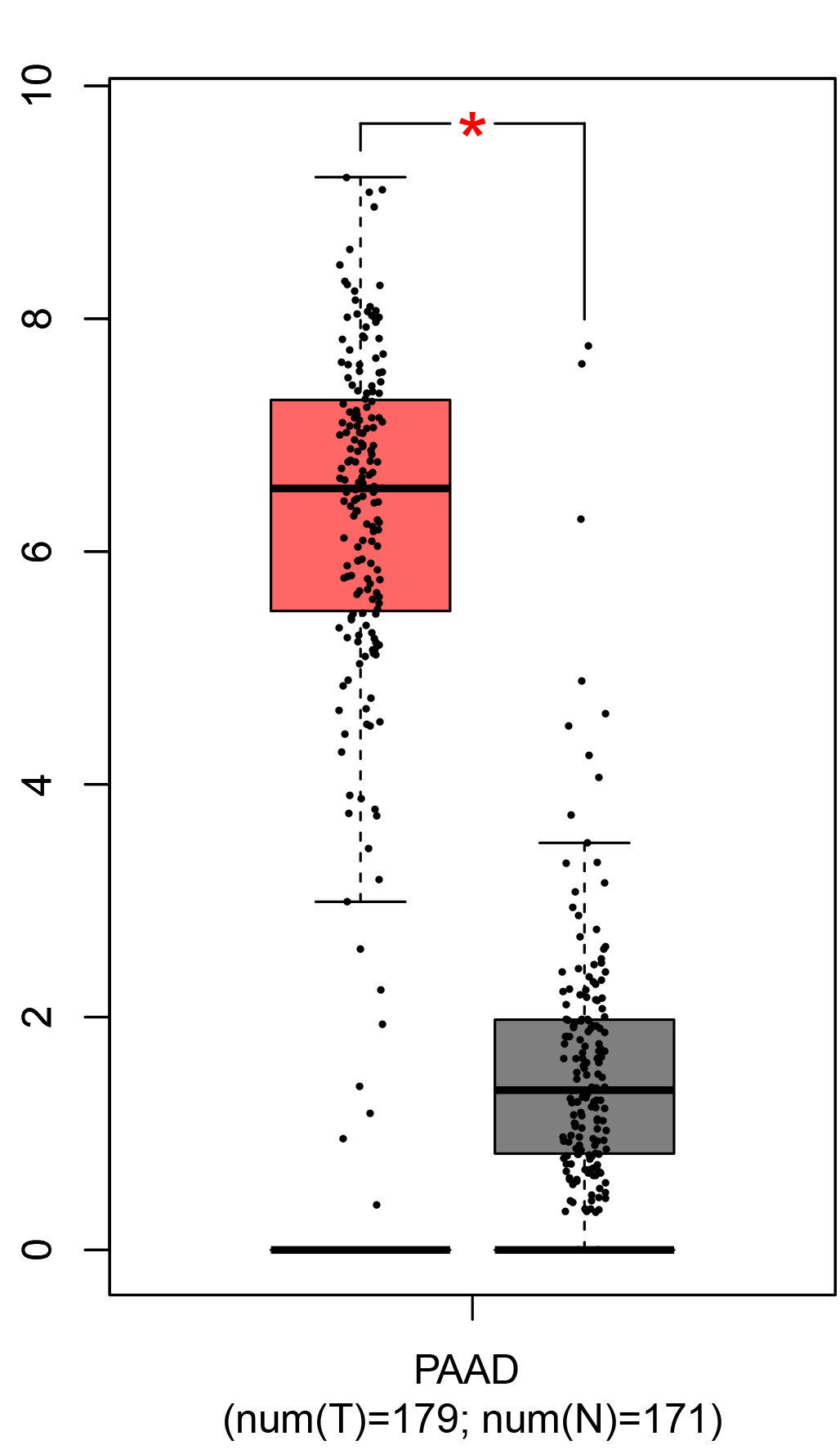

Supplement: Supplemental Information 17 [file peerj-08-10419-s017.png]

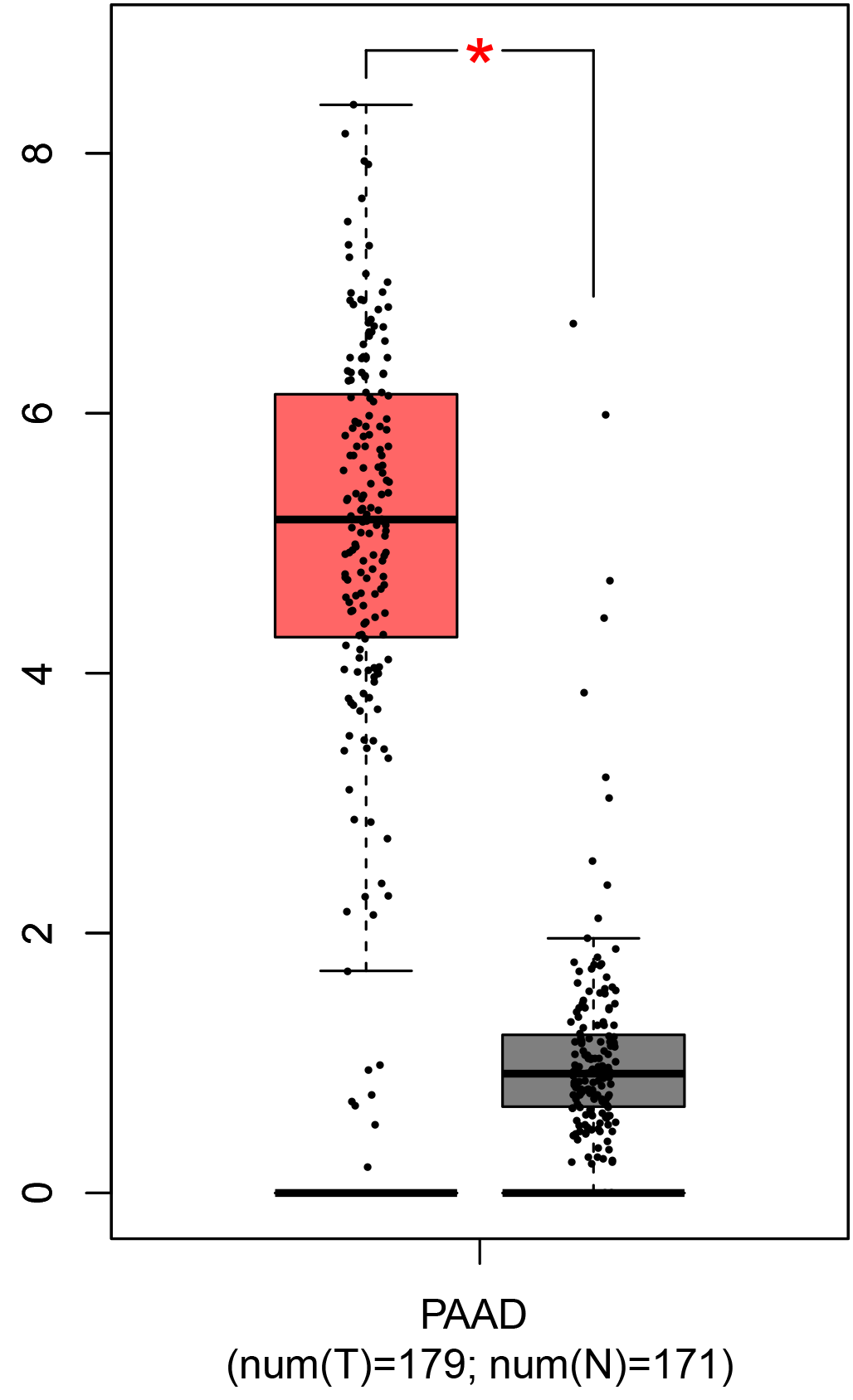

Supplement: Supplemental Information 18 [file peerj-08-10419-s018.png]

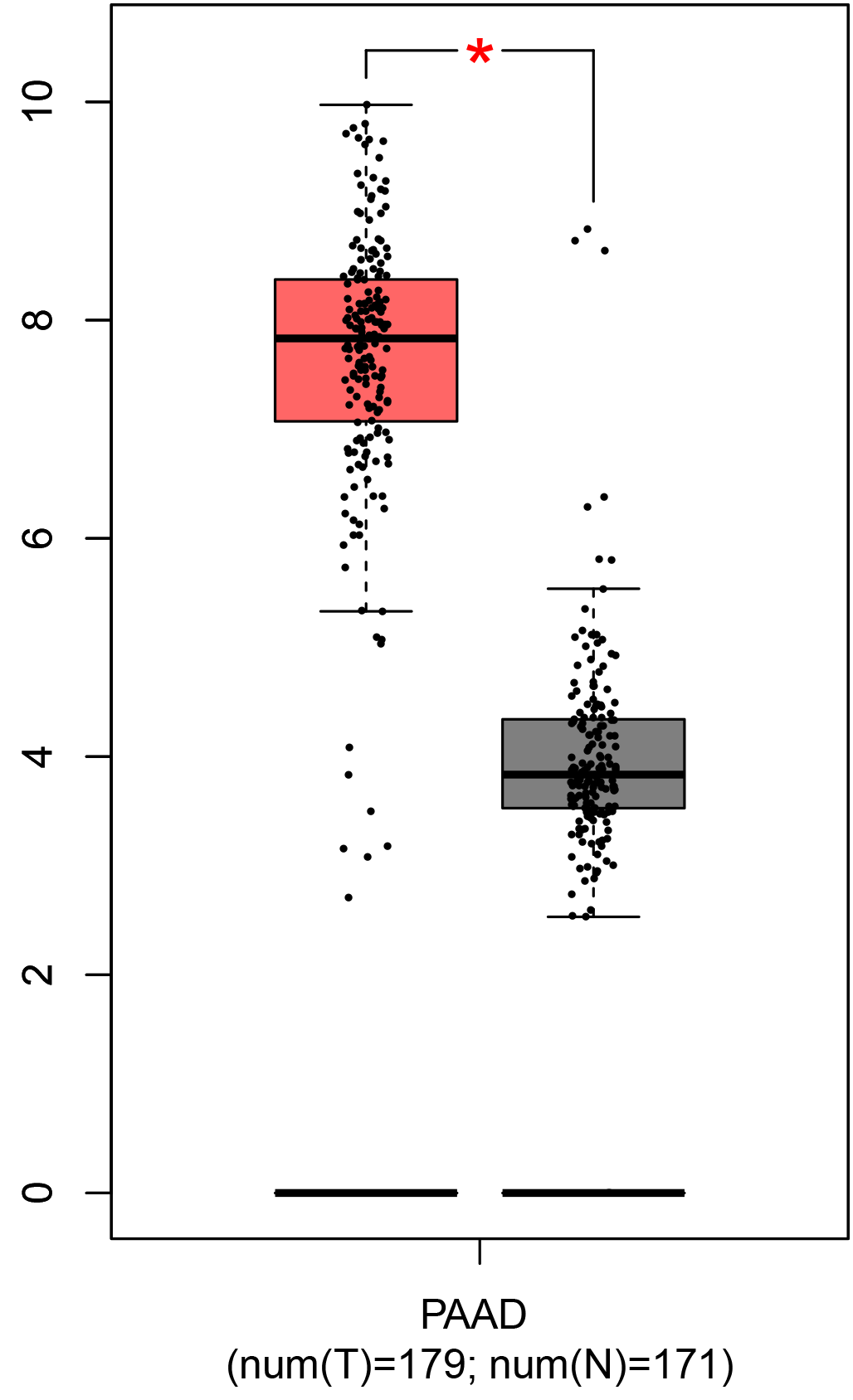

Supplement: Supplemental Information 19 [file peerj-08-10419-s019.png]

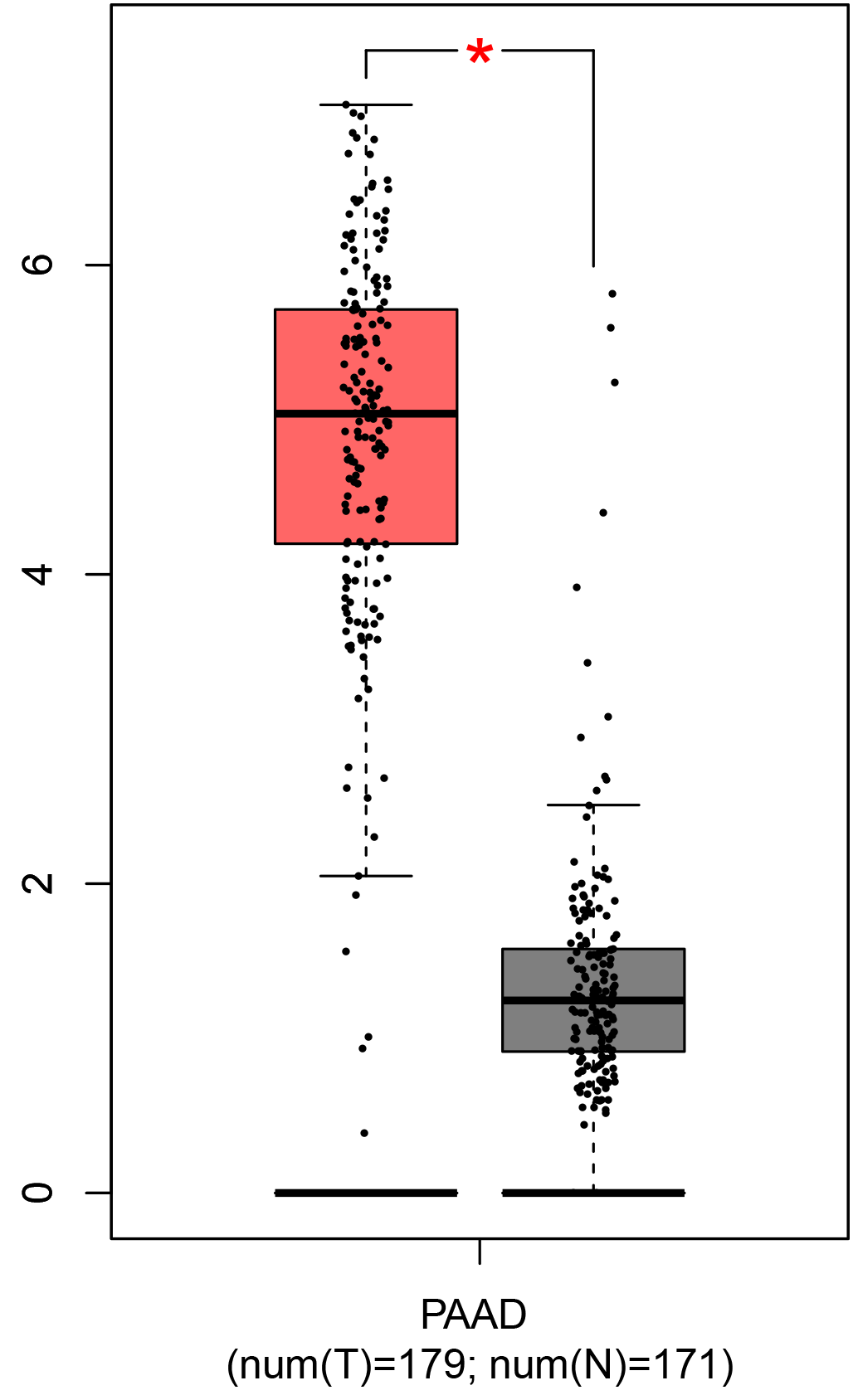

Supplement: Supplemental Information 20 [file peerj-08-10419-s020.png]

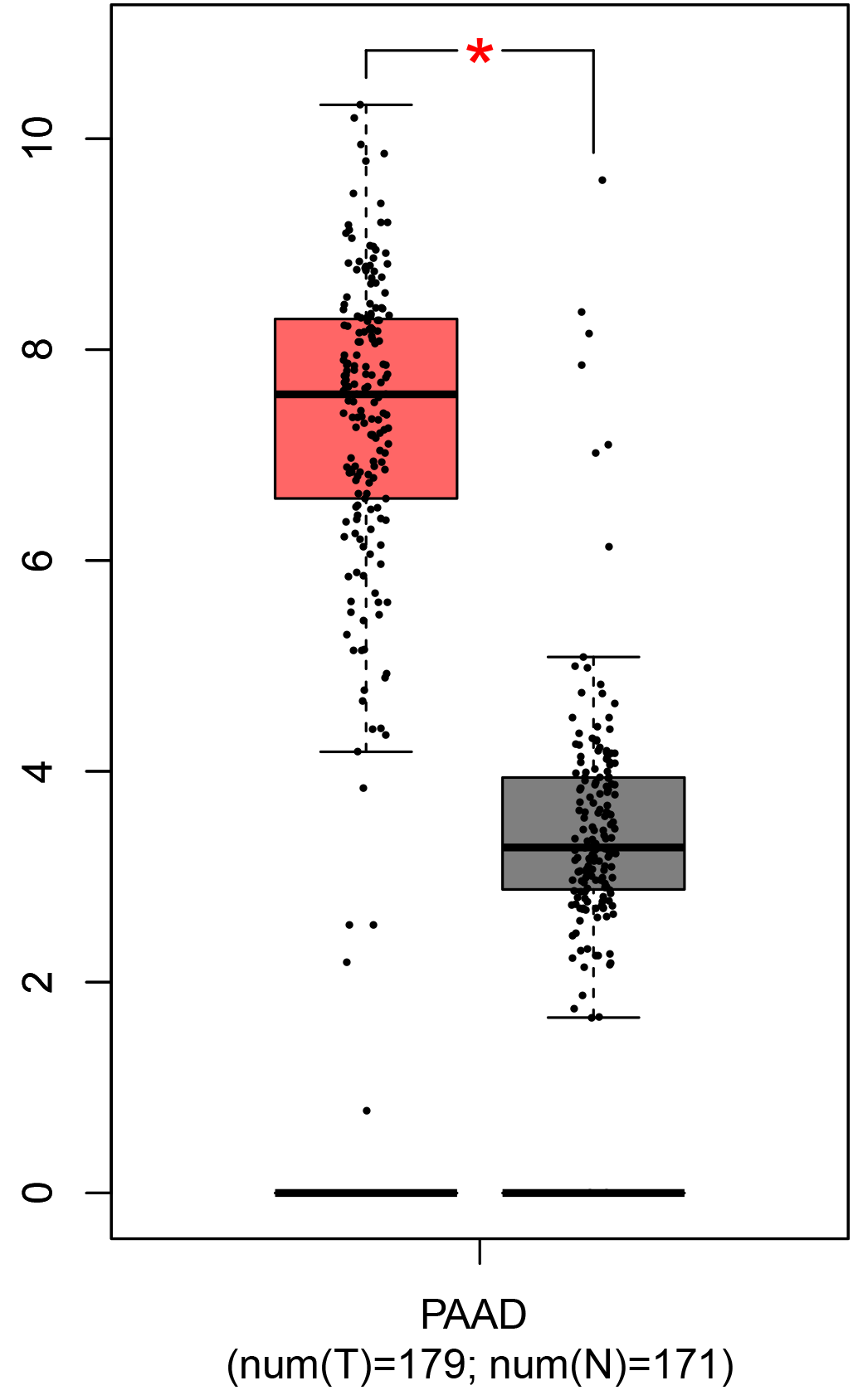

Supplement: Supplemental Information 21 [file peerj-08-10419-s021.png]

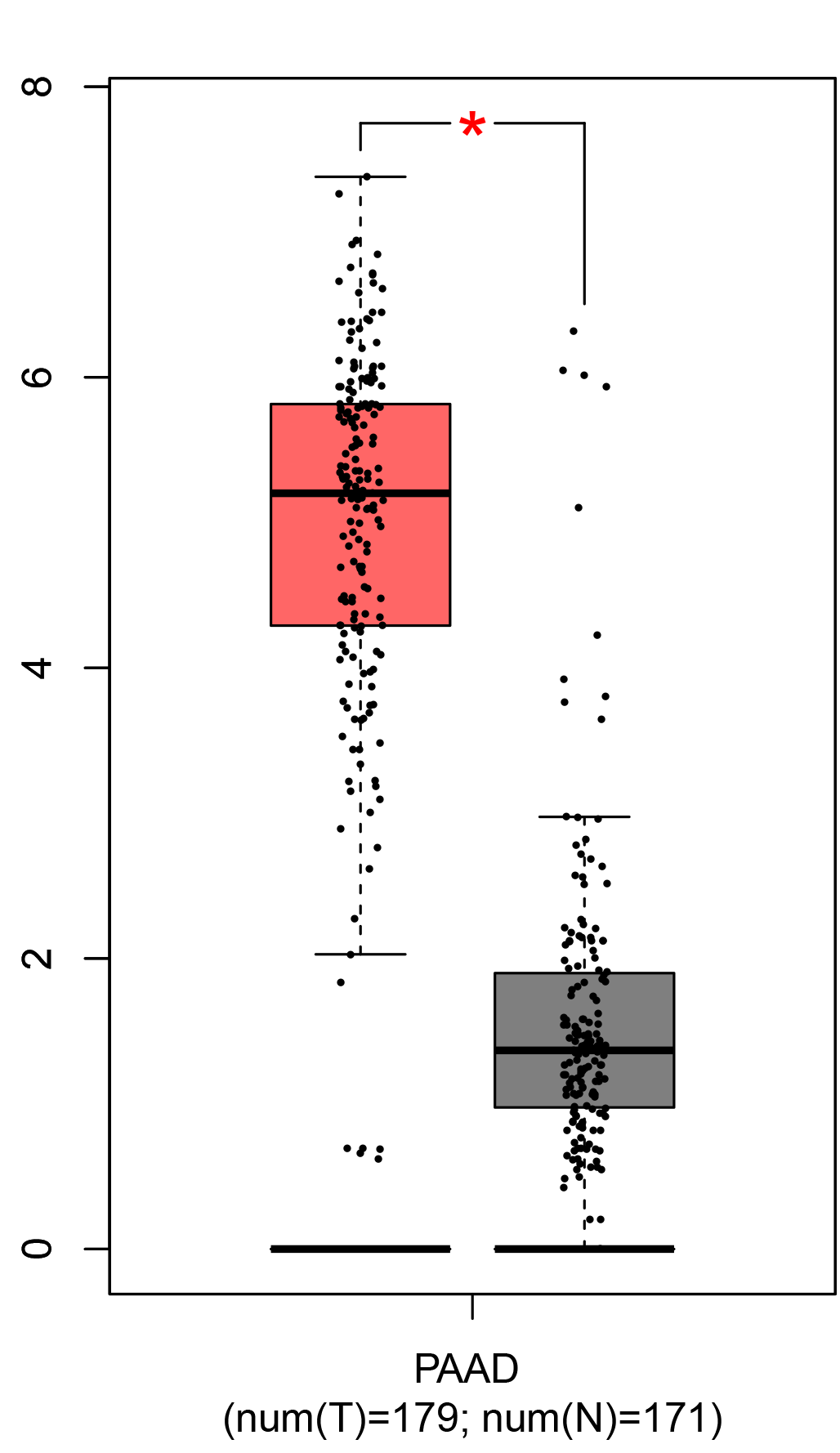

Supplement: Supplemental Information 22 [file peerj-08-10419-s022.png]

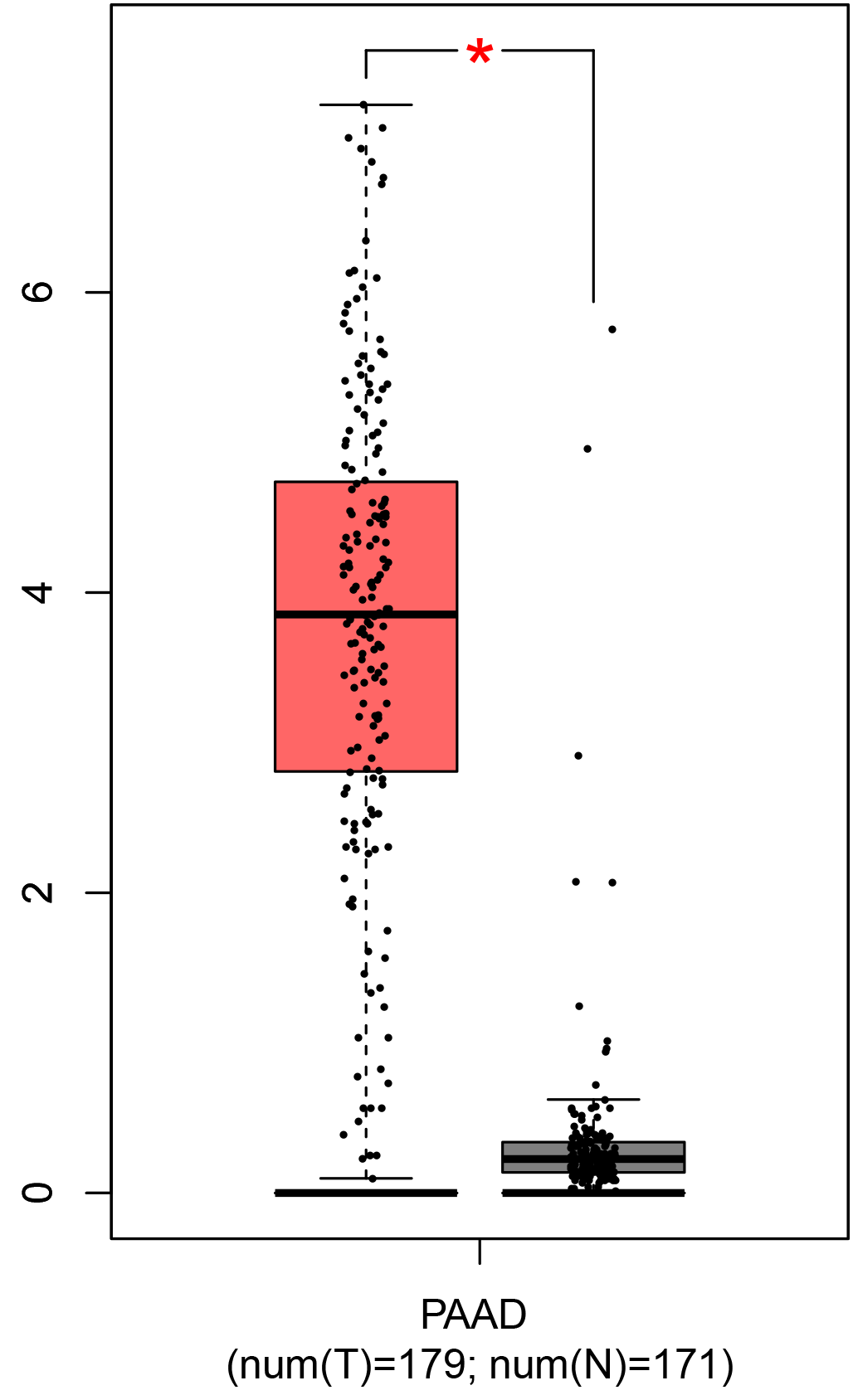

Supplement: Supplemental Information 23 [file peerj-08-10419-s023.png]

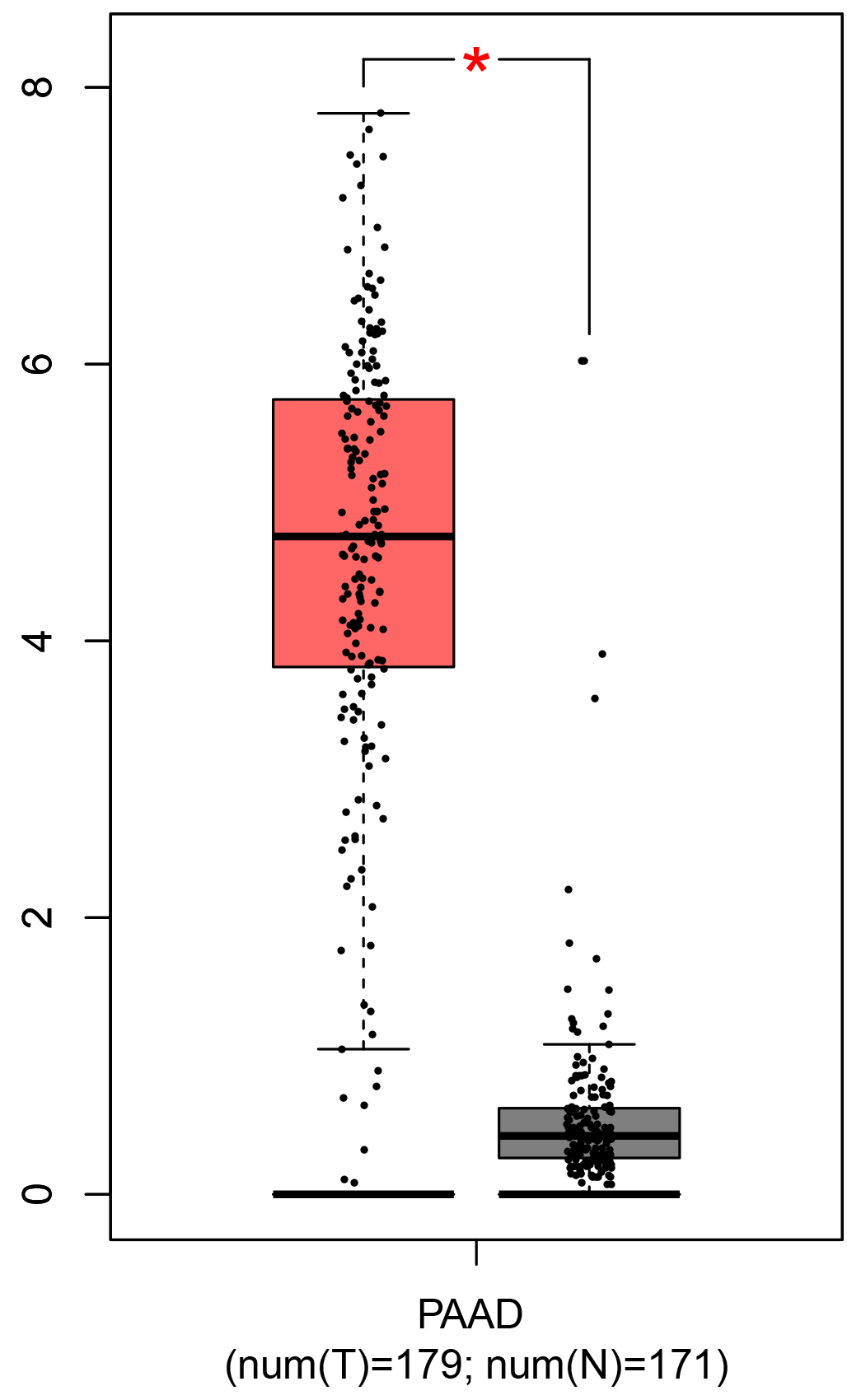

Supplement: Supplemental Information 24 [file peerj-08-10419-s024.png]

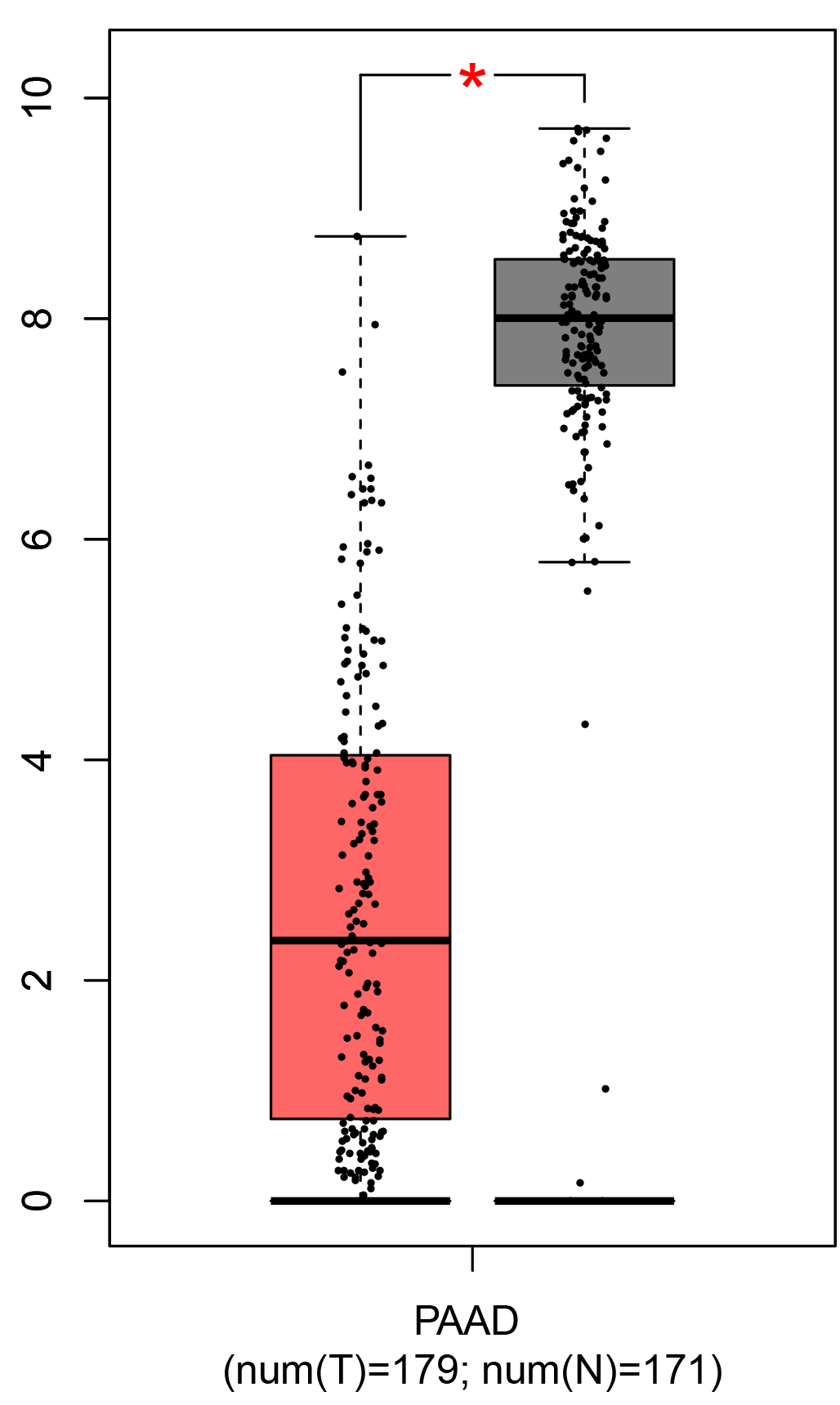

Supplement: Supplemental Information 25 [file peerj-08-10419-s025.png]

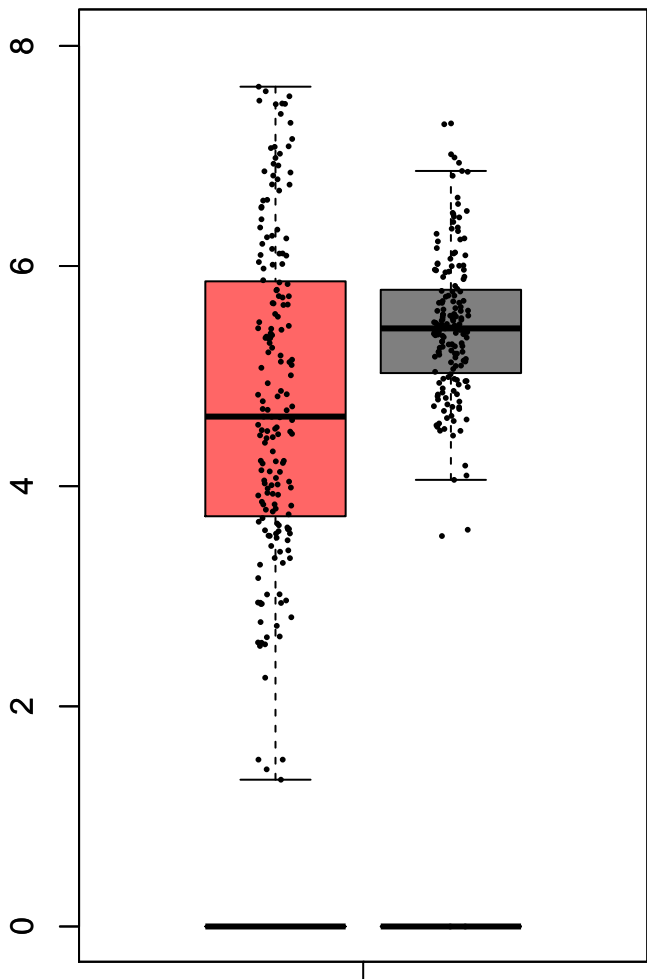

PAAD  
(num(T)=179; num(N)=171)

Supplement: Supplemental Information 26 [file peerj-08-10419-s026.pdf]

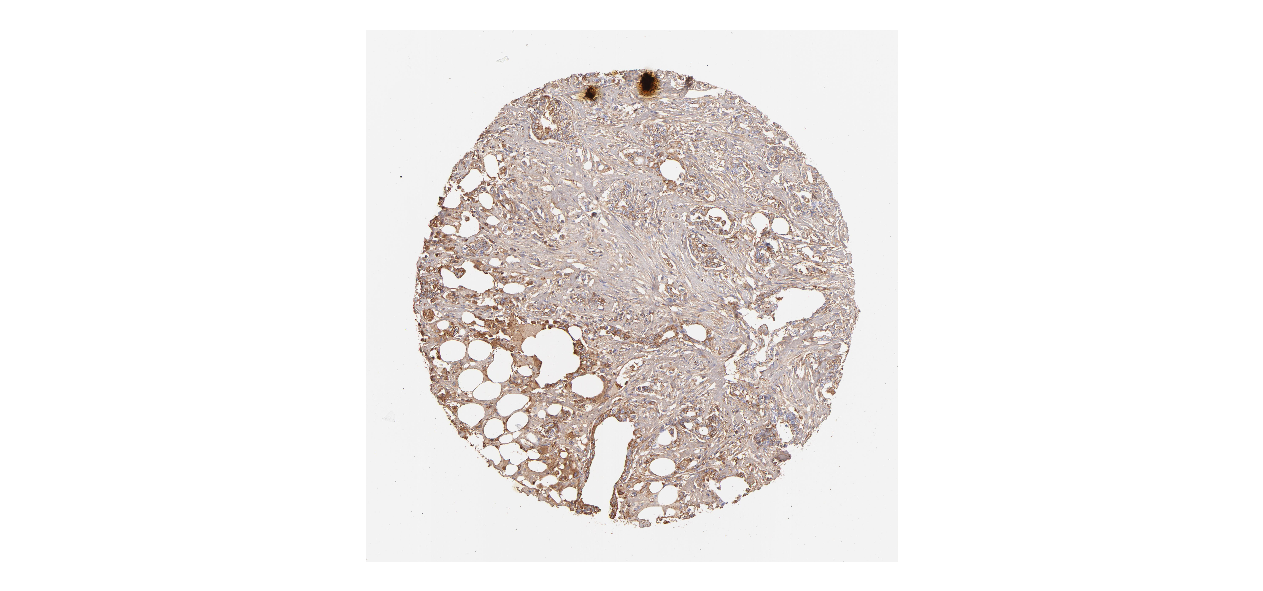

Supplement: Supplemental Information 27 [file peerj-08-10419-s027.png]

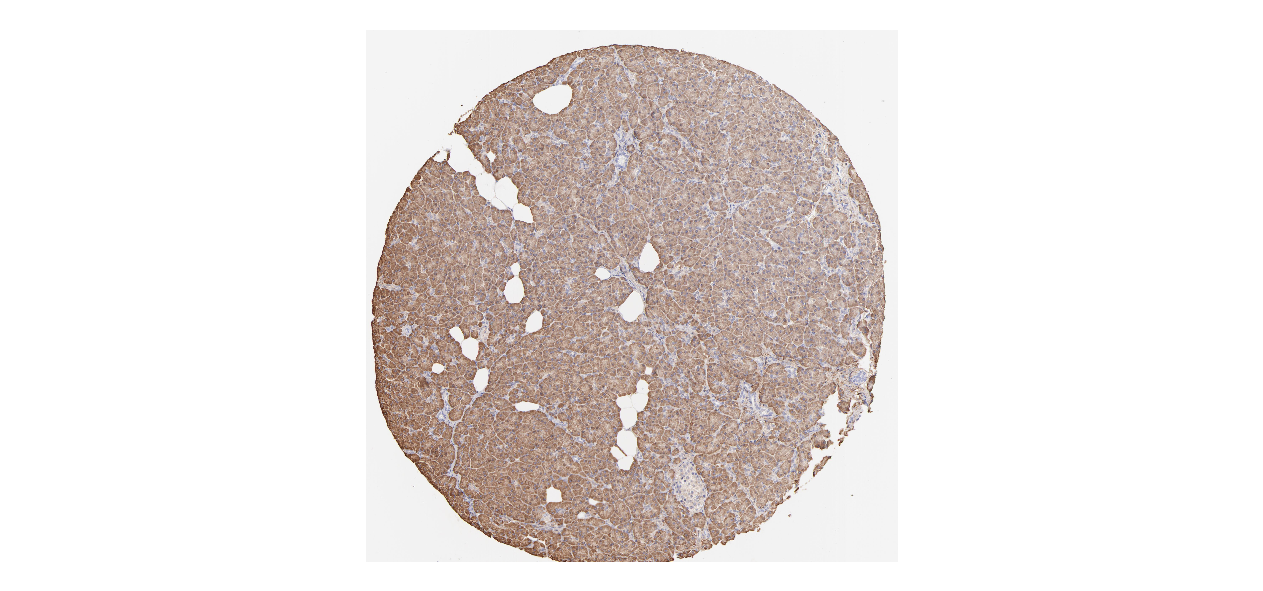

Supplement: Supplemental Information 28 [file peerj-08-10419-s028.png]

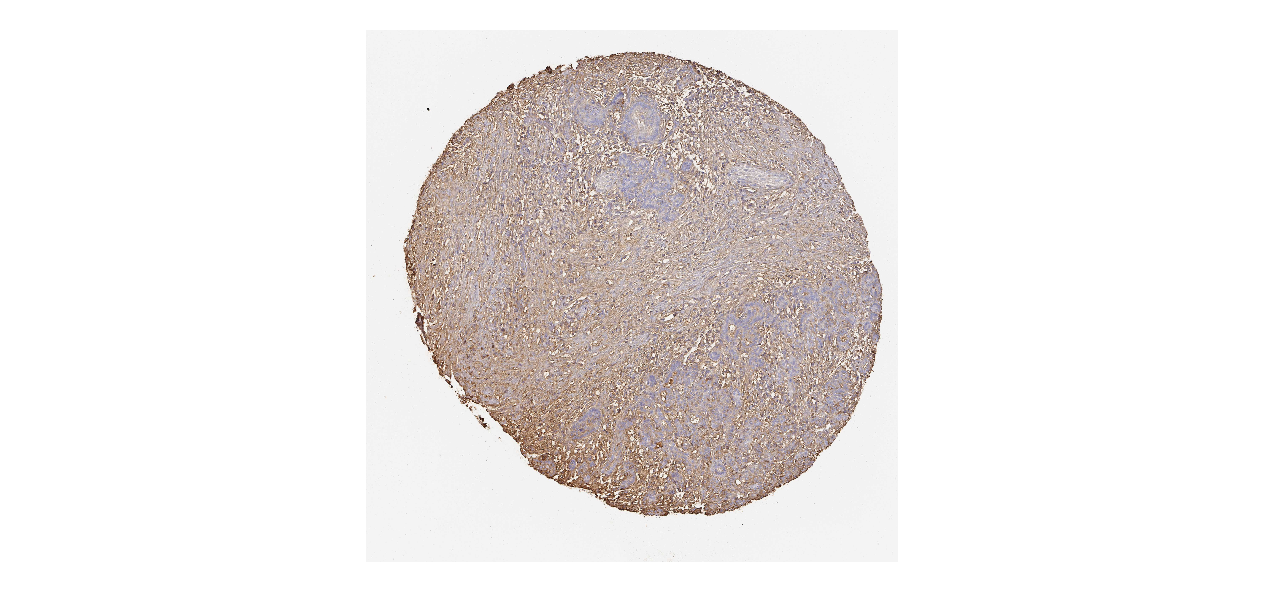

Supplement: Supplemental Information 29 [file peerj-08-10419-s029.png]

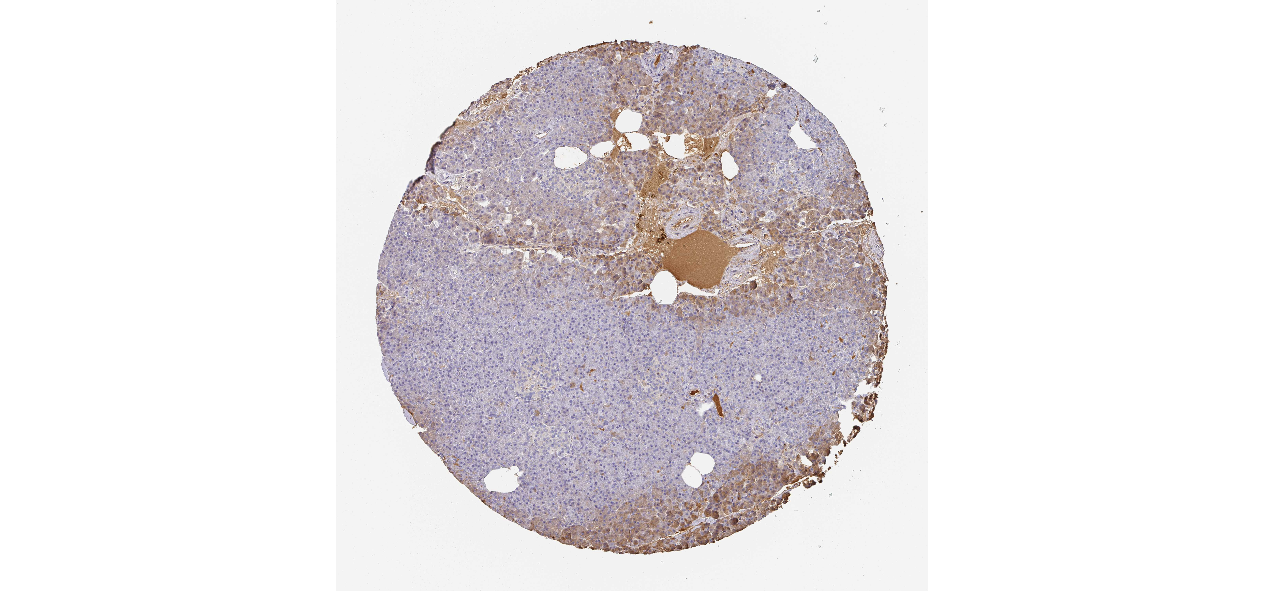

Supplement: Supplemental Information 30 [file peerj-08-10419-s030.png]

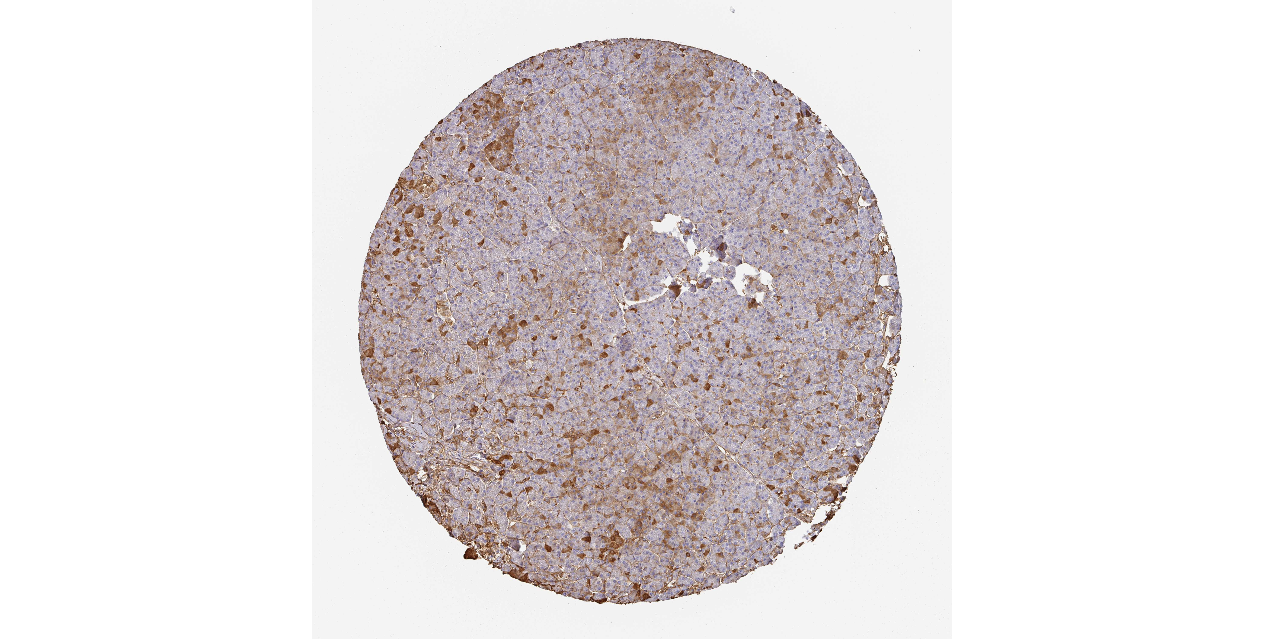

Supplement: Supplemental Information 31 [file peerj-08-10419-s031.png]

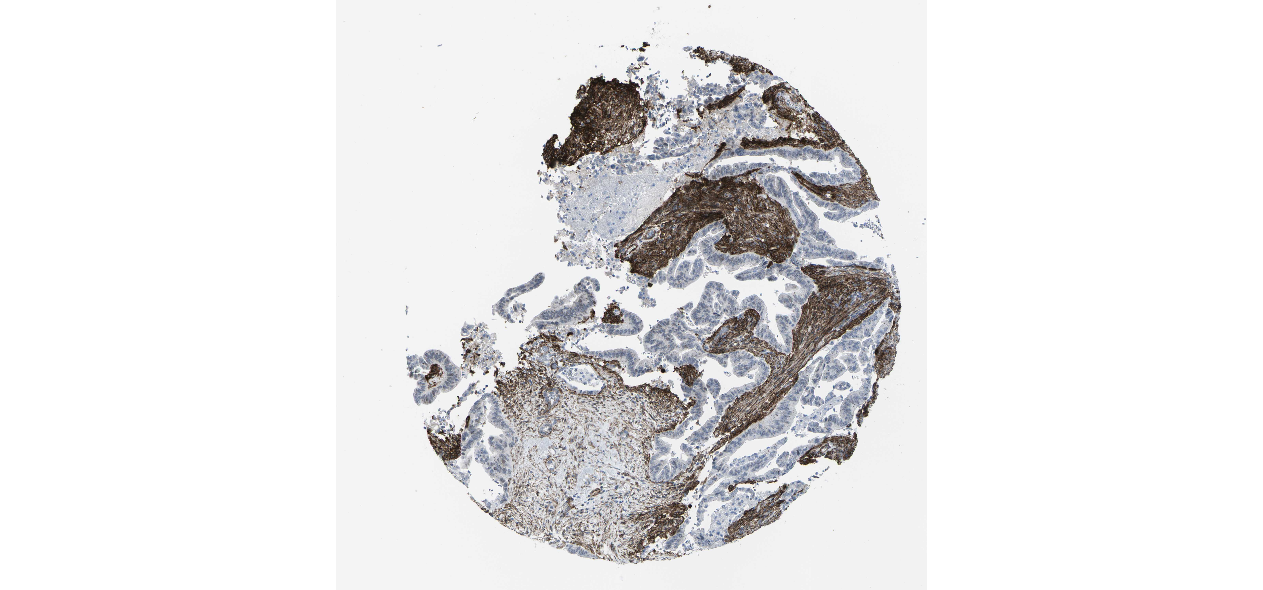

Supplement: Supplemental Information 32 [file peerj-08-10419-s032.png]

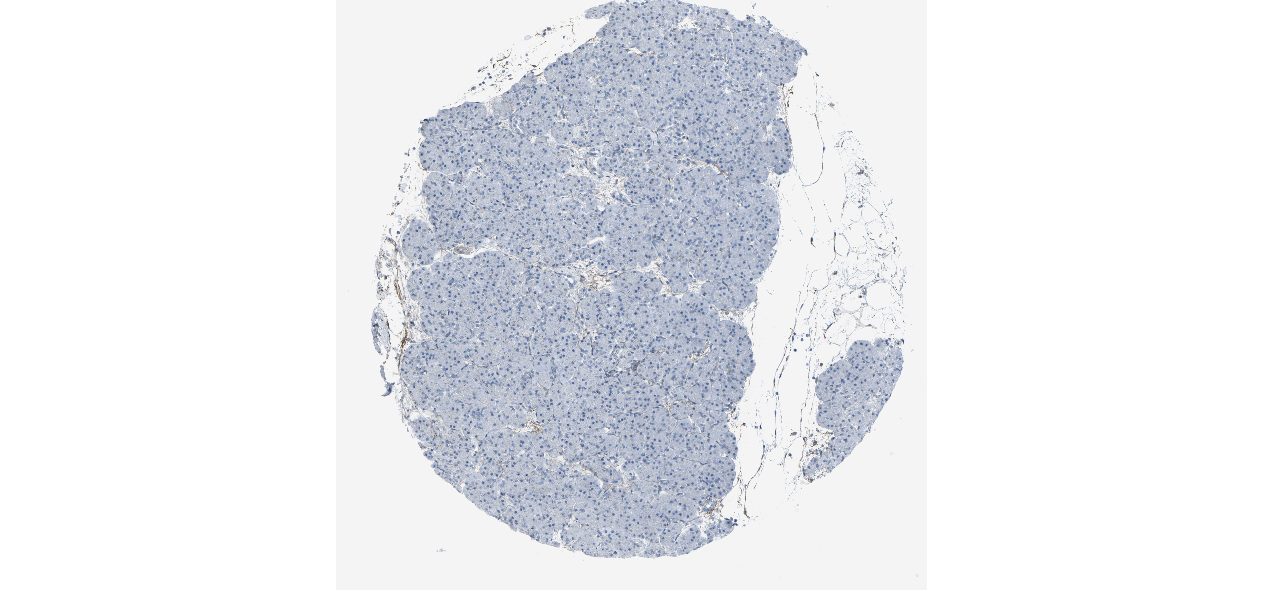

Supplement: Supplemental Information 33 [file peerj-08-10419-s033.png]

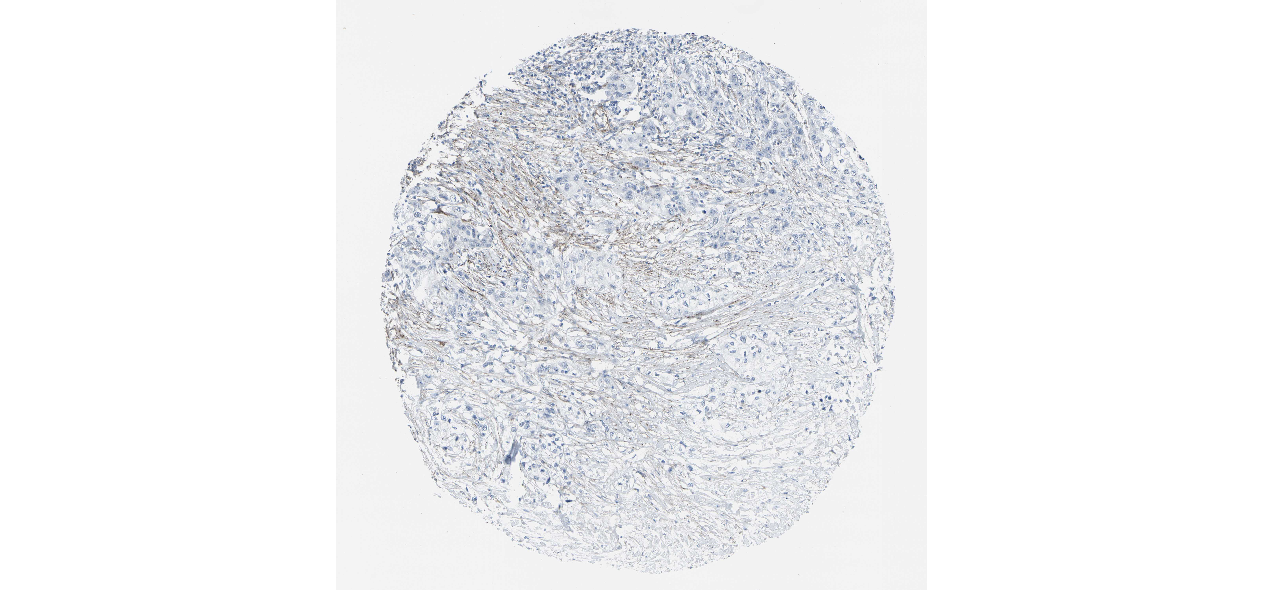

Supplement: Supplemental Information 34 [file peerj-08-10419-s034.png]

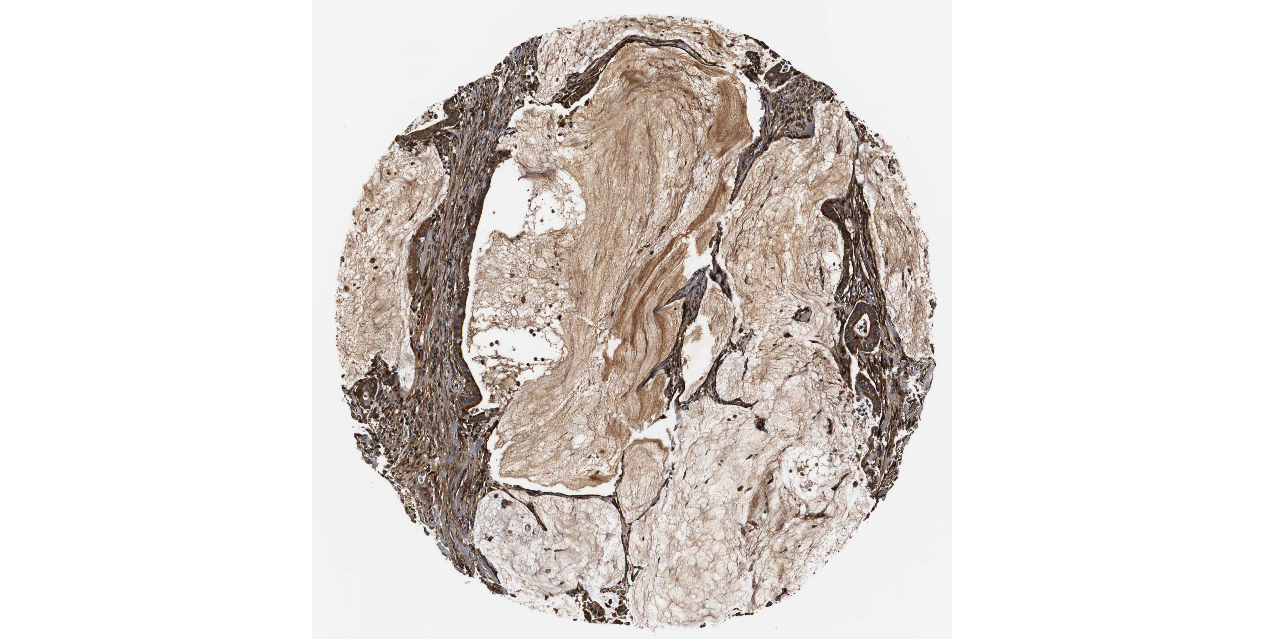

Supplement: Supplemental Information 35 [file peerj-08-10419-s035.png]

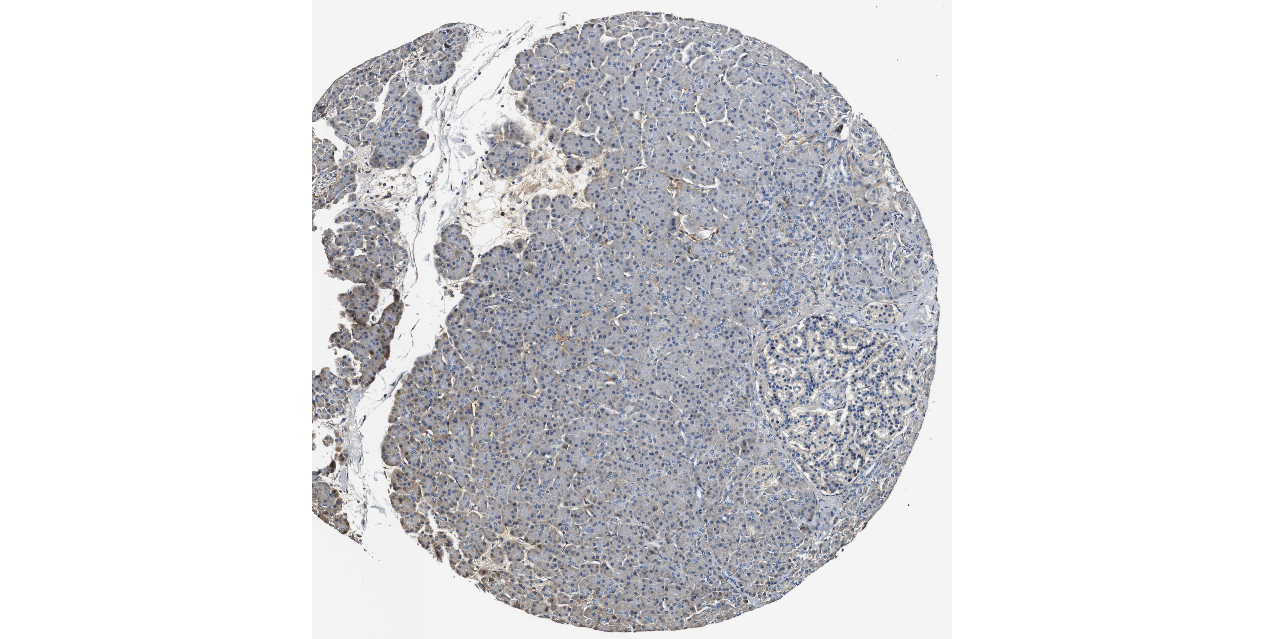

Supplement: Supplemental Information 36 [file peerj-08-10419-s036.png]

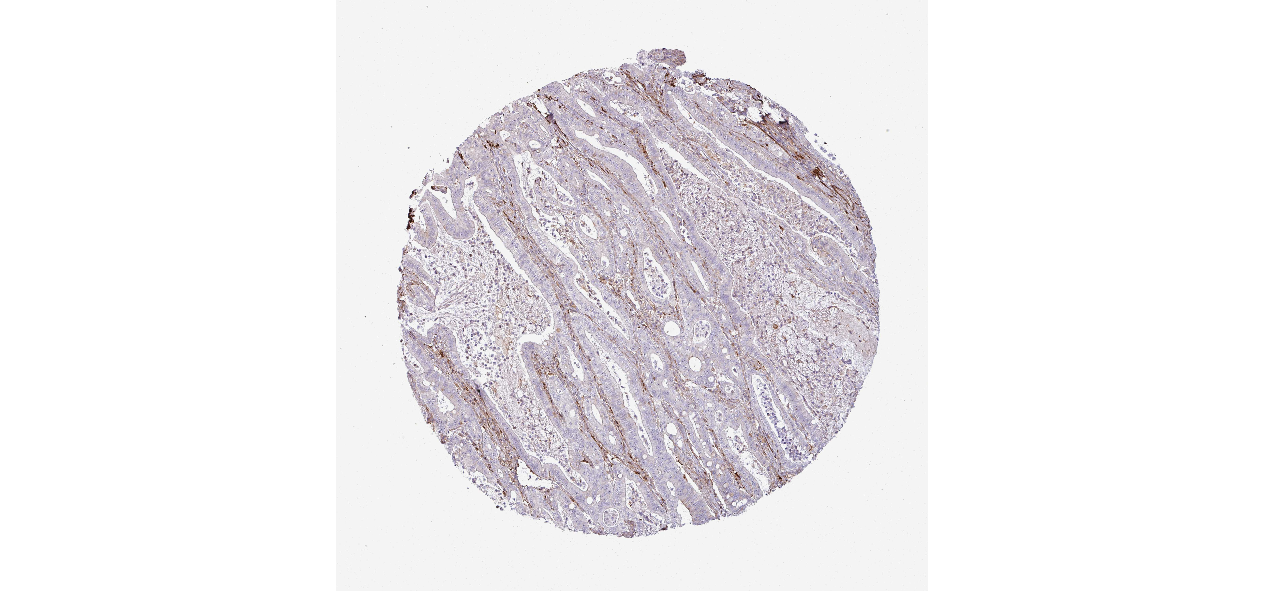

Supplement: Supplemental Information 37 [file peerj-08-10419-s037.png]

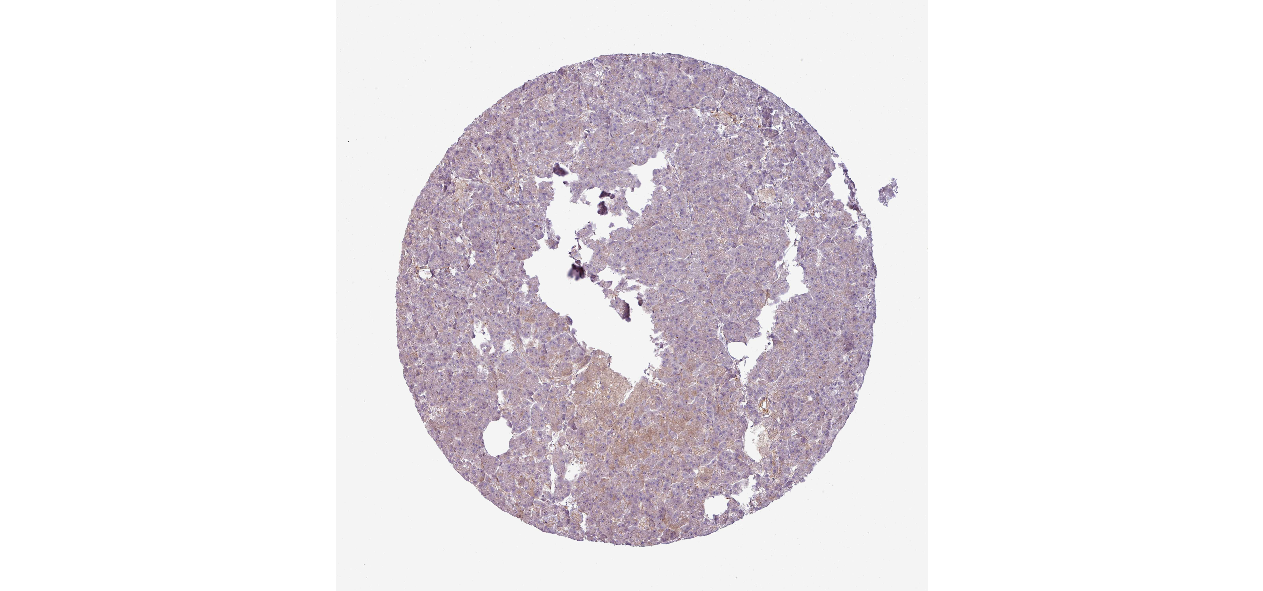

Supplement: Supplemental Information 38 [file peerj-08-10419-s038.png]

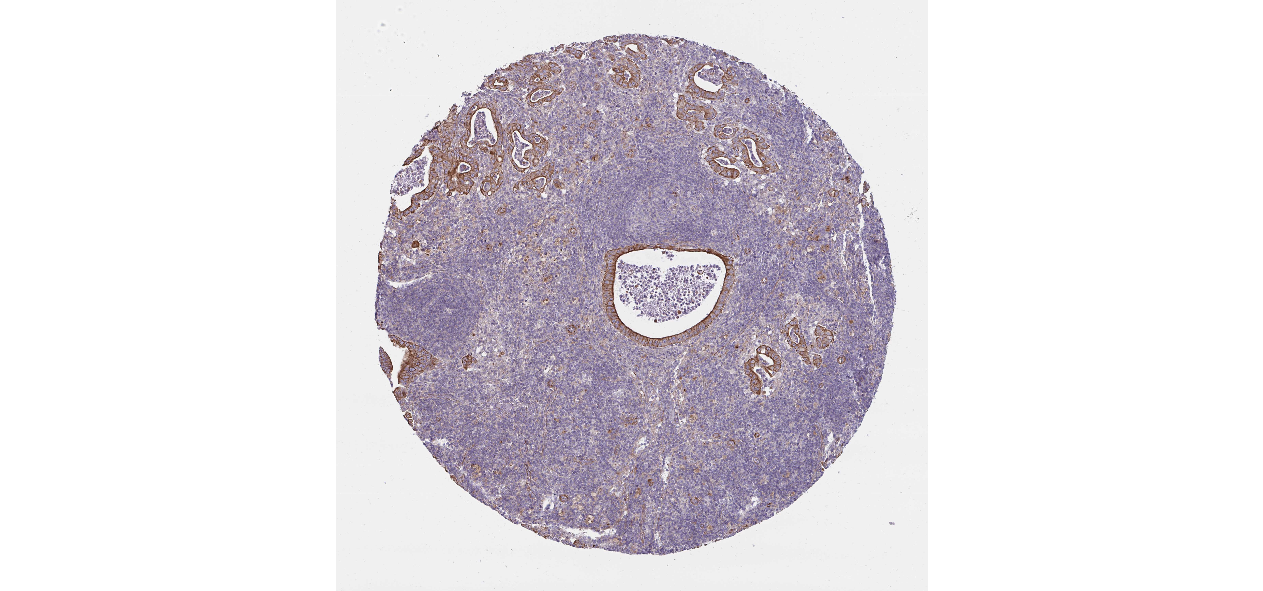

Supplement: Supplemental Information 39 [file peerj-08-10419-s039.png]

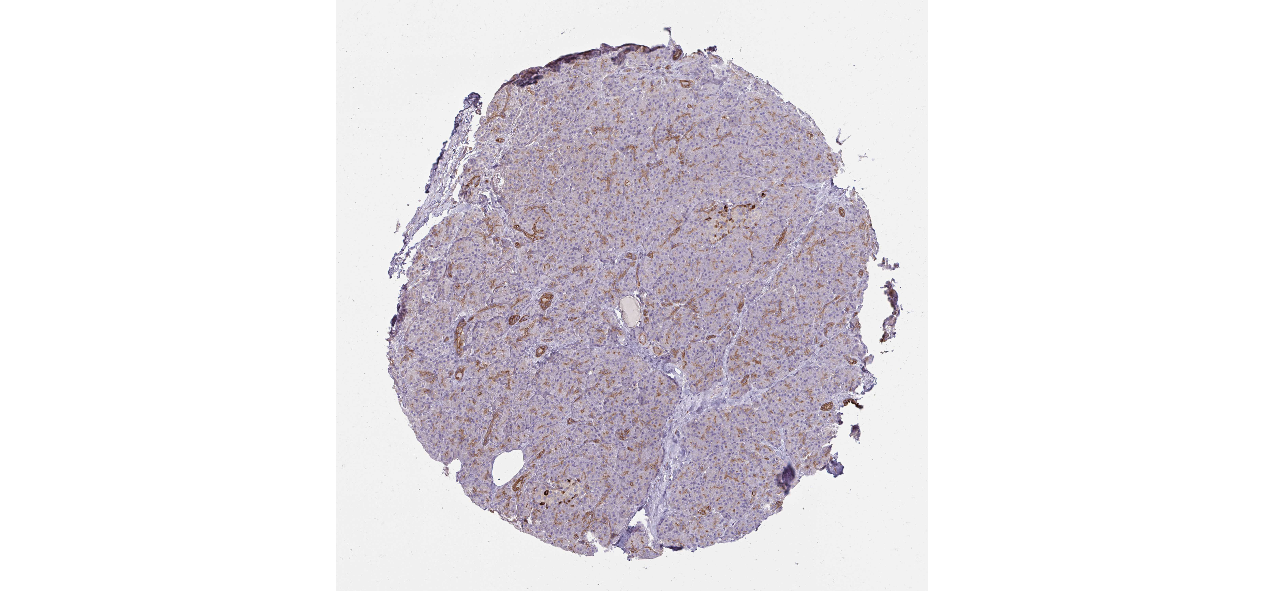

Supplement: Supplemental Information 40 [file peerj-08-10419-s040.png]

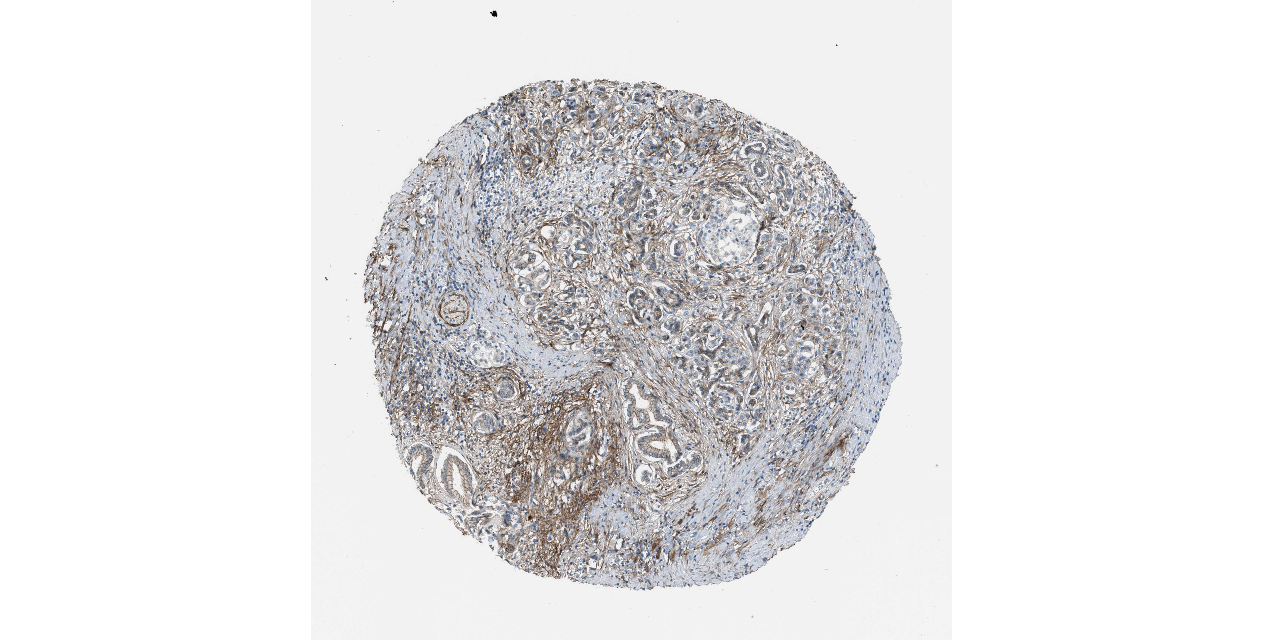

Supplement: Supplemental Information 41 [file peerj-08-10419-s041.png]

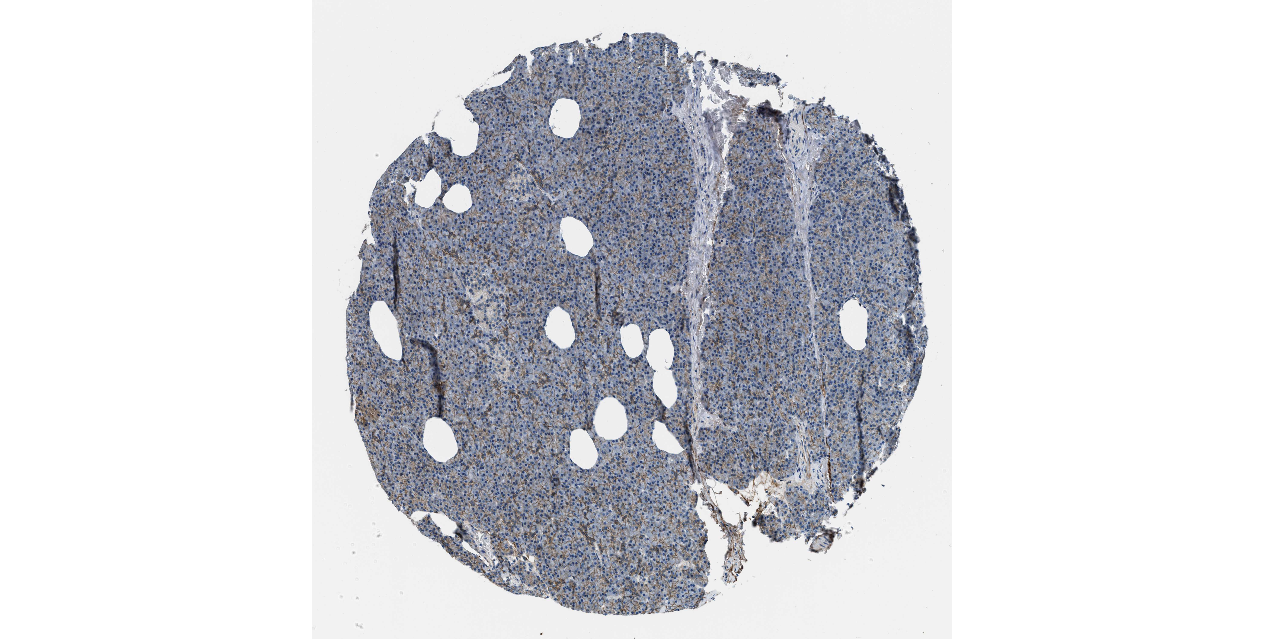

Supplement: Supplemental Information 42 [file peerj-08-10419-s042.png]

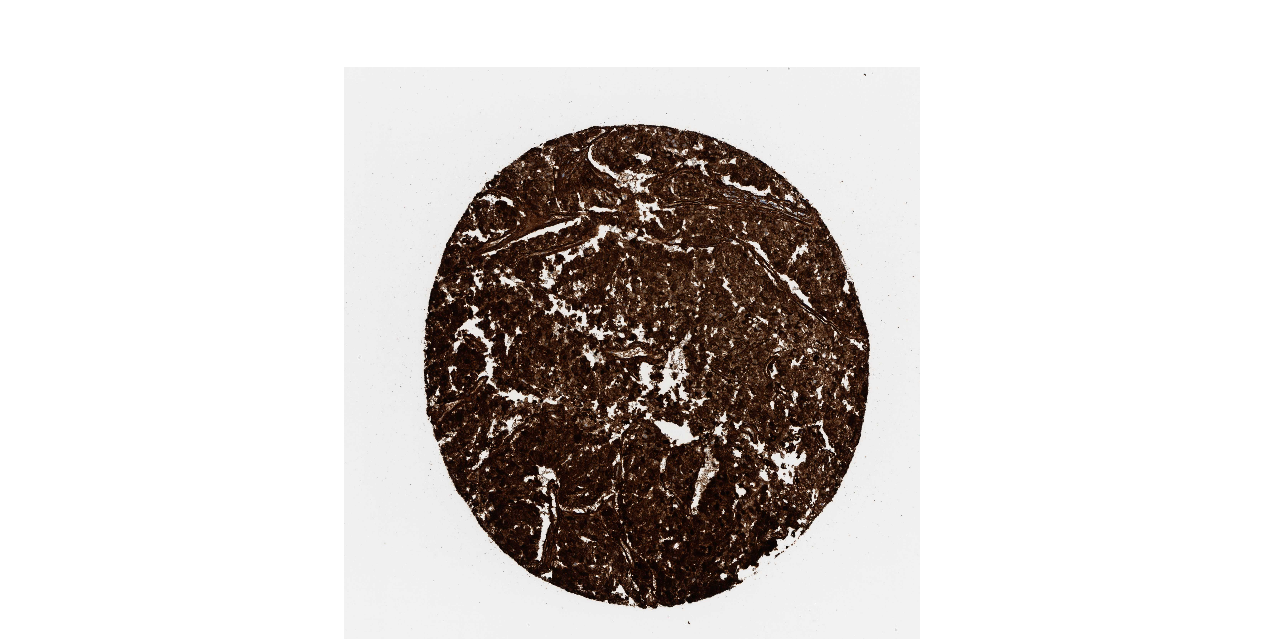

Supplement: Supplemental Information 43 [file peerj-08-10419-s043.png]

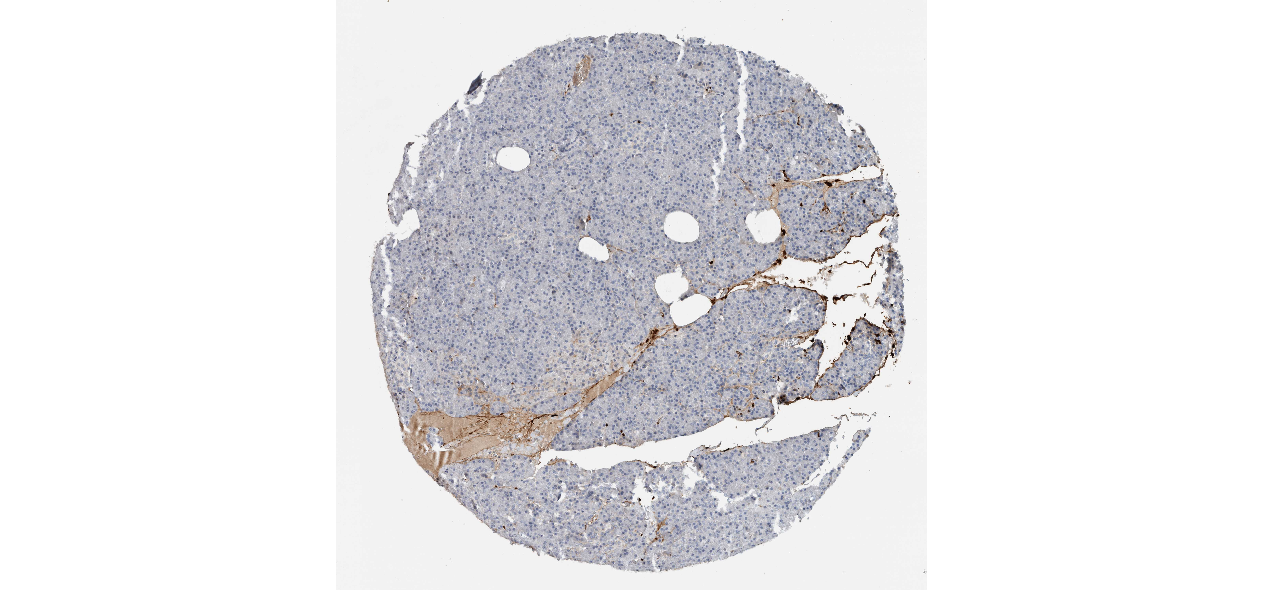

Supplement: Supplemental Information 44 [file peerj-08-10419-s044.png]

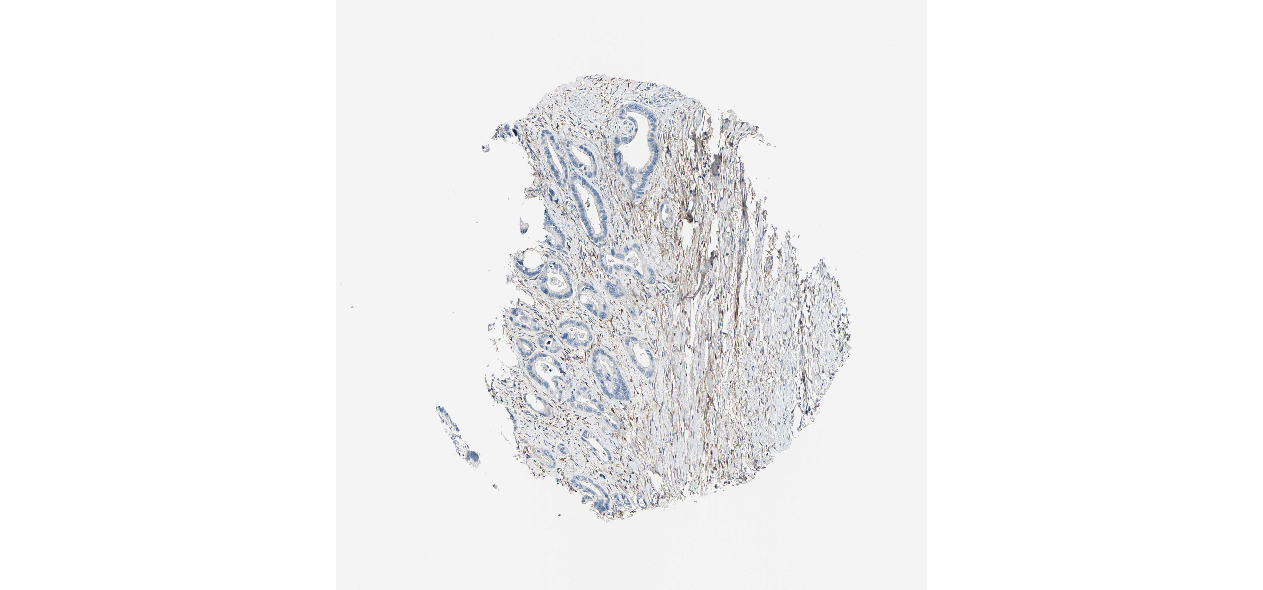

Supplement: Supplemental Information 45 [file peerj-08-10419-s045.png]

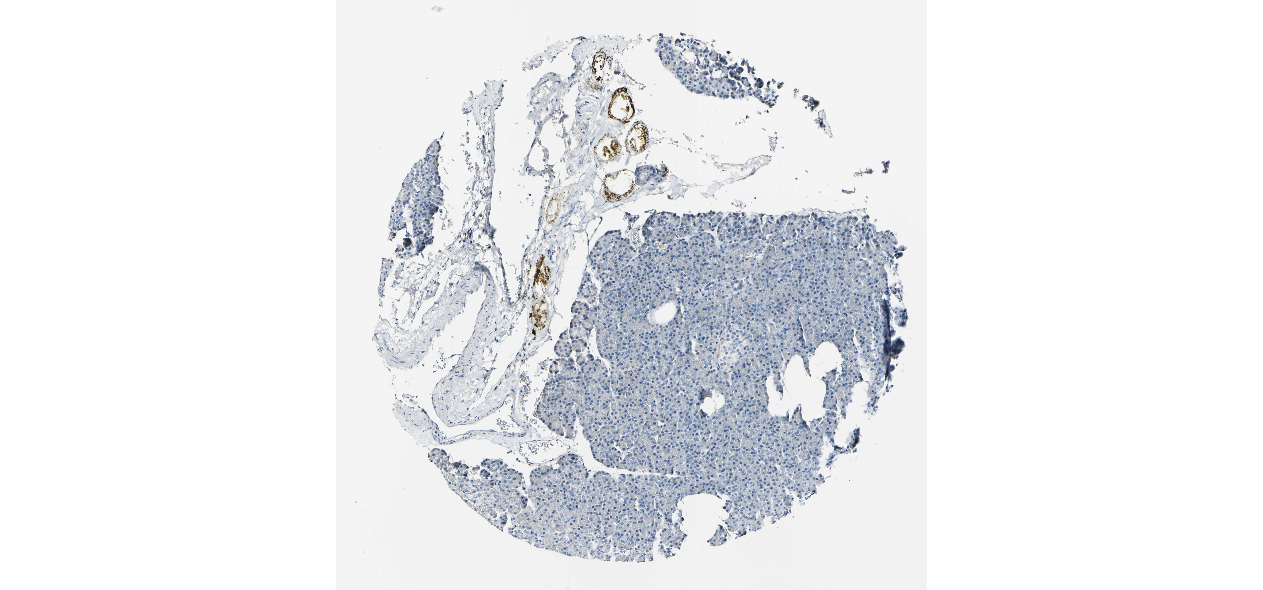

Supplement: Supplemental Information 46 [file peerj-08-10419-s046.png]

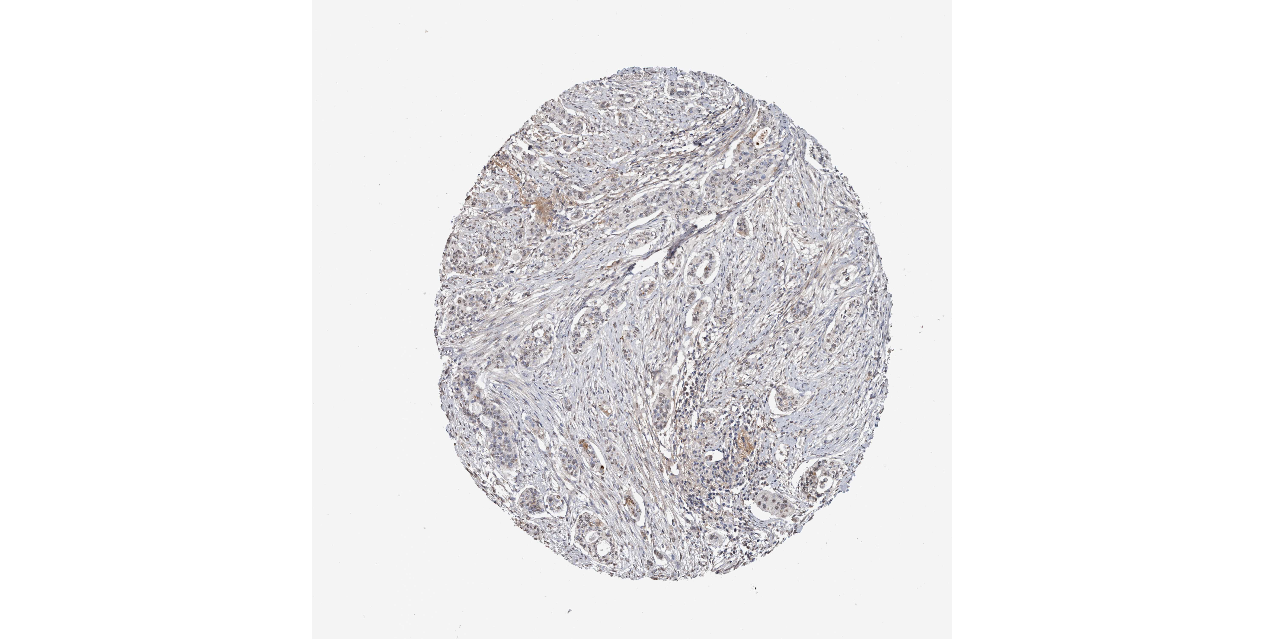

Supplement: Supplemental Information 47 [file peerj-08-10419-s047.png]

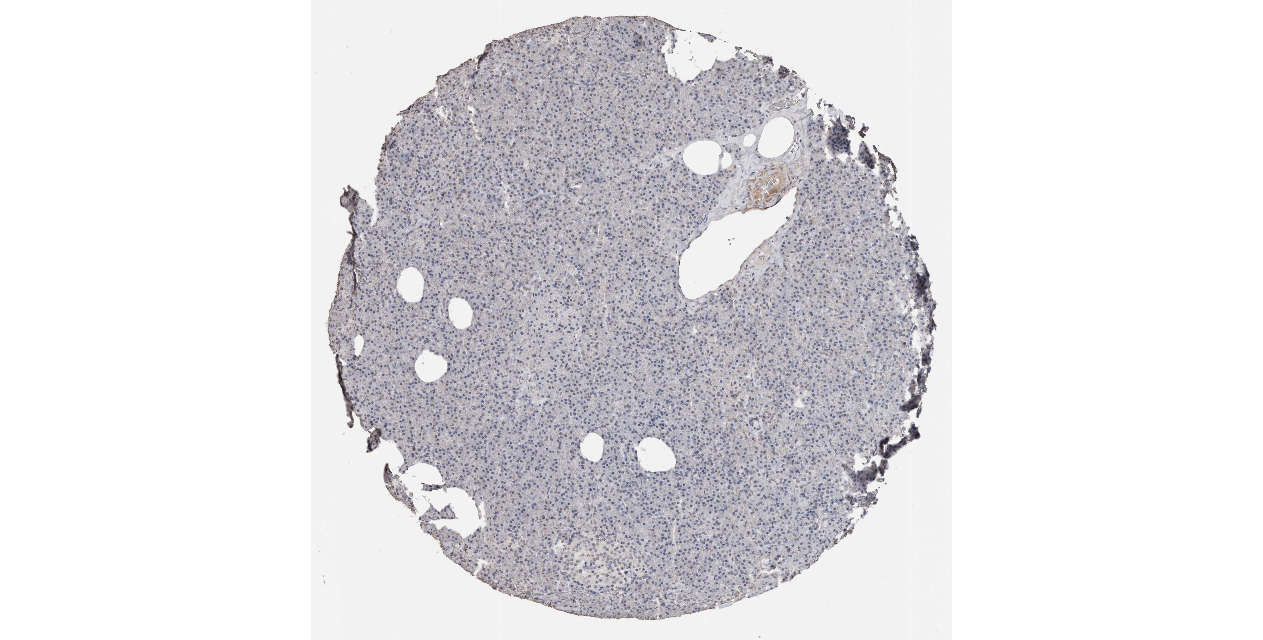

Supplement: Supplemental Information 48 [file peerj-08-10419-s048.png]

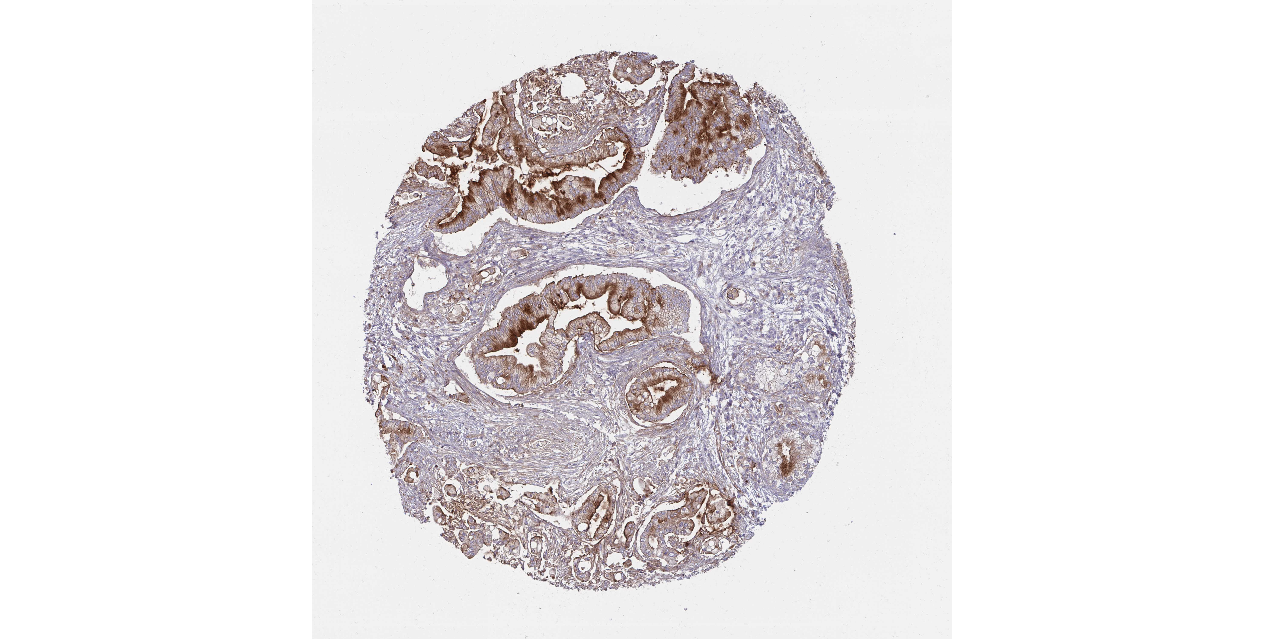

Supplement: Supplemental Information 49 [file peerj-08-10419-s049.png]

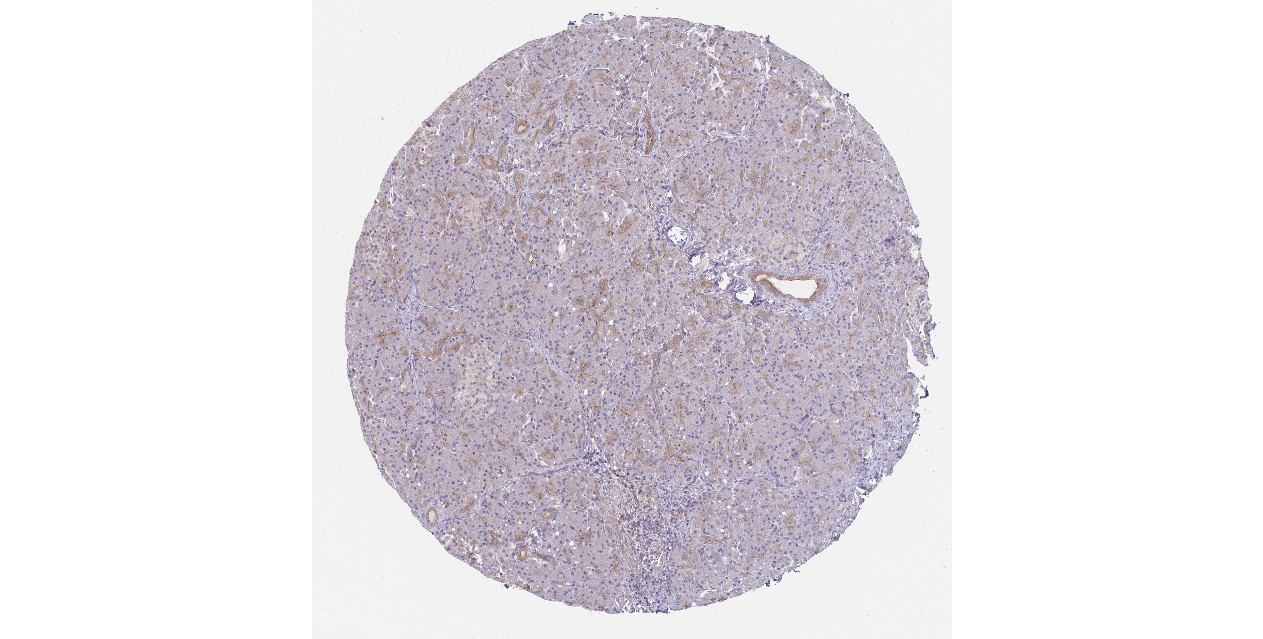

Supplement: Supplemental Information 50 [file peerj-08-10419-s050.png]

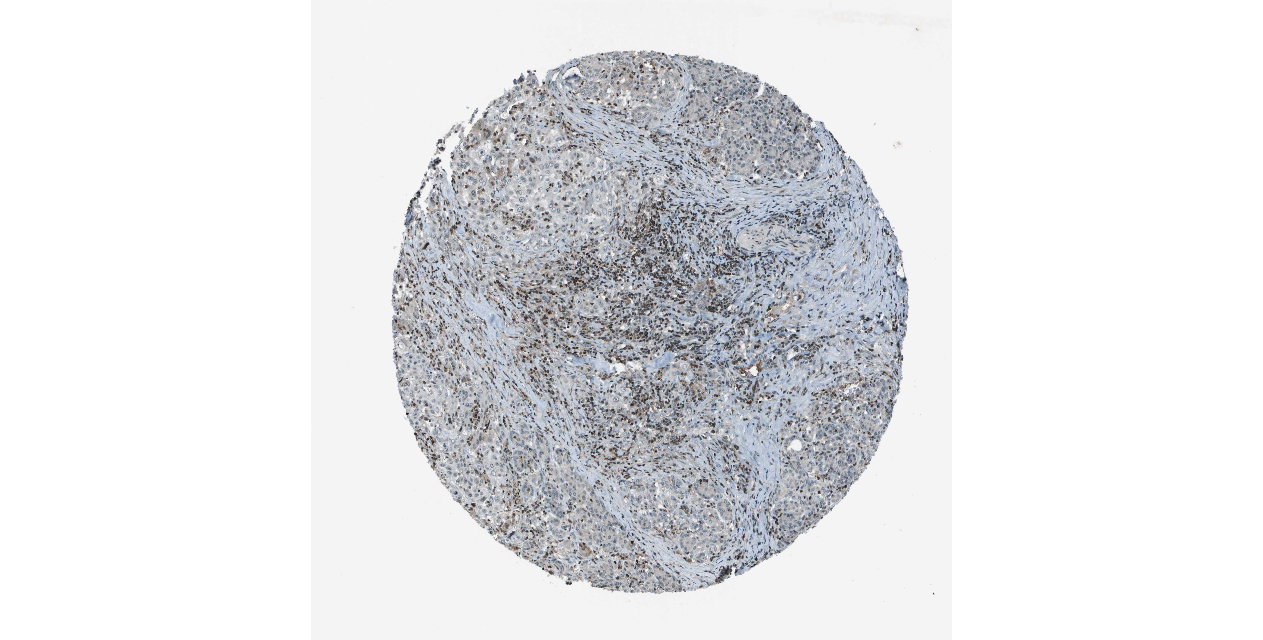

Supplement: Supplemental Information 51 [file peerj-08-10419-s051.png]

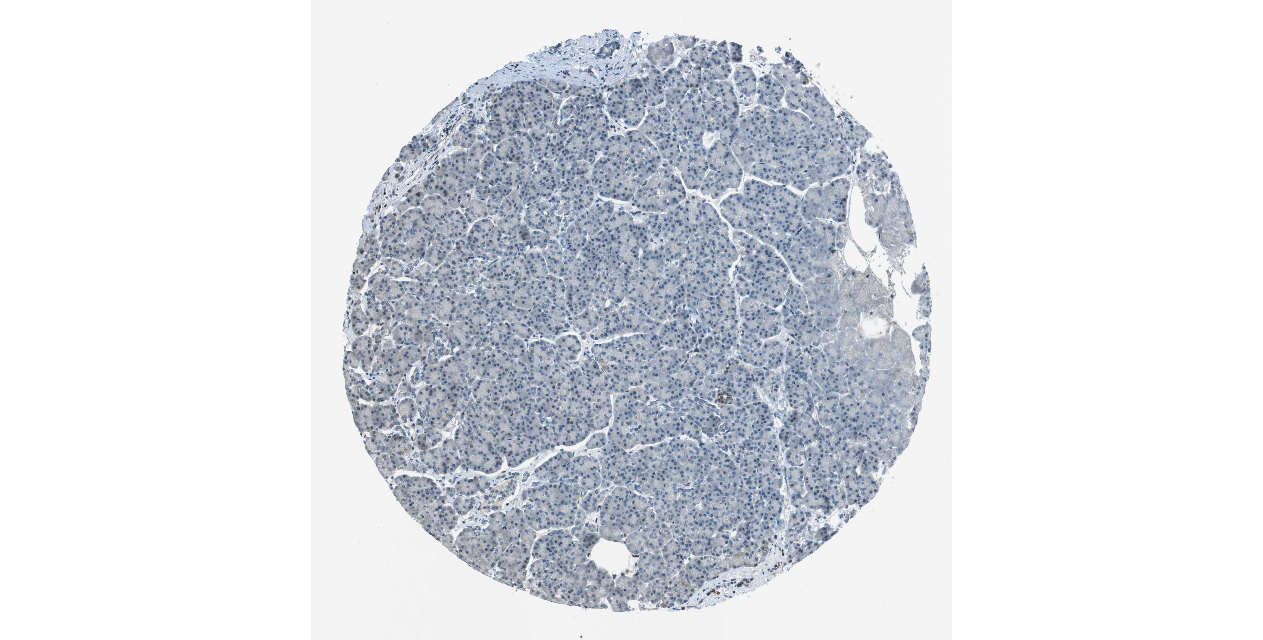

Supplement: Supplemental Information 52 [file peerj-08-10419-s052.png]

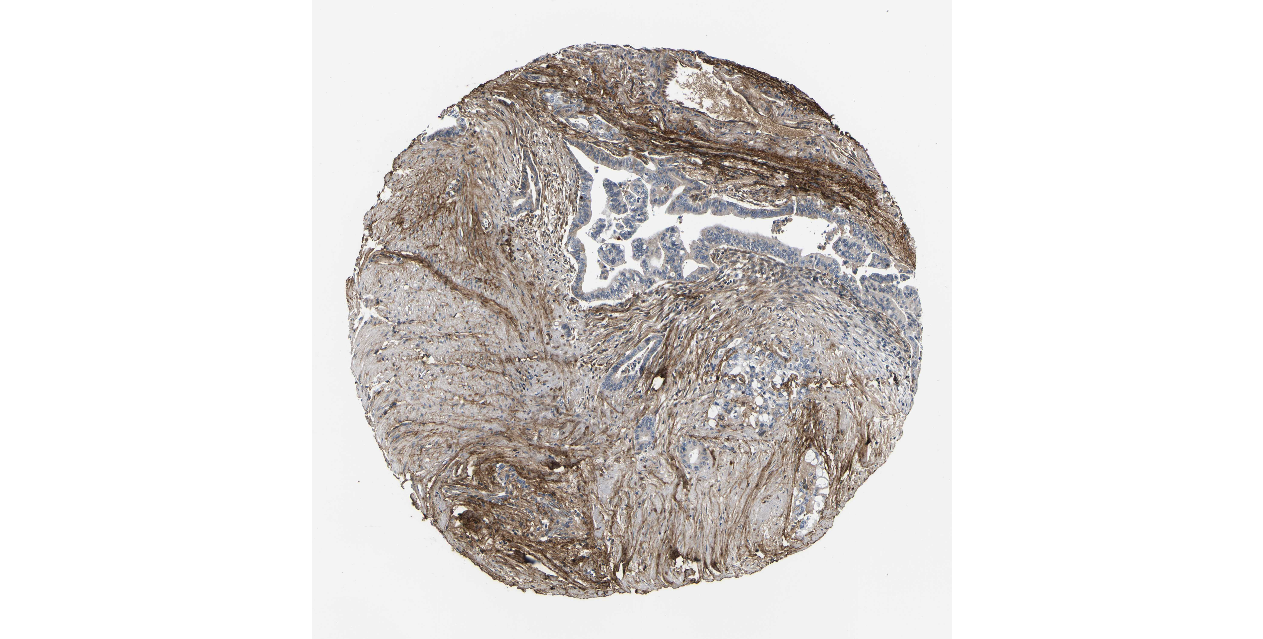

Supplement: Supplemental Information 53 [file peerj-08-10419-s053.png]

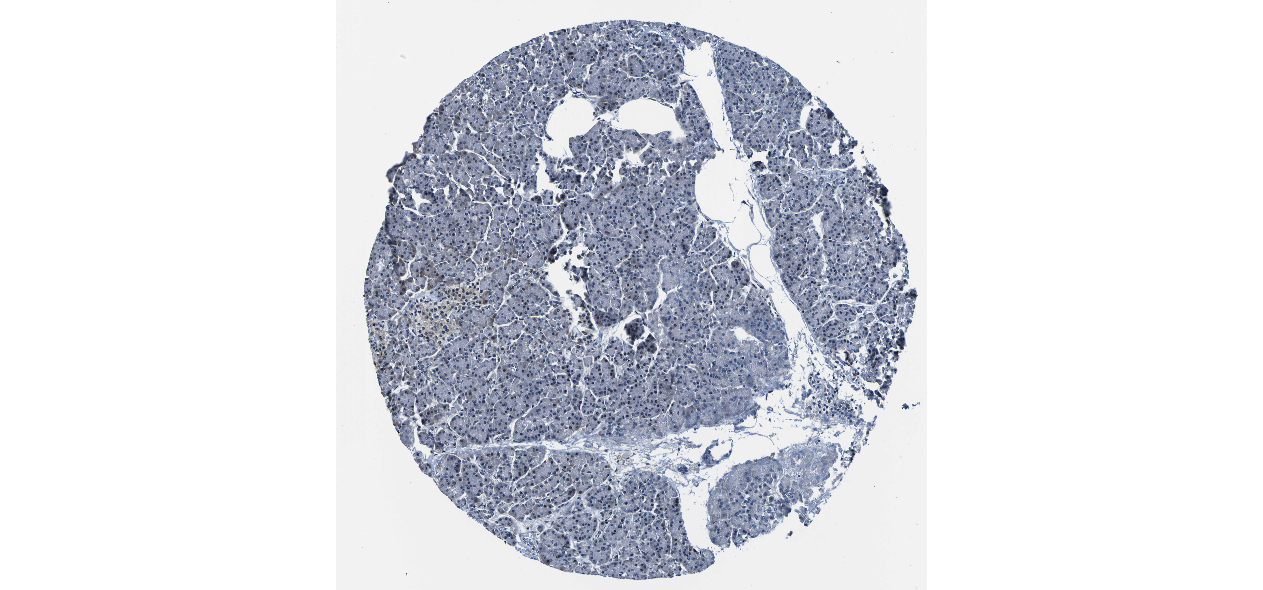

Supplement: Supplemental Information 54 [file peerj-08-10419-s054.png]

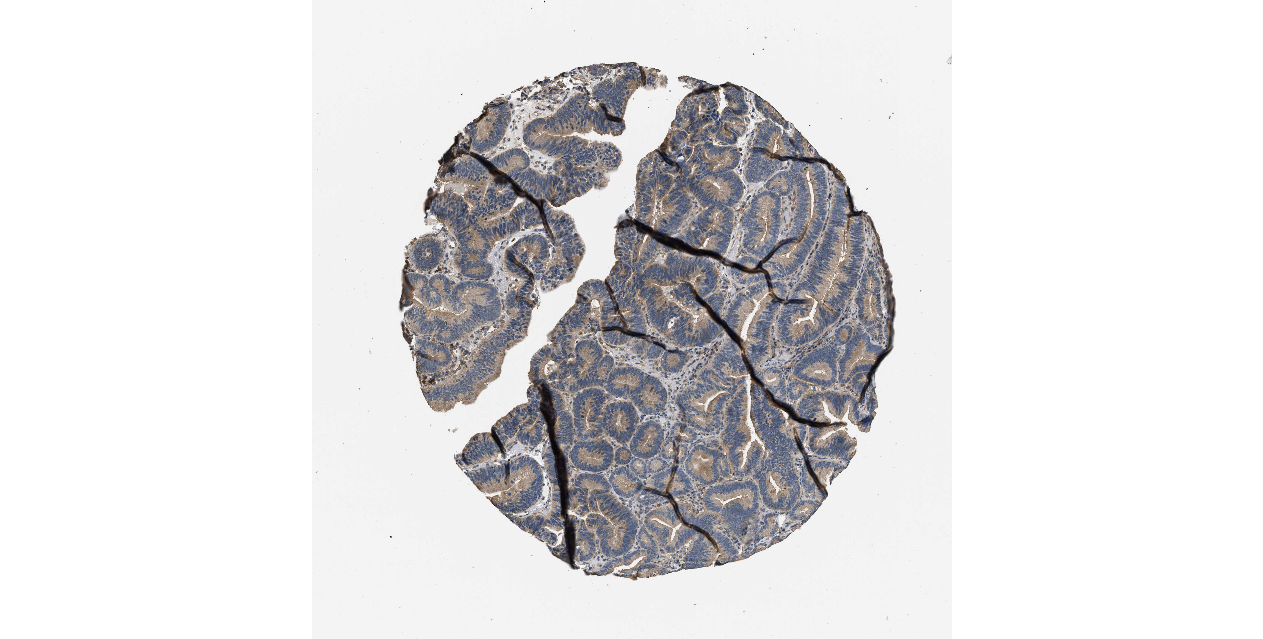

Supplement: Supplemental Information 55 [file peerj-08-10419-s055.png]

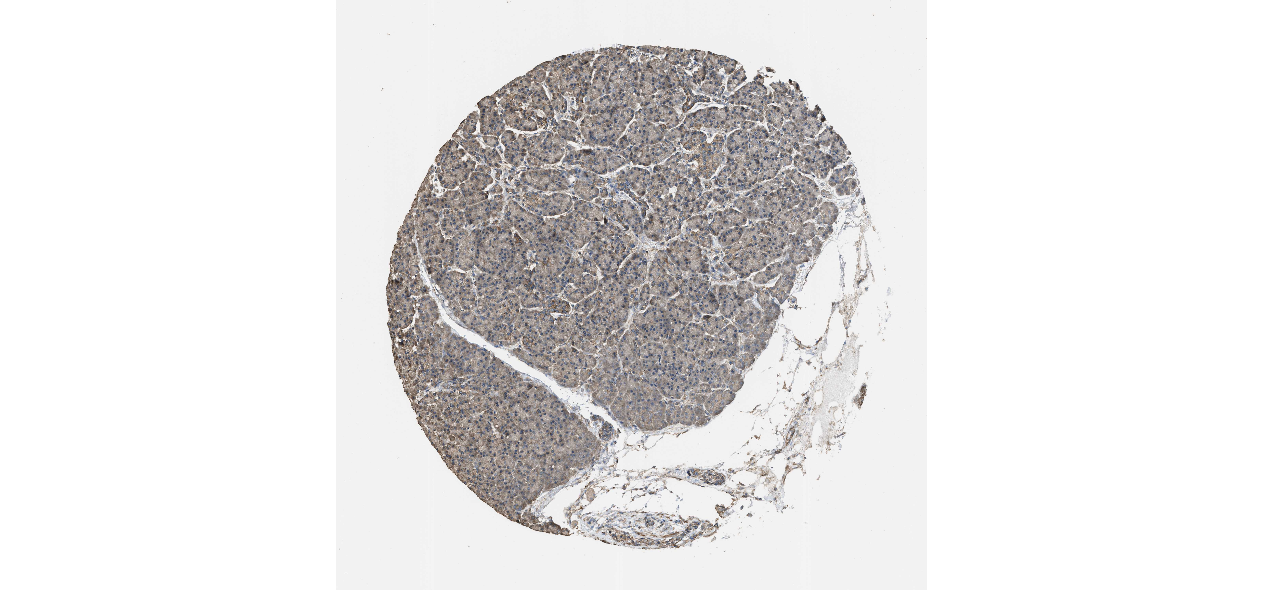

Supplement: Supplemental Information 56 [file peerj-08-10419-s056.png]

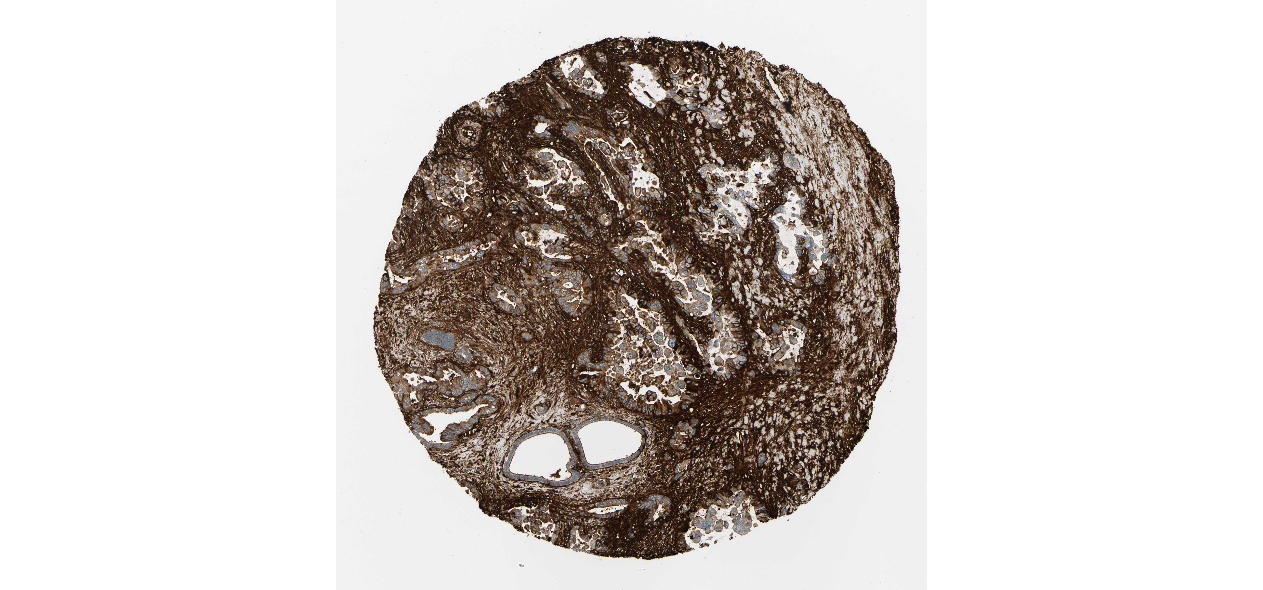

Supplement: Supplemental Information 57 [file peerj-08-10419-s057.png]

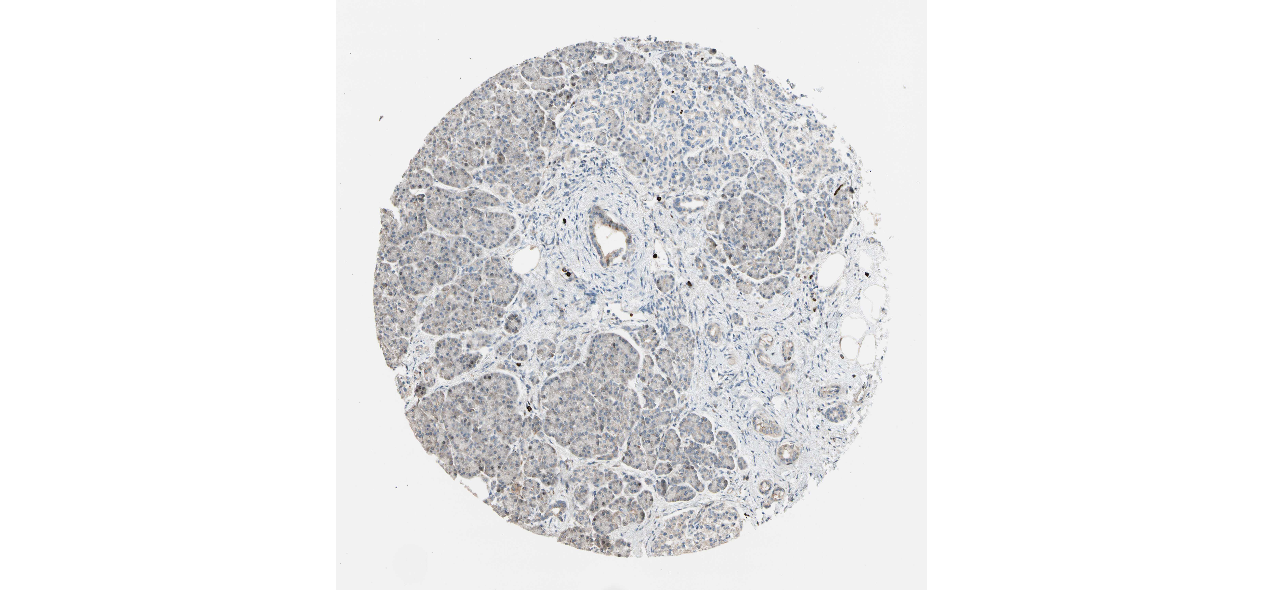

Supplement: Supplemental Information 58 [file peerj-08-10419-s058.png]

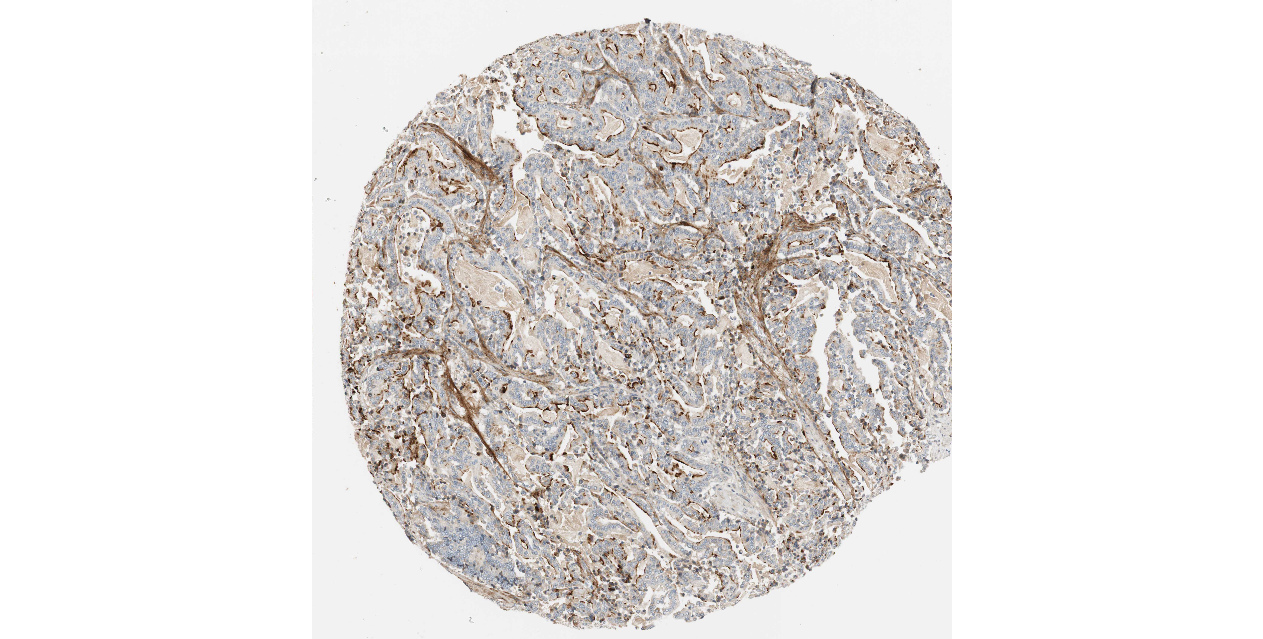

Supplement: Supplemental Information 59 [file peerj-08-10419-s059.png]

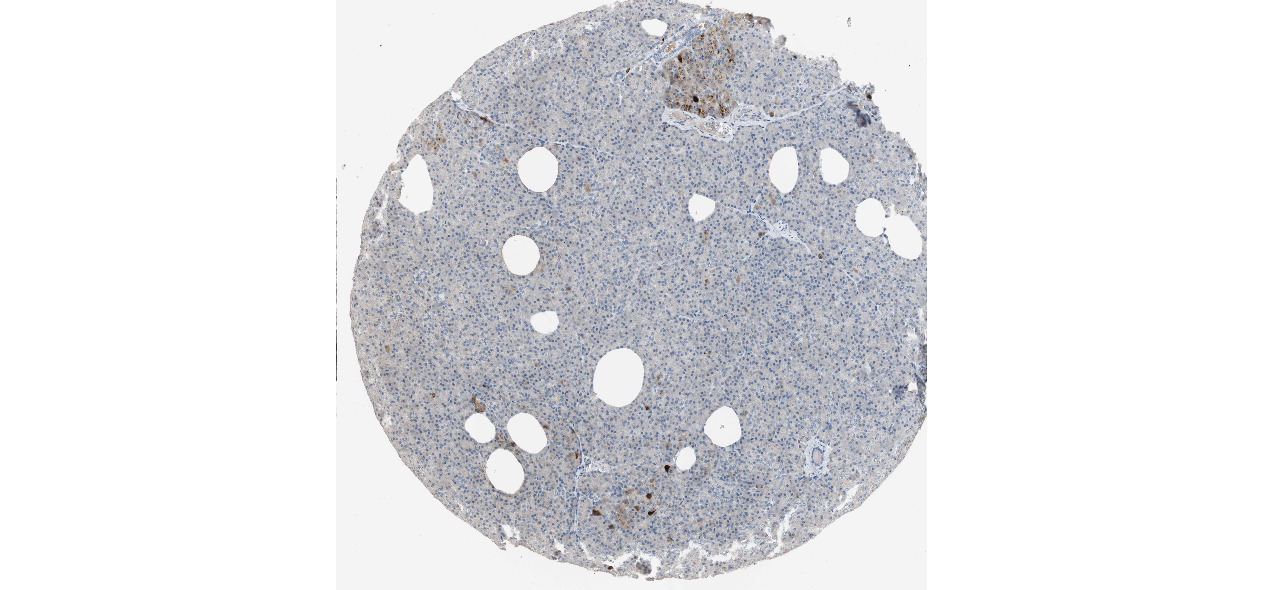

Supplement: Supplemental Information 60 [file peerj-08-10419-s060.png]

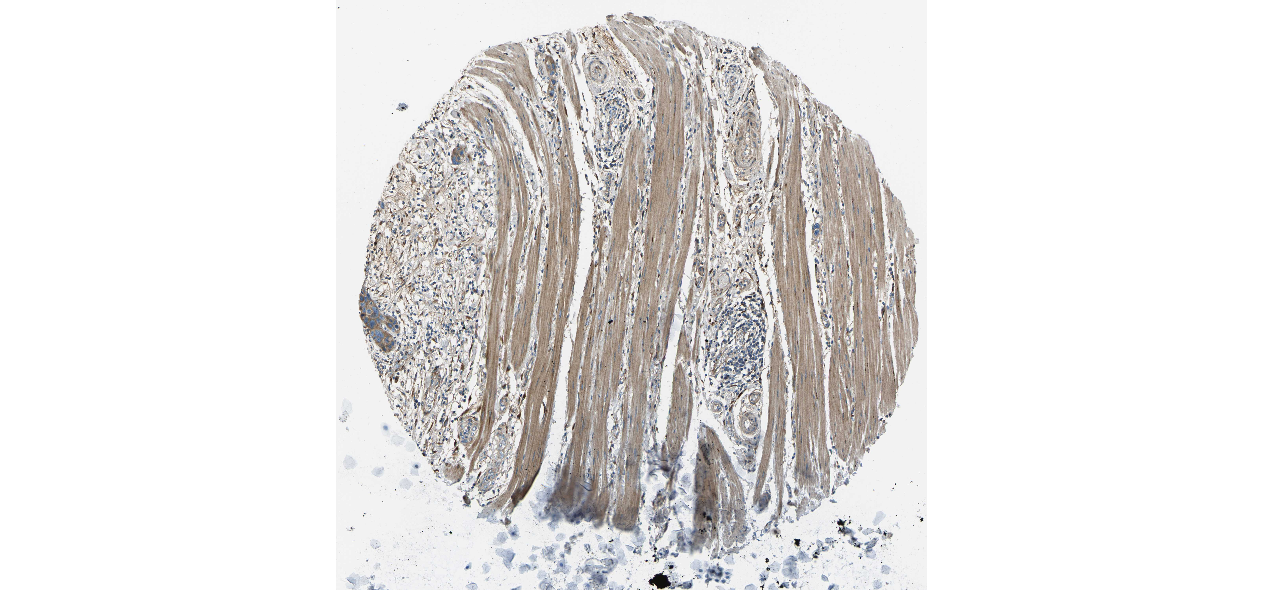

Supplement: Supplemental Information 61 [file peerj-08-10419-s061.png]

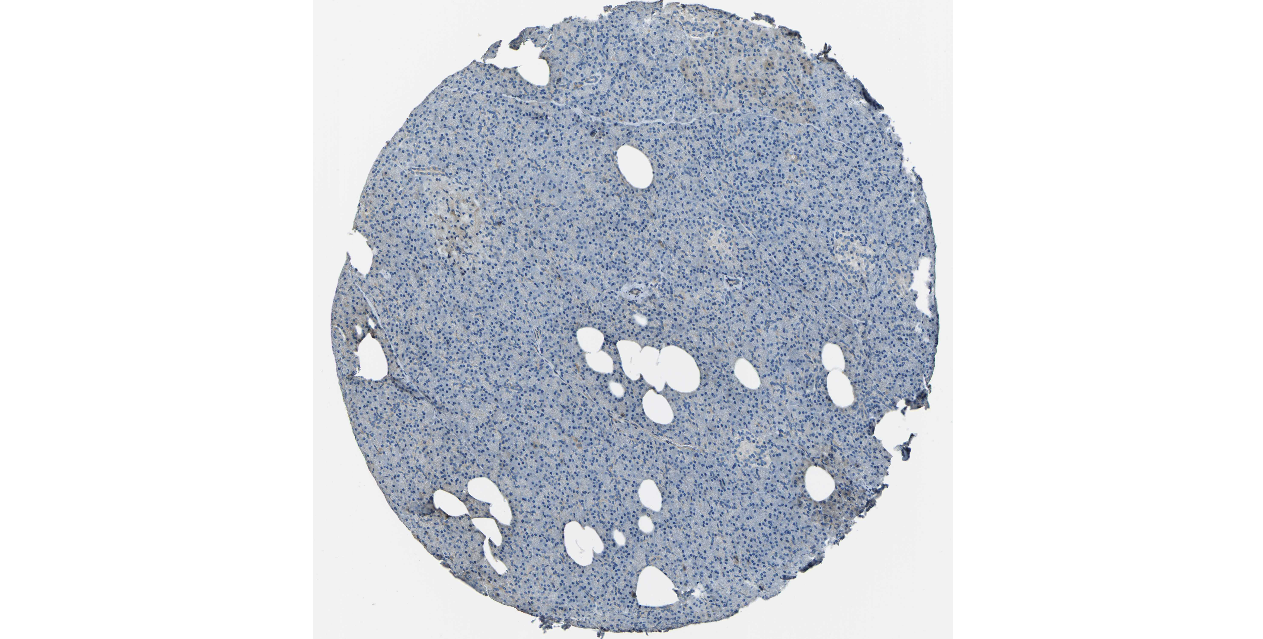

Supplement: Supplemental Information 62 [file peerj-08-10419-s062.png]

# Overall Survival

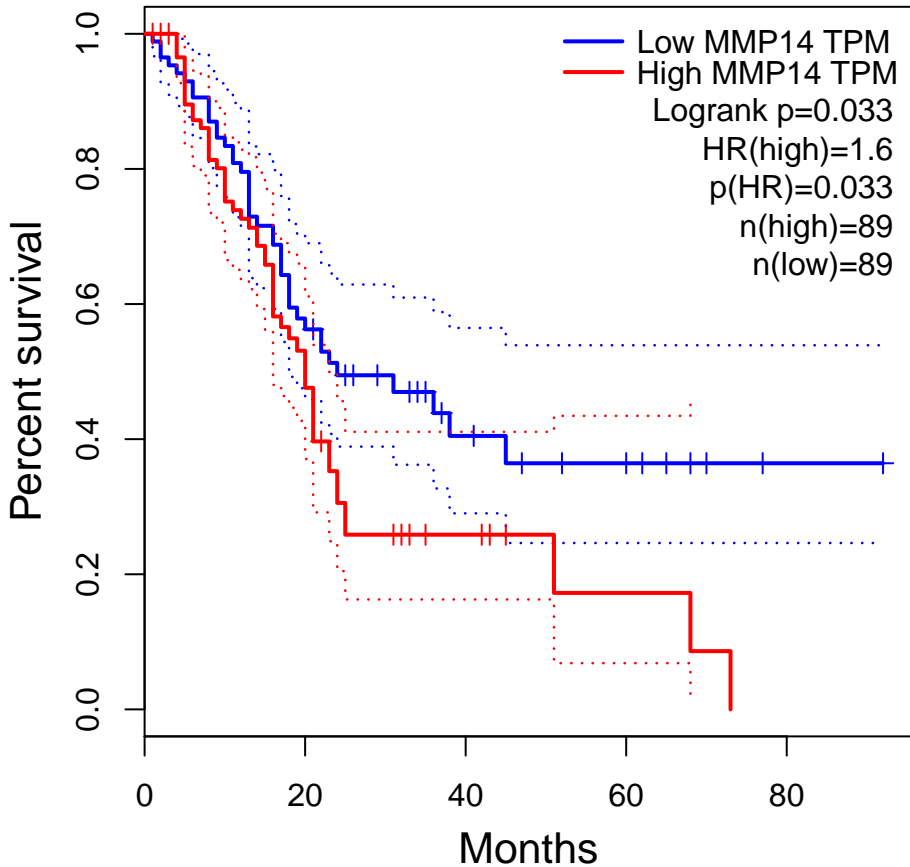

Supplement: Supplemental Information 63 [file peerj-08-10419-s063.pdf]

# Overall Survival

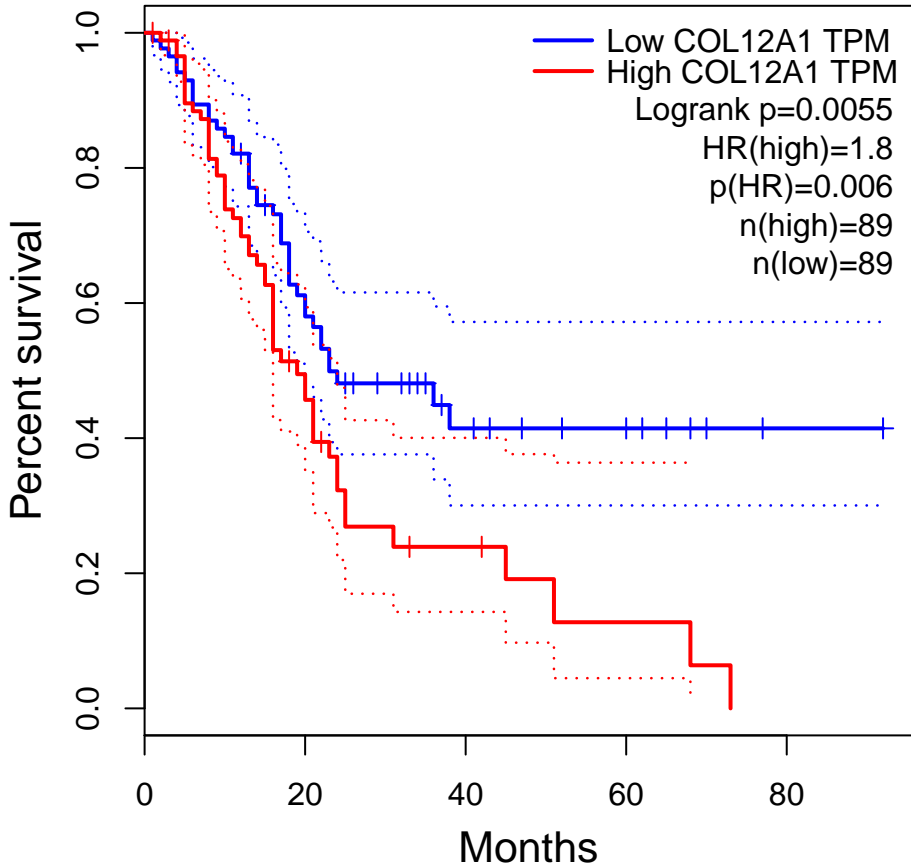

Supplement: Supplemental Information 64 [file peerj-08-10419-s064.pdf]

# Overall Survival

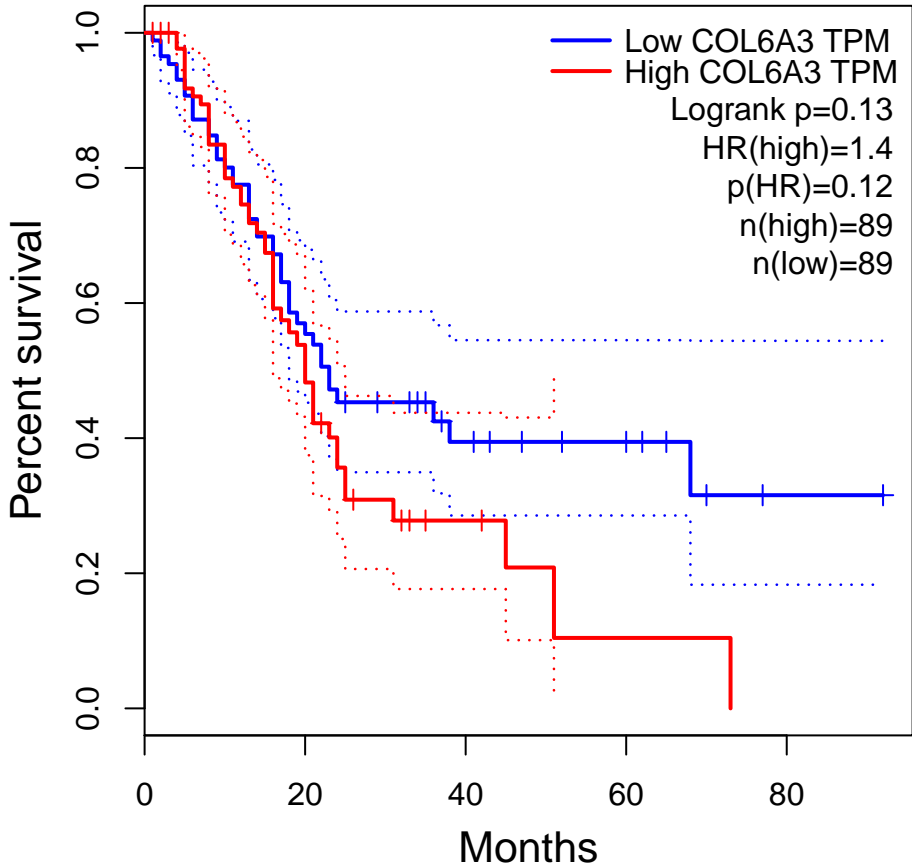

Supplement: Supplemental Information 65 [file peerj-08-10419-s065.pdf]

# Overall Survival

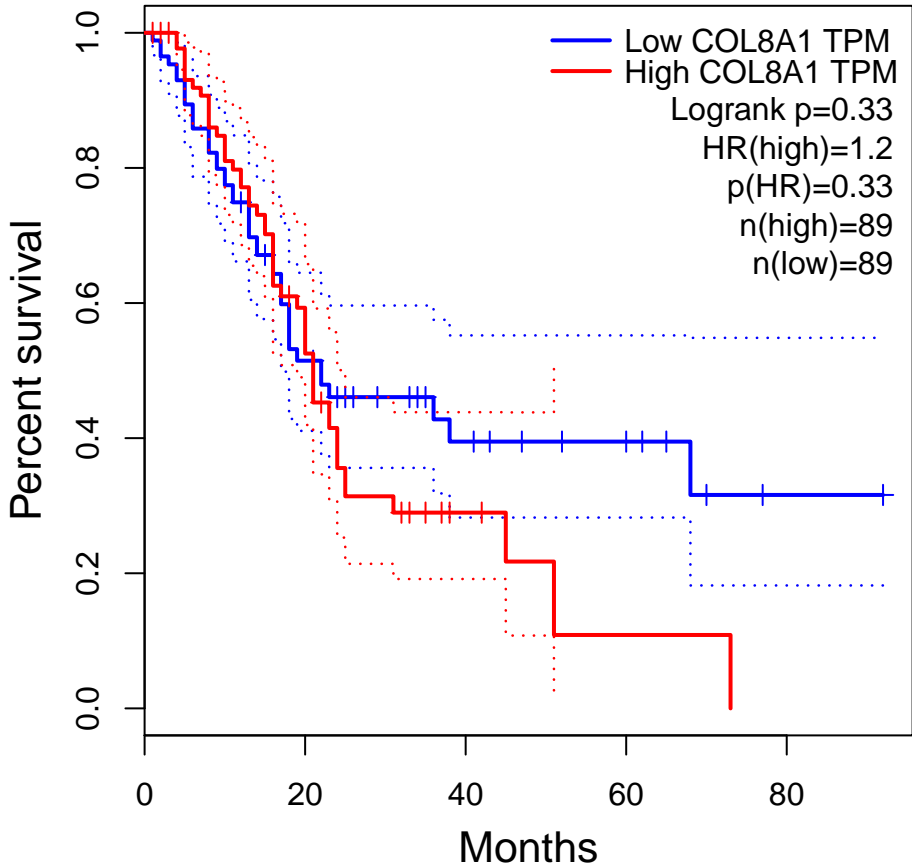

Supplement: Supplemental Information 66 [file peerj-08-10419-s066.pdf]

# Overall Survival

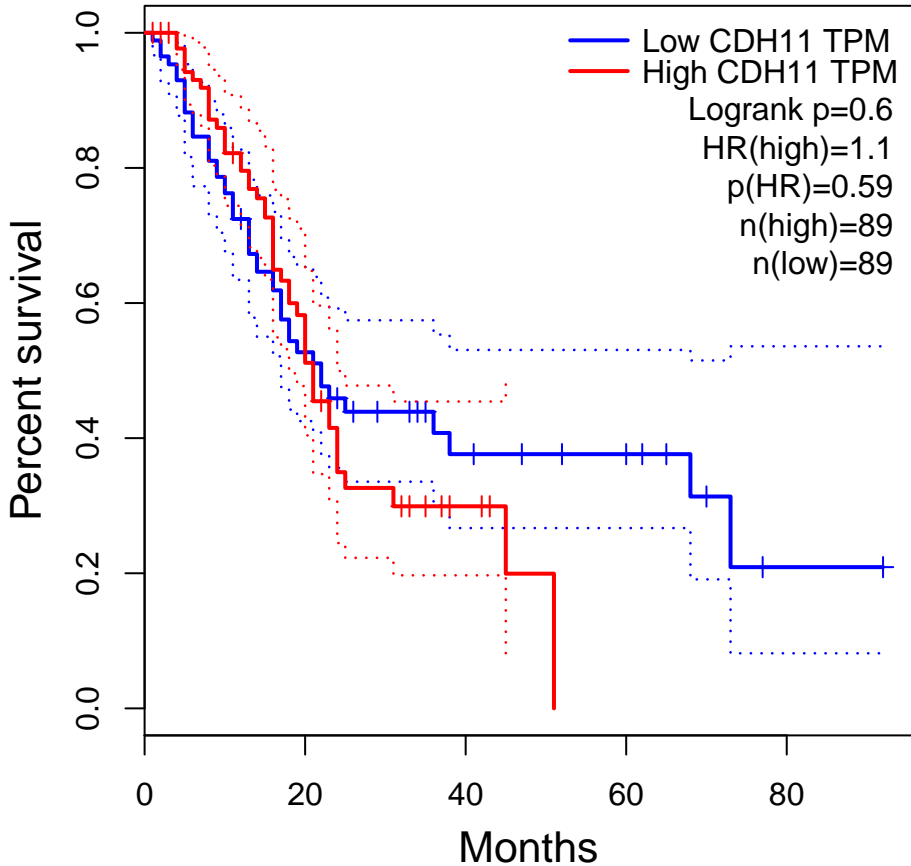

Supplement: Supplemental Information 67 [file peerj-08-10419-s067.pdf]

# Overall Survival

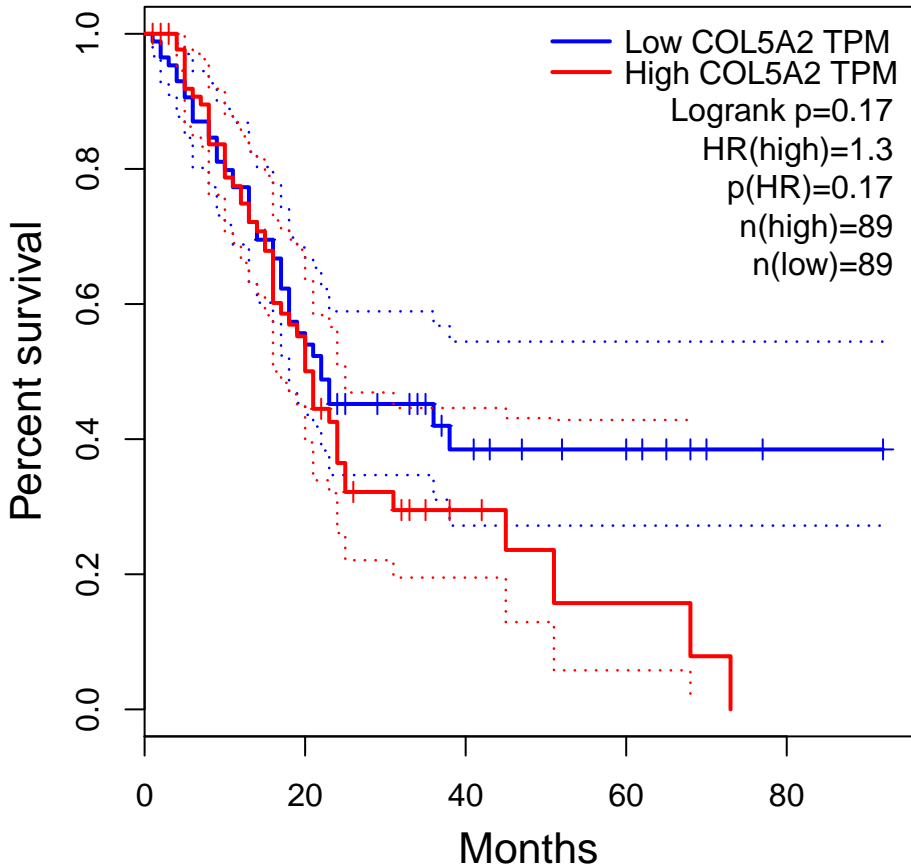

Supplement: Supplemental Information 68 [file peerj-08-10419-s068.pdf]

# Overall Survival

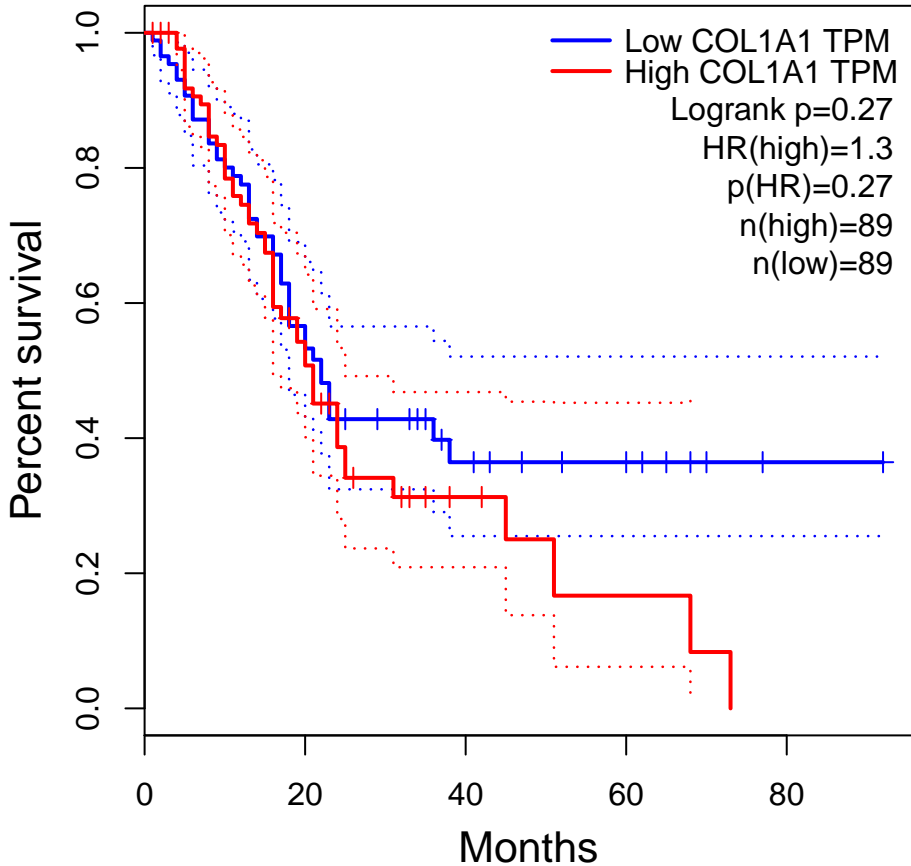

Supplement: Supplemental Information 69 [file peerj-08-10419-s069.pdf]

# Overall Survival

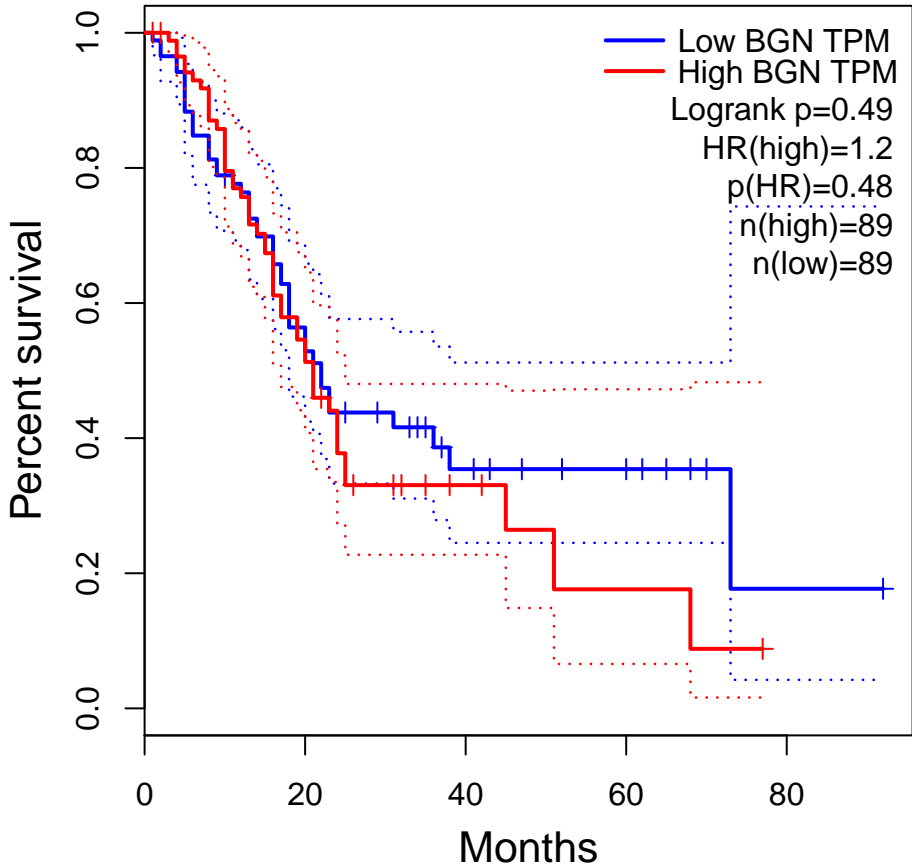

Supplement: Supplemental Information 70 [file peerj-08-10419-s070.pdf]

# Overall Survival

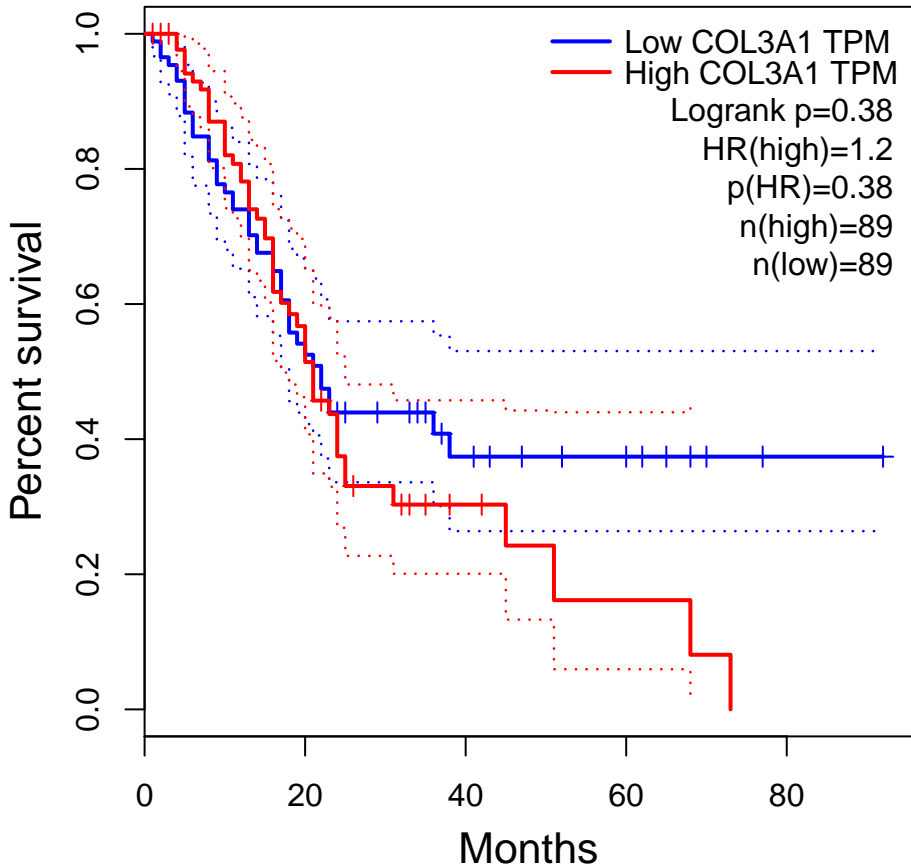

Supplement: Supplemental Information 71 [file peerj-08-10419-s071.pdf]

# Overall Survival

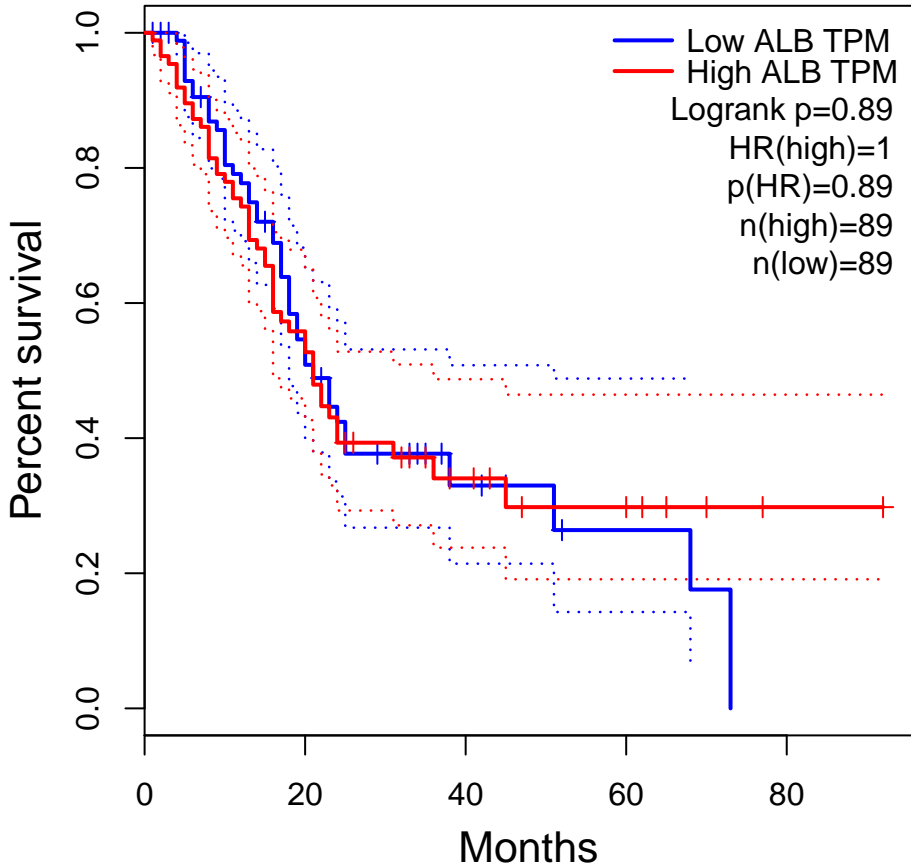

Supplement: Supplemental Information 72 [file peerj-08-10419-s072.pdf]

# Overall Survival

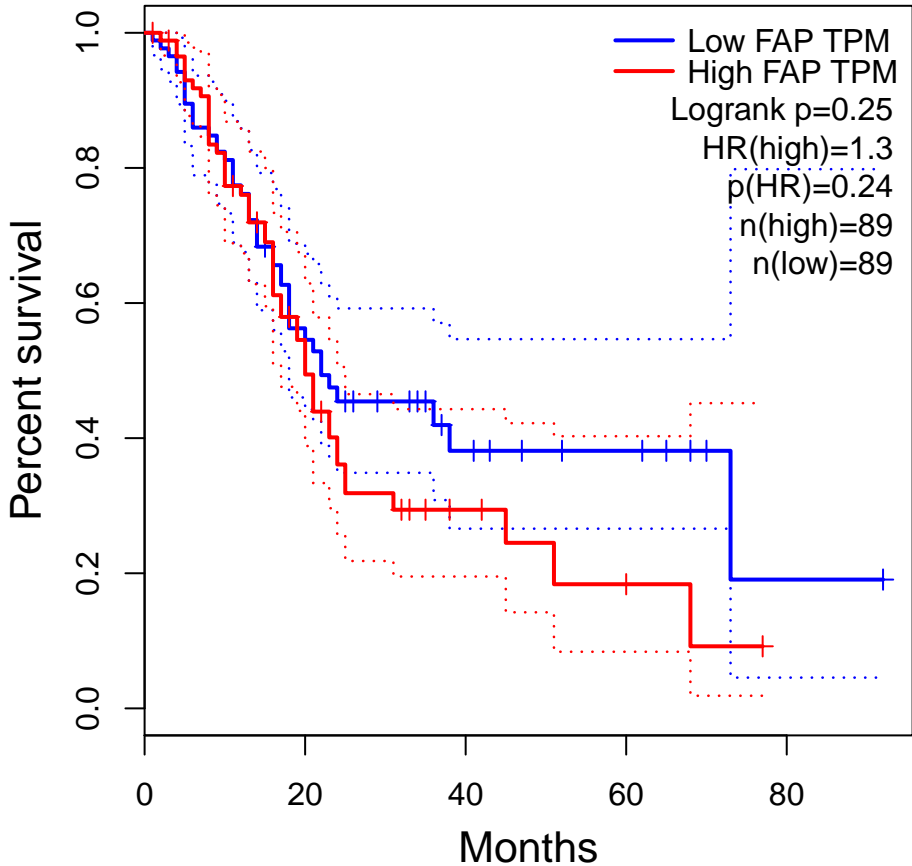

Supplement: Supplemental Information 73 [file peerj-08-10419-s073.pdf]

# Overall Survival

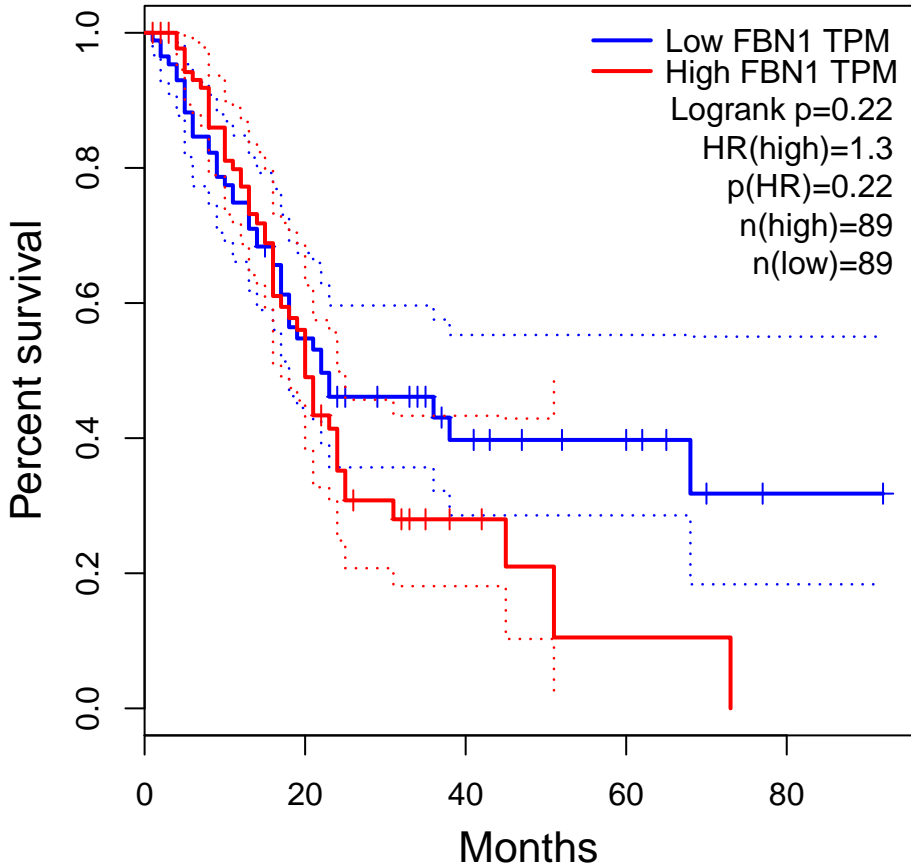

Supplement: Supplemental Information 74 [file peerj-08-10419-s074.pdf]

# Overall Survival

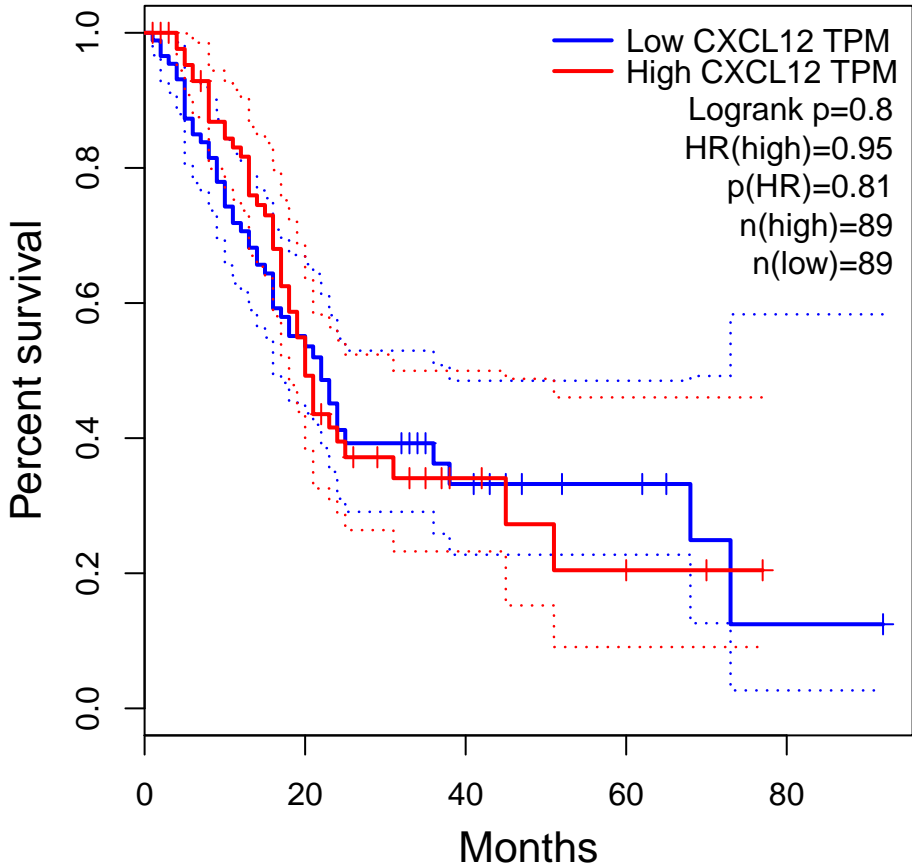

Supplement: Supplemental Information 75 [file peerj-08-10419-s075.pdf]

# Overall Survival

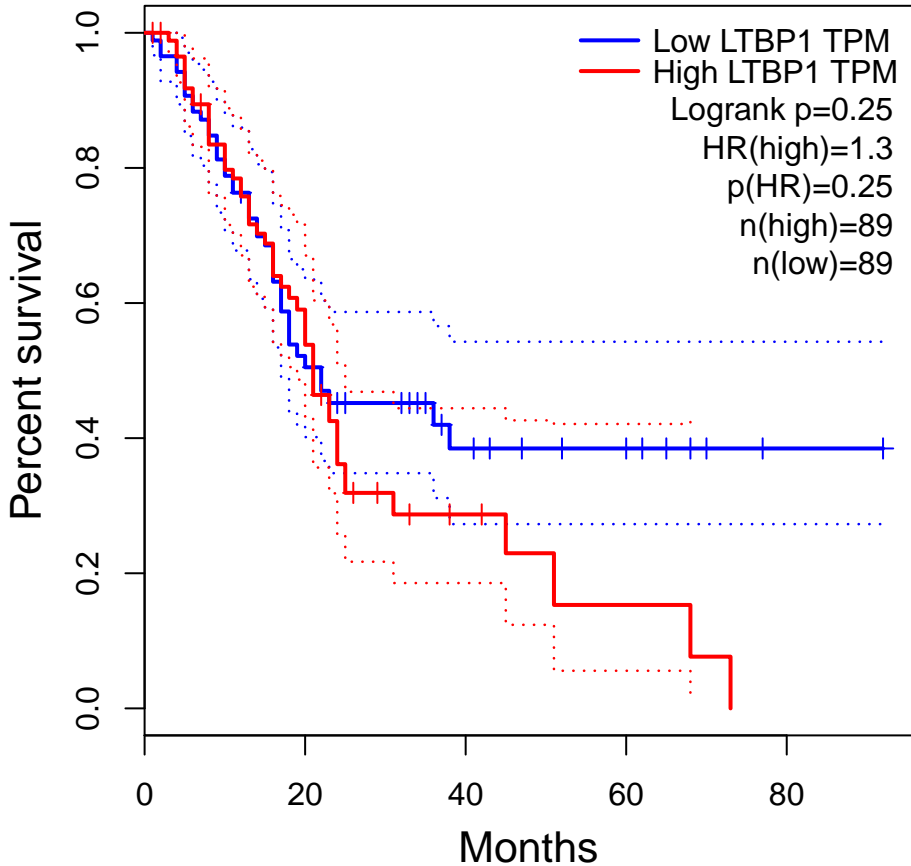

Supplement: Supplemental Information 76 [file peerj-08-10419-s076.pdf]

# Overall Survival

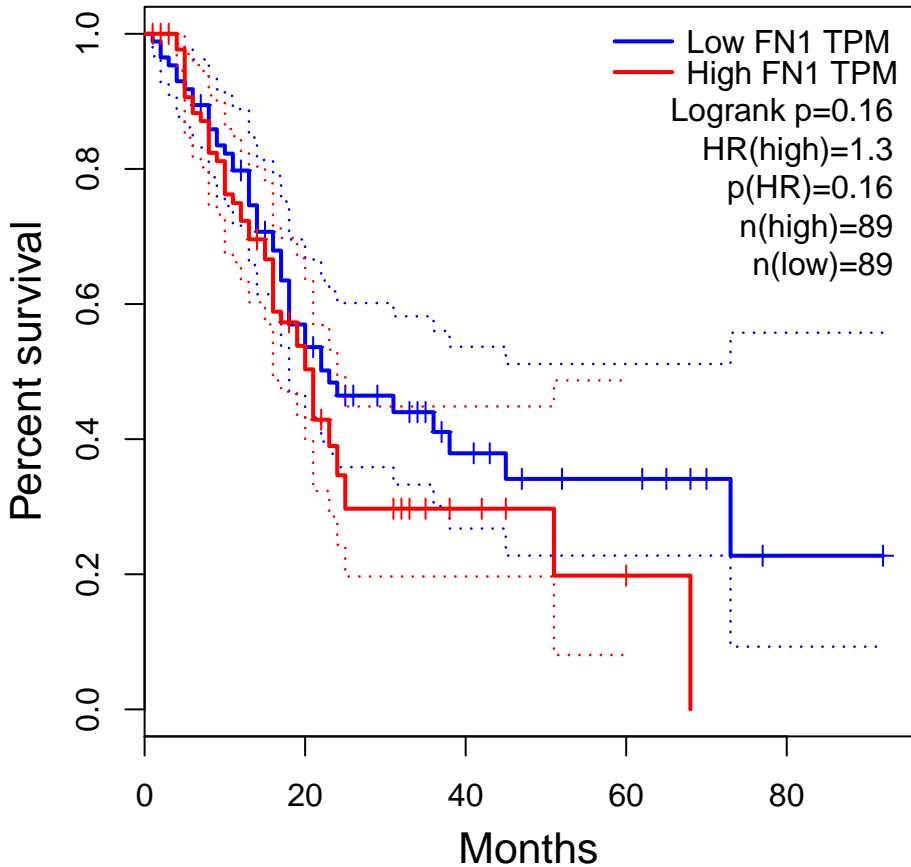

Supplement: Supplemental Information 77 [file peerj-08-10419-s077.pdf]

# Overall Survival

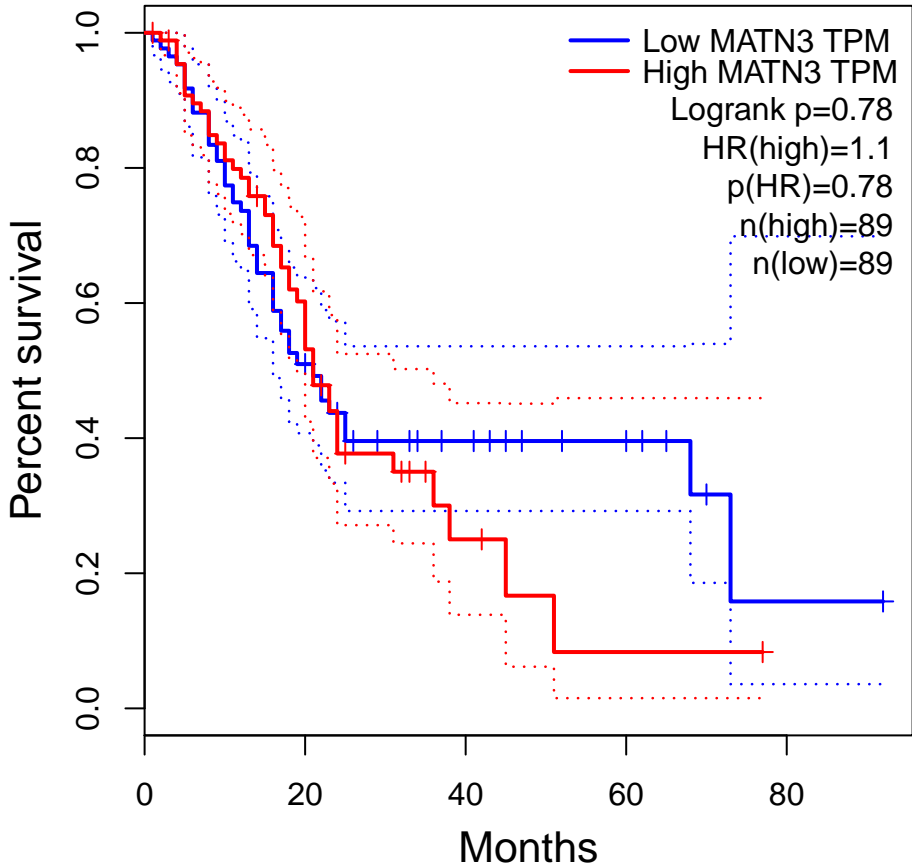

Supplement: Supplemental Information 78 [file peerj-08-10419-s078.pdf]

# Overall Survival

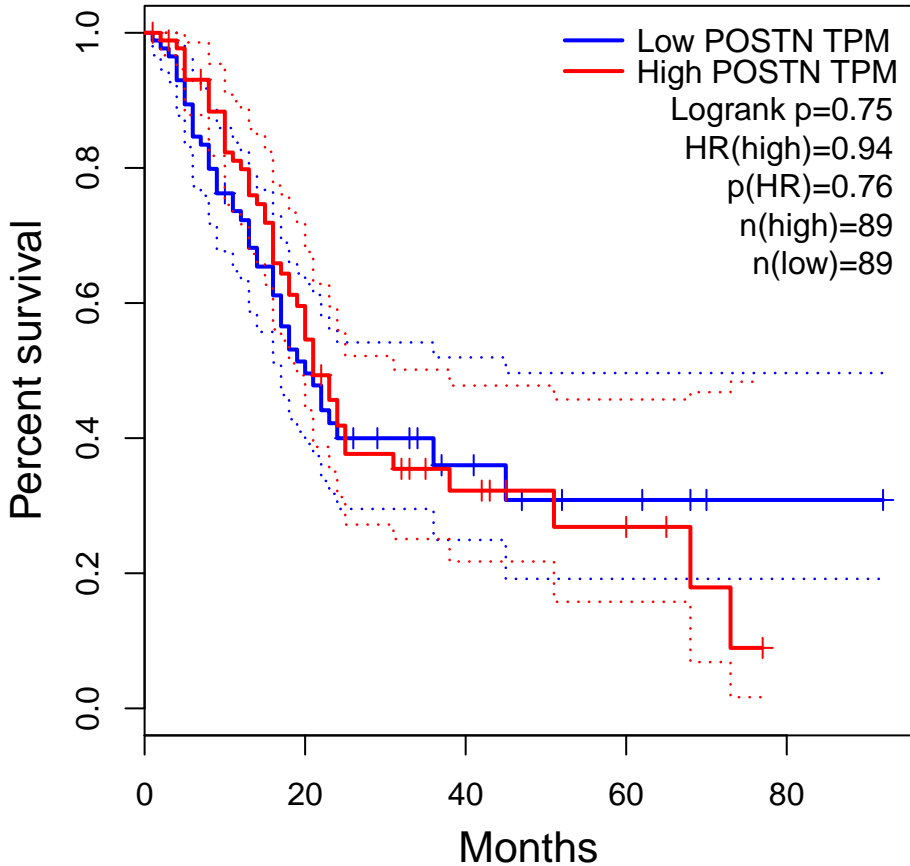

Supplement: Supplemental Information 79 [file peerj-08-10419-s079.pdf]

# Overall Survival

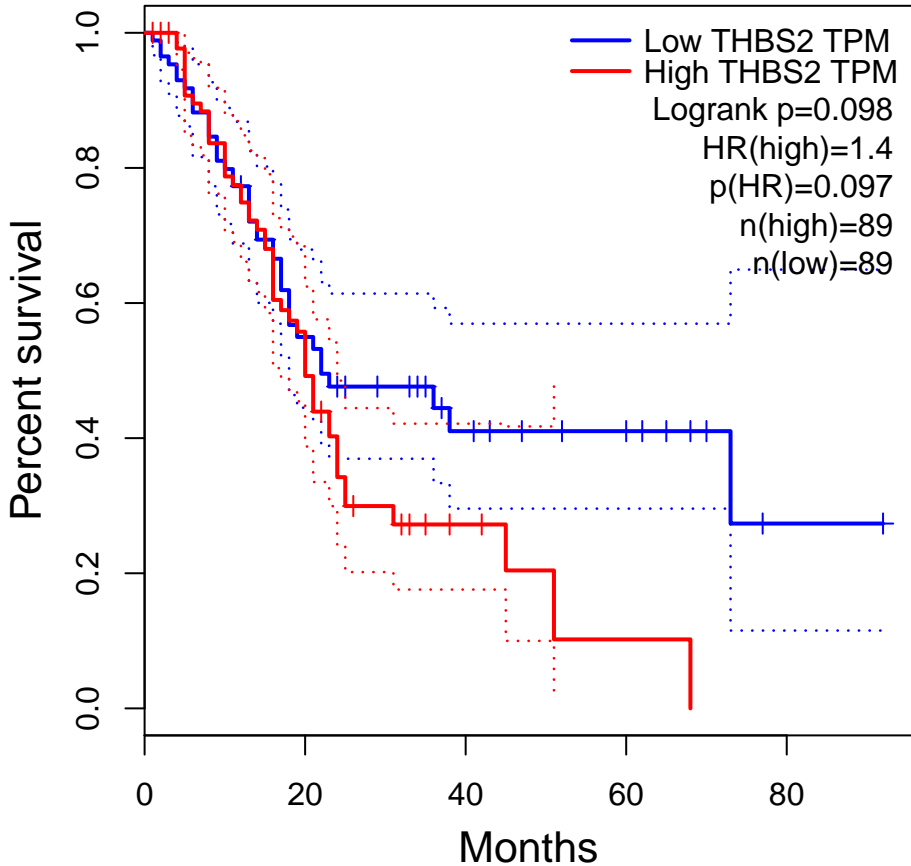

Supplement: Supplemental Information 80 [file peerj-08-10419-s080.pdf]

# Overall Survival

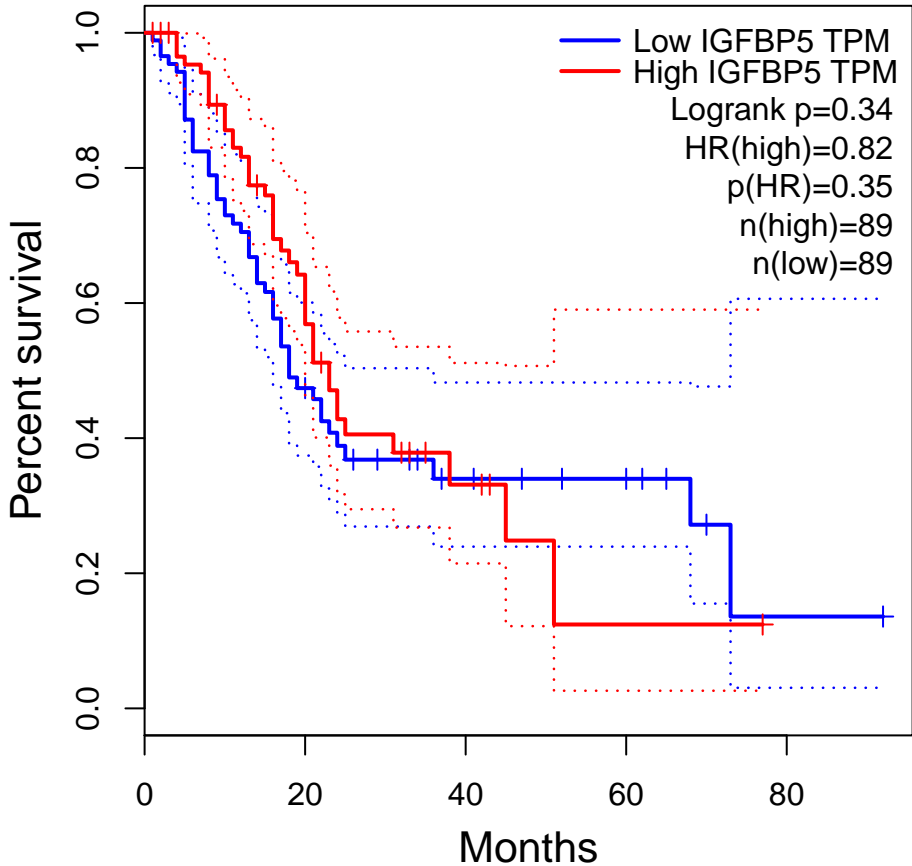

Supplement: Supplemental Information 81 [file peerj-08-10419-s081.pdf]

# Overall Survival

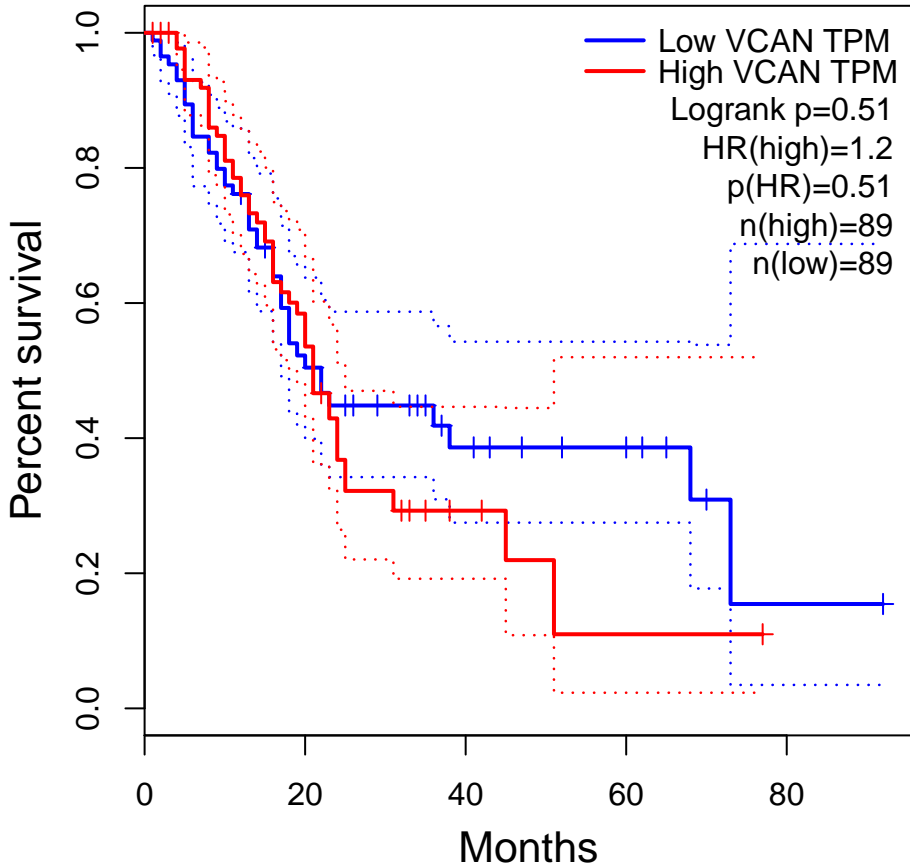

Supplement: Supplemental Information 82 [file peerj-08-10419-s082.pdf]
